# Once-daily dolutegravir-based antiretroviral therapy in infants and children living with HIV from age 4 weeks: results from the below 14 kg cohort in the randomised ODYSSEY trial

**DOI:** 10.1016/S2352-3018(22)00163-1

**Published:** 2022-08-30

**Authors:** Pauline Amuge, Abbas Lugemwa, Ben Wynne, Hilda A Mujuru, Avy Violari, Cissy M Kityo, Moherndran Archary, Ebrahim Variava, Ellen White, Rebecca M Turner, Clare Shakeshaft, Shabinah Ali, Kusum J Nathoo, Lorna Atwine, Afaaf Liberty, Dickson Bbuye, Elizabeth Kaudha, Rosie Mngqibisa, Modehei Mosala, Vivian Mumbiro, Annet Nanduudu, Rogers Ankunda, Lindiwe Maseko, Adeodata R Kekitiinwa, Carlo Giaquinto, Pablo Rojo, Diana M Gibb, Anna Turkova, Deborah Ford, ODYSSEY Trial Team, Amina Farhana Mehar (nee Abdulla), Pattamukkil Abraham, Elaine Abrams, Judith Acero, Gerald Muzorah Agaba, Grace Ahimbisibwe, Barbara Ainebyoona, Winnie Akobye, Yasmeen Akhalwaya, Nazim Akoojee, Shabinah S. Ali, Pauline Amuge, Catherine Andrea, Maria Angeles Muñoz Fernandez, Rogers Ankunda, Diana Antonia Rutebarika, Suvaporn Anugulruengkitt, Tsitsi Apollo, Moherndran Archary, Ronelle Arendze, Juliet Ategeka, Eunice Atim, Lorna Atwine, Abdel Babiker, Sarah Babirye, Enock Babu, Edward Bagirigomwa, Angella Baita, David Balamusani, Patsy Baliram, David Baliruno, Colin Ball, Henry Balwa, Alasdair Bamford, Srini Bandi, Dominique Barker, Linda Barlow-Mosha, Dickson Bbuye, Shazia Begum, Osee Behuhuma, Sarah Bernays, Rogers Besigye, Maria Bester, Joyline Bhiri, Davide Bilardi, Kristien Bird, Pauline Bollen, Chiara Borg, Anne-Marie Borges Da Silva, Jackie Brown, Elena Bruno, Torsak Bunupuradah, David Burger, Nomzamo Buthelezi, Mutsa Bwakura-Dangarembizi, Africanus Byaruhanga, Joanna Calvert, Petronelle Casey, Haseena Cassim, Sphiwee Cebekhulu, Sanuphong Chailert, Suwalai Chalermpantmetagul, Wanna Chamjamrat, Man Chan, Precious Chandiwana, Thannapat Chankun, Sararut Chanthaburanun, Nuttawut Chanto, Ennie Chidziva, Minenhle Chikowore, Joy Chimanzi, Dujrudee Chinwong, Stuart Chitongo, Moses Chitsamatanga, Joshua Choga, Duangrat Chutima, Polly Clayden, Alexandra Coelho, Angela Colbers, Alexandra Compagnucci, Ana Constança Mendes, Magda Conway, Mark F. Cotton, Jane Crawley, Tim R. Cressey, Jacky Crisp, Ana Cristina Matos, Sumaya Dadan, Jacqui Daglish, Siva Danaviah, Tseleng Daniel, Anita De Rossi, Sukanda Denjanta, Els Dobbels, Maria Dowie, Prosper Dube, Benedictor Dube, Nimisha Dudakia, Alice Elwana, Cristina Epalza, David Eram, Juan Erasmus, Peter Erim, Luis Escosa Garcia, Zaakirah Essack, Carolina Estepa, Monica Etima, Alexandre Fernandes, Maite Fernandez, Felicity Fitzgerald, Jacquie Flynn, Deborah Ford, Claudia Fortuny Guasch, Caroline Foster, George Fourie, Yolandie Fourie, Sophie Foxall, Derusha Frank, Kate Gandhi, India Garcia, Kathleen Gartner, Joshua Gasa, Gugu Gasa, Carlo Giaquinto, Diana M. Gibb, Coral Gomez Rico, Daniel Gomez-Pena, Secrecy Gondo, Anna Goodman, Maria Gorreti Nakalema, Winnie Gozhora, Pisut Greetanukroh, Biobanco Gregorio Maranon, Tiziana Grossele, Shamiso Gwande, Tapiwa Gwaze, Tsitsi Gwenzi, James Hakim, Emmanuel Hakiza, Abdul Hamid Kaka, Ashley Harley, Mornay Isaacs, Richard Isabirye, Wilber Ishemunyoro, Tom Jacobs, Lungile Jafta, Nasir Jamil, Anita Janse Janse van Rensburg, Vinesh Jeaven, Maria José Mellado Peña, Gonzague Jourdain, Katabalwa Juliet, Thidarat Jumpimai, Raungwit Junkaew, Thidarat Jupimai, Winfred Kaahwa, Mildred Kabasonga, Olivia Kaboggoza, Rose Jacqueline Kadhuba, Ampika Kaewbundit, Kanyanee Kaewmamueng, Bosco Kafufu, Brenda Kakayi, Phakamas Kamboua, Suparat Kanjanavanit, Gladys Kasangaki, Naruporn Kasipong, Miriam Kasozi, Hajira Kataike, Chrispus Katemba, Elizabeth Kaudha, Nkata Kekane, Adeodata R. Kekitiinwa, Edridah Keminyeto, Woottichai Khamduang, Warunee Khamjakkaew, Jiraporn Khamkon, Sasipass Khannak, Orapin Khatngam, Tassawan Khayanchoomnoom, Busi Khumalo, Mirriam Khunene, Suwimon Khusuwan, Phionah Kibalama, Robinah Kibenge, Anthony Kirabira, Cissy M. Kityo, Lameck Kiyimba, Nigel Klein, Soraya Klinprung, Robin Kobbe, Olivia Kobusingye, Josephine Kobusungye, Areerat Kongponoi, Christoph Königs, Olivier Koole, Christelle Kouakam, Nitinart Krueduangkam, Namthip Kruenual, Nuananong Kunjaroenrut, Raymonds Kyambadde, Priscilla Kyobutungi, Flavia Kyomuhendo, Erinah Kyomukama, Reshma Lakha, Cleopatra Langa, Laddawan Laomanit, Emily Lebotsa, Prattana Leenasirimakul, Lawrence Lekku, Sarah Lensen, Valériane Leroy, Jin Li, Afaaf Liberty, Juthamas Limplertjareanwanich, Emma Little, Abbas Lugemwa, Ezra Lutalo, Jose Luis Jimenez, Hermione Lyall, Candice MacDonald, Gladness Machache, Penelope Madlala, Tryphina Madonsela, Nomfundo Maduna, Joel Maena, Apicha Mahanontharit, Collin Makanga, Candice Makola, Shafic Makumbi, Lucille Malgraaf, Angelous Mamiane, Felicia Mantkowski, Wendy Mapfumo, Laura Marques, Agnes Mary Mugagga, Lindiwe Maseko, Tshepiso Masienyane, Ruth Mathiba, Farai Matimba, Sajeeda Mawlana, Emmanuel Mayanja, Fatima Mayat, Ritah Mbabazi, Nokuthula Mbadaliga, Faith Mbasani, Kathleen McClaughlin, Helen McIlleron, Watchara Meethaisong, Patricia Mendez Garcia, Annet Miwanda, Carlota Miranda, Siphiwe Mkhize, Kgosimang Mmolawa, Rosie Mngqibisa, Fatima Mohamed, Tumelo Moloantoa, Maletsatsi Monametsi, Samuel Montero, Cecilia L. Moore, Rejoice Mosia, Columbus Moyo, Mumsy Mthethwa, Shepherd Mudzingwa, Tawona Mudzviti, Hilda Mujuru, Emmanuel Mujyambere, Trust Mukanganiki, Cynthia Mukisa Williams, Mark Mulder, Disan Mulima, Alice Mulindwa, Vivian Mumbiro, Zivai Mupambireyi, Alba Murciano Cabeza, Herbert Murungi, Dorothy Murungu, Sandra Musarurwa, Victor Musiime, Alex V. Musiime, Maria Musisi, Philippa Musoke, Barbara Musoke Nakirya, Godfrey Musoro, Sharif Musumba, Sobia Mustafa, Shirley Mutsai, Phyllis Mwesigwa Rubondo, Mariam Naabalamba, Immaculate Nagawa, Allemah Naidoo, Shamim Nakabuye, Sarah Nakabuye, Sarah Nakalanzi, Justine Nalubwama, Annet Nalugo, Stella Nalusiba, Clementine Namajja, Sylvia Namanda, Paula Namayanja, Esther Nambi, Rachael Kikabi Namuddu, Stella Namukwaya, Florence Namuli, Josephine Namusanje, Rosemary Namwanje, Anusha Nanan-kanjee, Annet Nanduudu, Charity Nankunda, Joanita Nankya Baddokwaya, Maria Nannungi, Winnie Nansamba, Kesdao Nanthapisal, Juliet Nanyonjo, Sathaporn Na-Rajsima, Claire Nasaazi, Helena Nascimento, Eleni Nastouli, Wipaporn Natalie Songtaweesin, Kusum Nathoo, Ian Natuhurira, Rashidah Nazzinda, Thabisa Ncgaba, Milly Ndigendawani, Makhosonke Ndlovu, Georgina Nentsa, Chaiwat Ngampiyaskul, Ntombenhle Ngcobo, Nicole Ngo Giang Huong, Pia Ngwaru, Ruth Nhema, Emily Ninsiima, Gloria Ninsiima, Misheck Nkalo Phiri, Antoni Noguera Julian, Monica Nolan, Thornthun Noppakaorattanamanee, Muzamil Nsibuka Kisekka, Eniola Nsirim, Rashina Nundlal, Rosita Nunes, Lungile Nyantsa, Mandisa Nyati, Sean O'Riordan, Paul Ocitti Labeja, Denis Odoch, Rachel Oguntimehin, Martin Ojok, Geoffrey Onen, Wilma Orange, Pradthana Ounchanum, Benson Ouma, Andreia Padrao, Deborah Pako, Anna Parker, Malgorzata Pasko-Szcech, Reena Patel, Rukchanok Peongjakta, Turian Petpranee, Tasmin Phillips, Jackie Philps, Laura Picault, Sonja Pieterse, Helena Pinheiro, Supawadee Pongprapass, Anton Pozniak, Andrew Prendergast, Luis Prieto Tato, Patcharee Puangmalai, Thanyawee Puthanakit, Modiehi Rakgokong, Helena Ramos, Nastassja Ramsagar, Cornelius Rau, Yoann Riault, Pablo Rojo Conejo, Basiimwa Roy Clark, Eddie Rubanga, Baker Rubinga, Chutima Ruklao, Pattira Runarassamee, Diana Antonia Rutebarika, Chalermpong Saenjum, Chayakorn Saewtrakool, Yacine Saidi, Talia Sainz Costa, Chutima Saisaengjan, Rebecca Sakwa, Tatiana Sarfati, Noshalaza Sbisi, Dihedile Scheppers, Stephan Schultze-Strasser, Ulf Schulze-Sturm, Karen Scott, Janet Seeley, Robert Serunjogi, Leora Sewnarain, Clare Shakeshaft, Subashinie Sidhoo, Mercy Shibemba, Delane Shingadia, Sheleika Singh, Wasna Sirirungsi, Sibongile Sithebe, Theresa Smit, Kurt Smith, Marlize Smuts, Moira Spyer, Worathip Sripaoraya, Ussanee Srirompotong, Warunee Srisuk, Mark Ssenyonga, Patamawadee Sudsaard, Praornsuda Sukrakanchana, Pathanee Tearsansern, Carla Teixeira, Kanchana Than-in-at, Thitiwat Thapwai, Yupawan Thaweesombat, Jutarat Thewsoongnoen, Rodolphe Thiébaut, Margaret Thomason, Laura Thrasyvoulou, Khanungnit Thungkham, Judith Tikabibamu, Gloria Tinago, Ketmookda Trairat, Gareth Tudor-Williams, Mercy Tukamushaba, Deogratiuos Tukwasibwe, Julius Tumusiime, Joana Tuna, Anna Turkova, Rebecca Turner, Arttasid Udomvised, Aasia Vadee, Hesti Van Huyssteen, Nadine Van Looy, Ebrahim Variava, Yvonne Vaughan-Gordon, Giulio Vecchia, Avy Violari, Richard Vowden, Hylke Waalewijn, Rebecca Wampamba, Steve Welch, Ian Weller, Sibusisiwe Weza, Ellen White, Ian White, Kaja Widuch, Helen Wilkes, Sookpanee Wimonklang, Ben Wynne, Pacharaporn Yingyong, Zaam Zinda Nakawungu, Peter Zuidewind

**Affiliations:** a Baylor College of Medicine Children's Foundation-Uganda, Kampala, Uganda; b Joint Clinical Research Centre, Mbarara, Uganda; c Medical Research Council Clinical Trials Unit at University College London, London, UK; d University of Zimbabwe Clinical Research Centre, Harare, Zimbabwe; e Perinatal HIV Research Unit, University of the Witwatersrand, South Africa; f Joint Clinical Research Centre, Kampala, Uganda; g Department of Paediatrics and Children Health, King Edward VIII Hospital, University of KwaZulu-Natal, Durban, South Africa; h Department of Women and Child Health, University of Padova, Italy; i Penta Foundation, Padova, Italy; j Pediatric Infectious Diseases Unit, Hospital 12 de Octubre, Madrid, Spain

## Abstract

**Background:**

Young children living with HIV have few treatment options. We aimed to assess the efficacy and safety of dolutegravir-based antiretroviral therapy (ART) in children weighing between 3 kg and less than 14 kg.

**Methods:**

ODYSSEY is an open-label, randomised, non-inferiority trial (10% margin) comparing dolutegravir-based ART with standard of care and comprises two cohorts (children weighing ≥14 kg and <14 kg). Children weighing less than 14 kg starting first-line or second-line ART were enrolled in seven HIV treatment centres in South Africa, Uganda, and Zimbabwe. Randomisation, which was computer generated by the trial statistician, was stratified by first-line or second-line ART and three weight bands. Dispersible 5 mg dolutegravir was dosed according to WHO weight bands. The primary outcome was the Kaplan-Meier estimated proportion of children with virological or clinical failure by 96 weeks, defined as: confirmed viral load of at least 400 copies per mL after week 36; absence of virological suppression by 24 weeks followed by a switch to second-line or third-line ART; all-cause death; or a new or recurrent WHO stage 4 or severe WHO stage 3 event. The primary outcome was assessed by intention to treat in all randomly assigned participants. A primary Bayesian analysis of the difference in the proportion of children meeting the primary outcome between treatment groups incorporated evidence from the higher weight cohort (≥14 kg) in a prior distribution. A frequentist analysis was also done of the lower weight cohort (<14 kg) alone. Safety analyses are presented for all randomly assigned children in this study (<14 kg cohort). ODYSSEY is registered with ClinicalTrials.gov, NCT02259127.

**Findings:**

Between July 5, 2018, and Aug 26, 2019, 85 children weighing less than 14 kg were randomly assigned to receive dolutegravir (n=42) or standard of care (n=43; 32 [74%] receiving protease inhibitor-based ART). Median age was 1·4 years (IQR 0·6–2·0) and median weight 8·1 kg (5·4–10·0). 72 (85%) children started first-line ART and 13 (15%) started second-line ART. Median follow-up was 124 weeks (112–137). By 96 weeks, treatment failure occurred in 12 children in the dolutegravir group (Kaplan-Meier estimated proportion 31%) versus 21 (48%) in the standard-of-care group. The Bayesian estimated difference in treatment failure (dolutegravir minus standard of care) was –10% (95% CI –19% to –2%; p=0·020), demonstrating superiority of dolutegravir. The frequentist estimated difference was –18% (–36% to 2%; p=0·057). 15 serious adverse events were reported in 11 (26%) children in the dolutegravir group, including two deaths, and 19 were reported in 11 (26%) children in the standard-of-care group, including four deaths (hazard ratio [HR] 1·08 [95% CI 0·47–2·49]; p=0·86). 36 adverse events of grade 3 or higher were reported in 19 (45%) children in the dolutegravir group, versus 34 events in 21 (49%) children in the standard-of-care group (HR 0·93 [0·50–1·74]; p=0·83). No events were considered related to dolutegravir.

**Interpretation:**

Dolutegravir-based ART was superior to standard of care (mainly protease inhibitor-based) with a lower risk of treatment failure in infants and young children, providing support for global dispersible dolutegravir roll-out for younger children and allowing alignment of adult and paediatric treatment.

**Funding:**

Paediatric European Network for Treatment of AIDS Foundation, ViiV Healthcare, UK Medical Research Council.

## Introduction

In 2021, 1·7 million children younger than 15 years were living with HIV globally, of whom 84% were living in sub-Saharan Africa.^1^ Despite the global campaign to eliminate vertical transmission, children still acquire HIV during pregnancy, delivery, or breastfeeding, with 160 000 new infections globally in 2021.^1^ Treatment for children living with HIV aims to achieve and maintain undetectable viral loads, with correspondingly low viral reservoirs, and to provide long-term health and high quality of life. Achieving high adherence to treatment in infants and children living with HIV is challenging due to complexities in administering antiretroviral therapy (ART) and few treatment options. Paediatric ritonavir-boosted lopinavir, which was recommended by WHO for children younger than 3 years (2016) and younger than 6 years (2018),^2^, ^3^ requires administration twice a day, is poorly palatable in liquid or pellet form, and interacts with rifampicin making it difficult to use for children co-infected with tuberculosis. Furthermore, single-dose ritonavir (needed for boosting) is not widely available. Pretreatment resistance due to exposure to maternal ART or neonatal prevention of vertical transmission might also affect treatment success.^4^, ^5^

**Research in context**

**Evidence before this study**

Several studies in adults living with HIV have showed that dolutegravir-based antiretroviral therapy (ART) is safe and effective at achieving higher rates of viral suppression, with lower incidence of emerging HIV drug resistance and fewer drug–drug interactions, than other ART options. These trials preceded the WHO recommendation of dolutegravir-based ART as the preferred ART in adults living with HIV. We searched PubMed for trials evaluating dolutegravir in children up to May 16, 2022 with the search terms: (dolutegravir[Title/Abstract] AND (children[Title/Abstract] OR pediatric[Title/Abstract] OR paediatric[Title/Abstract]). We identified four publications from the ODYSSEY trial, including the study design paper, the results of the main trial, and two papers describing pharmacokinetic and safety data in children weighing 4 kg to less than 20 kg, and 20 kg to less than 40 kg, respectively. We identified four publications from IMPAACT P1093, describing results of their dolutegravir dose-finding study in children aged 4 weeks to 6 years (n=1 publication) and in adolescents (n=2; aged 12 to <18 years), and resistance across cohorts (n=1). The main ODYSSEY trial, which primarily enrolled children older than 6 years (weighing ≥14 kg), showed superior efficacy of dolutegravir-based ART compared with non-dolutegravir-based standard-of-care treatment in children living with HIV starting first-line or second-line ART, with improved lipid profiles and reassuring results on minimal difference in weight gain between trial groups. The nested pharmacokinetic studies in both the main ODYSSEY trial and the below 14 kg cohort, alongside the IMPAACT P1093 dose-finding study, provided pharmacokinetic and non-randomised safety data on appropriate dolutegravir dosing for children aged 4 weeks and older. ODYSSEY showed that children weighing at least 20 kg can be given the adult dolutegravir dose (50 mg film coated tablets), and ODYSSEY and IMPAACT P1093 informed dosing of dispersible dolutegravir in children weighing from 3 kg upwards. This paper presents the first randomised trial data on the efficacy and safety of dolutegravir compared with standard-of-care ART in children weighing less than 14 kg.

**Added value of this study**

Results in children weighing less than 14 kg in ODYSSEY show that dolutegravir has superior efficacy compared with standard of care (mostly ritonavir-boosted lopinavir-based ART) and is safe in young children from age 4 weeks, weighing at least 3 kg. Analysis of the primary endpoint of treatment failure used a methodologically innovative and preplanned Bayesian analysis, borrowing information from older children in the main ODYSSEY trial, with the extent of borrowing defined by clinical opinion.

**Implications of all the available evidence**

This study, together with the ODYSSEY pharmacokinetic studies and the IMPAACT P1093 study, provides comprehensive data for regulators, policy makers, key donors, funders, and implementers who are enabling rapid adoption of dolutegravir-based ART as the preferred treatment for infants and children from 4 weeks of age. This evidence facilitates the alignment of treatment for young children with that for older children, adolescents, and adults. This study successfully employed an innovative Bayesian analysis of a small cohort which could be considered in other paediatric drug trials. Taking this approach could reduce the time from research to implementation, which is crucial in improving treatment outcomes for children across different disease areas.

Previous trials have shown that dolutegravir-based ART regimens are safe and effective at achieving high rates of virological suppression among adults living with HIV.^6^, ^7^, ^8^, ^9^, ^10^ Dolutegravir-based regimens are also more durable with a lower risk of developing major drug resistance mutations compared with non-nucleoside reverse transcriptase inhibitor (NNRTI)-based ART regimens.^8^, ^9^, ^11^, ^12^, ^13^ The ODYSSEY trial aimed to compare the efficacy and safety of dolutegravir-based ART versus standard of care in children starting first-line or second-line ART.^14^ The main trial recruited children weighing at least 14 kg and showed superiority of dolutegravir-based ART.^15^ Based on a nested pharmacokinetic and safety substudy,^16^ the US Food and Drug Administration (FDA) and European Medicines Agency approved the adult 50 mg dolutegravir dose in children weighing 20 kg or more. Worldwide, adults and children weighing at least 20 kg are rapidly switching to dolutegravir-based ART for first-line or second-line therapy.^17^

While a paediatric single-arm dolutegravir dose-finding study (IMPAACT P1093; NCT01302847) was ongoing,^18^ ODYSSEY randomly assigned a cohort of children aged 4 weeks and older and weighing between 3 kg and less than 14 kg (<14 kg cohort) in South Africa, Uganda, and Zimbabwe to evaluate the efficacy and safety of dolutegravir-based ART compared with standard of care. Children in the dolutegravir group were also enrolled in a pharmacokinetic substudy.^19^ Both pharmacokinetic studies provided data for regulatory submission of 5 mg paediatric dispersible dolutegravir formulation.^18^, ^19^ Here we report on the results of the ODYSSEY efficacy and safety study in children weighing less than 14 kg.

## Methods

### Study design

ODYSSEY is an open-label, randomised, parallel-group, non-inferiority trial comparing dolutegravir plus two nucleoside or nucleotide reverse transcriptase inhibitors (NRTIs; dolutegravir arm) versus standard of care (NNRTI, boosted protease inhibitor, or non-dolutegravir integrase inhibitor, plus two NRTIs) in infants, children, and adolescents with HIV (younger than 18 years), starting first-line ART (ODYSSEY-A) or switching to second-line ART after treatment failure with first-line regimens (ODYSSEY-B).^14^ The main trial recruitment period was between Sept 20, 2016 and June 22, 2018 and was open to children weighing at least 14 kg, with children weighing less than 14 kg ineligible for random assignment during this period due to absence of an appropriate dolutegravir formulation and dosing for weight bands lower than 14 kg. Recruitment of children weighing less than 14 kg opened on July 5, 2018 using a 5 mg dispersible tablet with dosing based on preliminary IMPAACT P1093 data. The target sample size for the main trial was achieved before enrolment of the less than 14 kg cohort. The less than 14 kg cohort was recruited from seven HIV treatment centres in South Africa, Uganda, and Zimbabwe (appendix p 101). Children were followed up until June 28, 2021, when the last child recruited reached 96 weeks, and all children remaining in follow-up were then seen at the end of the randomised phase (last visit Oct 31, 2021). National and local ethics committees and the ethics committee at University College London (London, UK) approved the trial. The ODYSSEY protocol is available online.

### Participants

Participants were infants and children living with HIV aged 4 weeks or older and weighing between 3 kg and less than 14 kg. Children starting first-line ART were enrolled in ODYSSEY-A. Children eligible for ODYSSEY-B were those starting second-line ART after failure of first-line regimens, with an HIV-1 RNA viral load of at least 500 copies per mL within 4 weeks of screening. Second-line ART was selected by the treating clinicians and should have included a new third drug and at least one NRTI likely to have preserved activity on the basis of treatment history. Main exclusion criteria for both ODYSSEY-A and ODYSSEY-B were severe hepatic impairment or unstable liver disease or previous integrase inhibitor exposure for more than 2 weeks. Full eligibility criteria are listed in the appendix (p 102). Carers gave written informed consent for the trial.

### Randomisation and masking

Randomisation was 1:1 to dolutegravir-based ART versus standard of care using permuted blocks and stratified by enrolment group (ODYSSEY-A or ODYSSEY-B) and weight band (3 kg to <6 kg; 6 kg to <10 kg; and 10 kg to <14 kg). A computer-generated randomisation list was prepared by a trial statistician (DF) and incorporated within a trial database, which was accessed via an in-house browser-based data entry system, and enabled access only to the next random allocation. Viral loads were analysed by laboratory staff masked to treatment arm, and all reported clinical events were adjudicated by an independent endpoint review committee, also masked to treatment. Only the trial statisticians (BW, EW, and DF) and the independent data monitoring committee saw aggregated data by randomised arm during the trial.

### Procedures

Participants were seen at screening, enrolment (week 0), weeks 4 and 12, and then every 12 weeks, when height, weight, HIV disease stage, adverse events, and treatment adherence were assessed. Adverse events were graded according to the Division of AIDS (DAIDS) Table for Grading the Severity of Adult and Pediatric Adverse Events (version 2.0). CD4, CD8, biochemistry, and haematology tests were performed at baseline (week 0; apart from biochemistry at screening), weeks 4 and 24, and then every 24 weeks; lipids and glucose were measured at baseline and every 48 weeks. Real-time viral load testing was done every 6–12 months as per local practice (40 of 79 participants in follow-up for ≥24 weeks had two or three real-time viral loads by 96 weeks; 34 of 79 had four or more). Retrospective viral load testing was completed on plasma stored at baseline, weeks 4 and 12, and then every 12 weeks for timepoints when real-time viral load testing was not done (results were not returned to treating clinicians). Dolutegravir was administered once a day in 5 mg dispersible tablets according to WHO weight bands (5 mg or 10 mg in weight band 3 kg to <6 kg [dose dependent on child age of <6 months or ≥6 months]; 15 mg in weight band 6 kg to <10 kg; 20 mg in weight band 10 kg to <14 kg; and 25 mg in weight band 14 kg to <20 kg).

### Outcomes

The primary endpoint was virological or clinical failure (ie, treatment failure) by 96 weeks, defined as first occurrence of any of: a less than 1 log_10_ drop in viral load at week 24 from baseline (or viral load ≥50 copies per mL at week 24 if viral load was <500 copies per mL at baseline) and a switch to second-line or third-line ART (at or after 24 weeks) due to treatment failure; two consecutive viral load results of at least 400 copies per mL, the first at or after week 36; a new or recurrent AIDS-defining event (WHO stage 4) or severe WHO stage 3 event;^20^ or all-cause death. Secondary efficacy endpoints were: virological or clinical failure by 48 weeks; time to new or recurrent WHO stage 4 or severe WHO stage 3 event; rates of WHO stage 4 and severe WHO stage 3 events and deaths over 96 weeks; proportions of children with cross-sectional viral load less than 50 copies per mL or less than 400 copies per mL at 48 and 96 weeks; change in CD4 count and percentage and CD4 to CD8 ratio from baseline to weeks 48 and 96; and proportion of children with major drug resistance mutations recognised by the International AIDS Society (IAS)–USA.^21^ Real-time and retrospective viral loads were used for measurement of outcomes. Participants meeting a virological component of the primary endpoint were retrospectively tested for post-failure drug resistance mutations up to week 96, with use of the most recent sample with a viral load of at least 1000 copies per mL after failure and before a treatment change. Change in total cholesterol from baseline to week 96 was the prespecified main secondary endpoint for assessing the safety of dolutegravir-based ART versus standard of care. Other secondary safety endpoints were incidence of serious adverse events, grade 3 or higher adverse events (DAIDS grading criteria), any grade adverse events leading to treatment modification, and change in triglycerides and lipid fractions from baseline to weeks 48 and 96. Carer-reported secondary endpoints were adherence and acceptability assessed by questionnaires at regular intervals (adherence assessed at screening [ODYSSEY-B participants only], 4 and 12 weeks, and then every 12 weeks; acceptability assessed at weeks 0, 4, 12, and 24, and then every 24 weeks). All clinical events under the remit of the primary and secondary outcomes were reviewed against prespecified criteria by the endpoint review committee. Anthropometric measures were not secondary outcomes in the protocol but were prespecified for analysis in the statistical analysis plan and are presented as they are of interest due to adult data suggesting excess weight gain on dolutegravir-based ART.^11^, ^13^, ^22^, ^23^

### Statistical analysis

The target sample size of 700 children was reached in the main trial (children weighing ≥14 kg); providing 90% power to demonstrate that dolutegravir-based ART was not inferior to standard of care, assuming a failure rate of 18% overall in both arms by 96 weeks, 10% loss to follow-up, and a 10% non-inferiority margin. Children weighing less than 14 kg were recruited in addition to the sample size for the main trial. The sample size for the lower weight cohort was based on enrolling at least eight children with evaluable pharmacokinetic curves on dolutegravir in each of the three weight bands (3 kg to <6 kg, 6 kg to <10 kg, and 10 kg to <14 kg). To achieve these numbers, at least 20 children were randomly assigned in each weight band (total ≥60 children). Power for efficacy and the numbers receiving first-line and second-line therapy were not prespecified for this cohort. To address the small sample size, the primary analysis of efficacy was prespecified as a Bayesian analysis, borrowing information from the main trial cohort.

Follow-up was censored on June 28, 2021, when the last participant reached 96 weeks, or, if earlier than June 28, at loss to follow-up, or at the last viral load assessment for virological endpoints. Comparisons between randomised arms were based on intention to treat (including all randomly assigned participants) adjusting for ODYSSEY-A and ODYSSEY-B strata. The primary outcome was assessed with adjusted Kaplan-Meier curves to estimate the proportion of children in whom treatment failed in each randomised arm by week 96. A cumulative failure function for each randomised arm was estimated as a weighted mean of the corresponding stratum-specific cumulative failure functions (estimated from a Cox model adjusting for ODYSSEY-A and ODYSSEY-B strata and randomised arm), with weights proportional to the number of participants in each stratum at baseline. CIs for the proportions of children with treatment failure by treatment arm were estimated by bootstrapping on the log{–log[S(t)]} scale, assuming normal theory, with the scale chosen to allow for non-symmetrical CIs on the probability scale. The SE for the difference in treatment failure at 96 weeks (dolutegravir minus standard of care) was estimated by bootstrapping on the risk difference scale. Bayesian estimation was then used for the primary analysis of the difference in treatment failure by 96 weeks by arm in participants weighing less than 14 kg. An informative prior distribution was used based on the treatment effect observed in participants weighing 14 kg or more, with relative weight defined by clinical opinion, solicited prior to the main trial results. We pooled treatment effects for the two cohorts using a weight of 78% for the children in the 14 kg and greater cohort (effectively down-weighting 707 children to 301) and 22% for 85 children in the below 14 kg cohort (appendix pp 8–9).^24^ In secondary analyses of the primary outcome, we did conventional frequentist analysis of the below 14 kg cohort, in which (in contrast to the Bayesian approach) only data from this cohort were used to draw conclusions, and of the whole trial population (<14 kg and ≥14 kg); in both analyses the CI for the risk difference was estimated by bias-corrected bootstrapping. A χ^2^ test was used to test for heterogeneity of treatment effect by weight cohort (<14 kg and ≥14 kg).

Secondary outcomes are reported only for the below 14 kg cohort, and analyses of secondary outcomes used a conventional frequentist intention-to-treat approach. The FDA snapshot algorithm, which incorporates information on treatment changes, deaths, and losses to follow-up,^25^ was used to compare the proportions with virological suppression (viral load <50 copies per mL and <400 copies per mL) at 48 and 96 weeks by treatment arm (appendix pp 10–11). Regimen change was defined for the overall trial as change of the third drug (not part of the NRTI component) for treatment failure, toxicity, or major protocol deviation (appendix p 12). In a per-protocol analysis of the below 14 kg cohort (frequentist approach), follow-up was censored at regimen change or ART discontinuation for longer than 31 days. Numbers of adverse events and numbers of participants experiencing at least one adverse event are presented; treatment arms were compared by time to first event using Cox regression. Changes in continuous outcomes (including immunological, metabolic, and anthropometric measures) were assessed with ANCOVA, adjusting for baseline value and enrolment group strata (ODYSSEY-A and ODYSSEY-B). Participants with viral loads of less than 50 copies per mL and less than 400 copies per mL at 48 and 96 weeks were compared between arms on the basis of cross-sectional proportions. Between-group differences were estimated by the marginal risk differences from logistic regression models. In an exploratory analysis, we calculated the cumulative probability of confirmed virological suppression (two consecutive viral loads <400 copies per mL) from cause-specific hazard functions and overall survival curves, treating a switch from initial trial regimen or a switch for treatment failure in NRTI backbone as a competing risk; death was also a competing risk. Hazard ratios (HRs) based on cumulative incidence (data not shown) were similar to the cause-specific HRs presented.

All p values are two-sided. All analyses were completed with Stata 16 (version 16.1). The ODYSSEY trial is registered with ClinicalTrials.gov, NCT02259127.

### Role of the funding source

ViiV Healthcare reviewed the study design and reviewed and provided comments on the manuscript. ViiV Healthcare did not participate in data collection, data analysis, or data interpretation. Employees of the UK Medical Research Council and Paediatric European Network for Treatment of AIDS Foundation were authors of the paper and were involved in study design, data collection, data analysis, data interpretation, and writing of the report.

## Results

**Figure 1:**
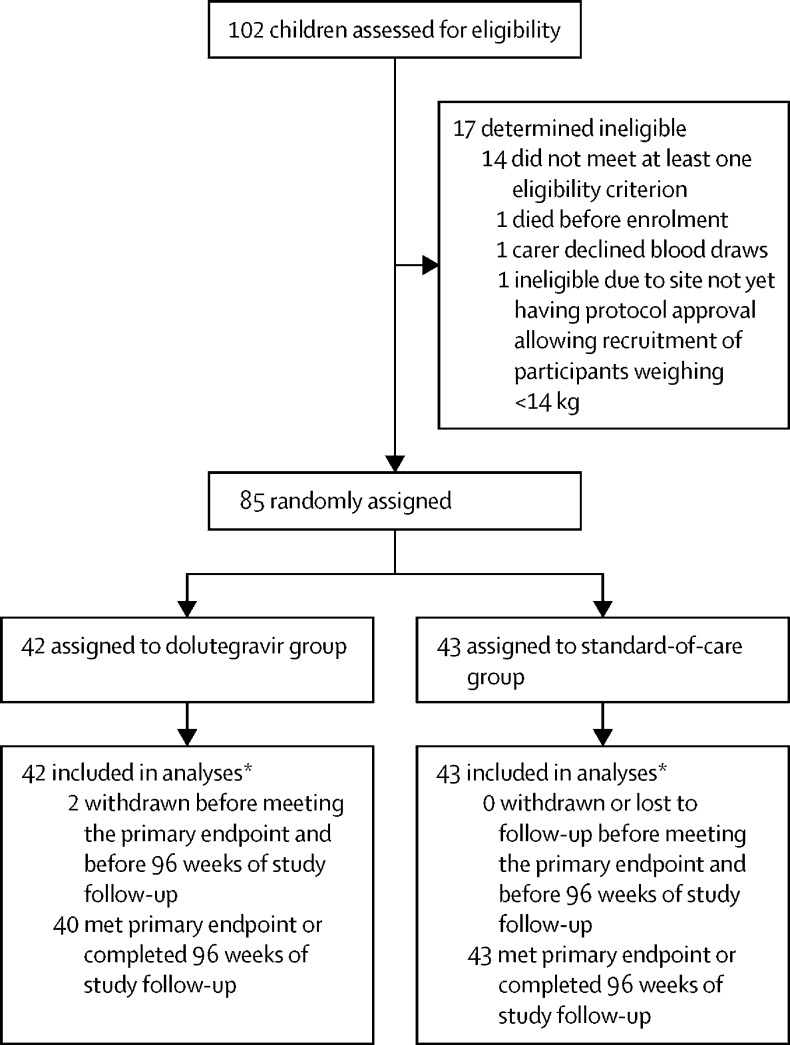
Trial profile *All enrolled participants contributed to the primary endpoint (appendix pp 8–9).

Between July 5, 2018, and Aug 26, 2019, 102 children were assessed for eligibility and 85 were enrolled (Uganda n=43; Zimbabwe n=22; South Africa n=20). 42 were randomly assigned to dolutegravir-based ART and 43 to standard of care (figure 1). Baseline characteristics were similar in both groups (table 1). Median age was 1·4 years (IQR 0·6–2·0) with 76 (89%) of 85 children younger than 3 years. Median weight was 8·1 kg (5·4–10·0); 23 (27%) participants were in the 3 kg to less than 6 kg weight band, 40 (47%) were in the 6 kg to less than 10 kg weight band, and 22 (26%) were in the 10 kg to less than 14 kg weight band. Among 81 participants with available data, 18 (22%) had a CD4 percentage of less than 15% at enrolment. Enrolment viral loads were high; 27 participants (34%; n=80 with available data) had a viral load between 100 000 and less than 500 000 copies per mL, and 24 (30%) had a viral load of 500 000 copies per mL or higher. Exposure to ART to prevent vertical transmission was reported in around half the children (38 [48%] of 80), and, when known, was predominantly nevirapine (28 [80%] of 35). 27 (32%) of the 85 children had a weight-for-age z score lower than –3. Overall, 72 (85%) children started first-line ART (ODYSSEY A cohort). In the standard-of-care group, 29 (78%) of 37 children started ritonavir-boosted lopinavir-based ART, four (11%) started efavirenz-based ART, and four (11%) started nevirapine-based ART (0 of 8 who started on an NNRTI had previous nevirapine exposure). 13 (15%) children started second-line ART (ODYSSEY B cohort). In the standard-of-care group, three (50%) of six started ritonavir-boosted lopinavir-based ART, two (33%) started raltegravir-based ART, and one (17%) started nevirapine-based ART. NRTI backbones were balanced across groups; 75 children (38 in the dolutegravir group and 37 in the standard-of-care group) received abacavir–lamivudine and ten children (four in the dolutegravir group and six in the standard-of-care group) received zidovudine–lamivudine (appendix p 79).

**Table 1:** Baseline characteristics

		**Dolutegravir**	**Standard of care**	**Total**
Participants		42	43	85
Country				
	South Africa	8 (19%)	12 (28%)	20 (24%)
	Uganda	22 (52%)	21 (49%)	43 (51%)
	Zimbabwe	12 (29%)	10 (23%)	22 (26%)
ODYSSEY A or ODYSSEY B				
	ODYSSEY A (starting first-line ART)	35 (83%)	37 (86%)	72 (85%)
	ODYSSEY B (switching to second-line ART)	7 (17%)	6 (14%)	13 (15%)
Sex				
	Female	26 (62%)	18 (42%)	44 (52%)
	Male	16 (38%)	25 (58%)	41 (48%)
Age at enrolment, years				
	Median	1·3 (0·5 to 2·0; 0·3 to 5·9)	1·5 (0·6 to 2·1; 0·1 to 4·5)	1·4 (0·6 to 2·0; 0·1 to 5·9)
	<6 months	11 (26%)	8 (19%)	19 (22%)
	6 months to <1 year	5 (12%)	8 (19%)	13 (15%)
	1 year to <3 years	22 (52%)	22 (51%)	44 (52%)
	3 years to <6 years	4 (10%)	5 (12%)	9 (11%)
Weight, kg				
	Median	8·1 (5·6 to 10·0; 3·8 to 13·0)	8·2 (5·2 to 10·3; 3·4 to 13·4)	8·1 (5·4 to 10·0; 3·4 to 13·4)
	3 kg to <6 kg	11 (26%)	12 (28%)	23 (27%)
	6 kg to <10 kg	20 (48%)	20 (47%)	40 (47%)
	10 kg to <14 kg	11 (26%)	11 (26%)	22 (26%)
Weight-for-age z score				
	Median	−2·1 (−3·4 to −1·3; −5·1 to 0·3)	−1·8 (−3·7 to −0·8; −5·7 to 1·4)	−1·9 (−3·4 to −1·1; −5·7 to 1·4)
	<–3	14 (33%)	13 (30%)	27 (32%)
	−3 to <–2	7 (17%)	6 (14%)	13 (15%)
	−2 to <0	18 (43%)	21 (49%)	39 (46%)
	≥0	3 (7%)	3 (7%)	6 (7%)
BMI-for-age z score				
	Median	−1·1 (−1·9 to 0·2; −3·6 to 1·9)	−0·7 (−2·2 to 0·3; −5·7 to 3·1)	−0·8 (−2·0 to 0·2; −5·7 to 3·1)
	<–3	3 (7%)	6 (14%)	9 (11%)
	−3 to <–2	6 (14%)	6 (14%)	12 (14%)
	−2 to <0	20 (48%)	14 (33%)	34 (40%)
	≥0	13 (31%)	17 (40%)	30 (35%)
CD4 percentage*				
	Median	24 (17 to 35)	23 (14 to 30)	23 (16 to 31)
	<15%	7 (17%)	11 (28%)	18 (22%)
	15% to <30%	22 (54%)	18 (45%)	40 (49%)
	≥30%	12 (29%)	11 (28%)	23 (28%)
	Missing	1	3	4
CD4, cells per mm^3^*				
	Median	1639 (1026 to 2327)	1221 (633 to 1870)	1391 (863 to 2060)
Viral load, copies per mL*				
	<10 000	4 (10%)	7 (18%)	11 (14%)
	10 000 to <100 000	13 (31%)	5 (13%)	18 (23%)
	100 000 to <500 000	12 (29%)	15 (39%)	27 (34%)
	≥500 000	13 (31%)	11 (29%)	24 (30%)
	Missing†	0	5	5
Log_10_ viral load, copies per mL*				
	Median	5·2 (4·4 to 5·8)	5·4 (4·8 to 5·9)	5·3 (4·6 to 5·9)
History of WHO HIV/AIDS staging‡				
	Stage 1–2	31 (74%)	25 (58%)	56 (66%)
	Stage 3	6 (14%)	8 (19%)	14 (16%)
	Stage 4	5 (12%)	10 (23%)	15 (18%)
Prevention of vertical transmission ART exposure				
	No	19 (46%)	23 (59%)	42 (53%)
	Yes	22 (54%)	16 (41%)	38 (48%)
	Unknown	1	4	5

Data are n (%), median (IQR; range), or median (IQR). Percentages are for the non-missing proportion. ART=antiretroviral therapy.

* Mean of measurement at screening and randomisation if both were available.

† In each of the five participants with missing baseline viral load measurements, the sites were unable to draw a sufficient volume of blood for testing.

‡ Worst known stage prior to enrolment.

**Figure 2:**
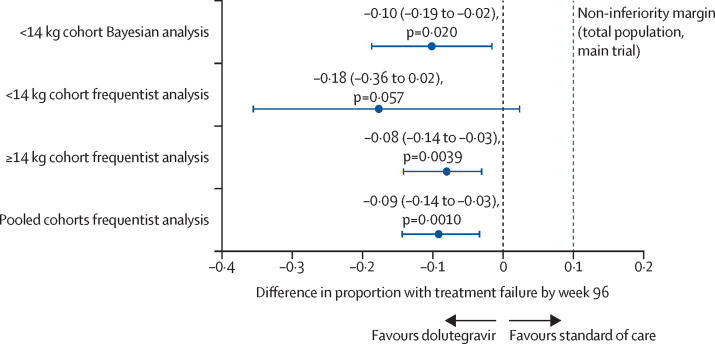
Difference in proportion of participants with virological or clinical failure by 96 weeks

Median follow-up was 124 weeks (112–137). 83 (98%) children were seen at or after 96 weeks or reached the primary endpoint. By 96 weeks, treatment failure occurred in 12 children in the dolutegravir group (Kaplan-Meier estimated proportion 31% [95% CI 18–48]) versus 21 (48% [35–63]) in the standard-of-care group (appendix p 23, table 2). Nine participants in the dolutegravir group versus 16 in the standard-of-care group met the primary endpoint for virological failure, although a significant proportion were not virally suppressed (two consecutive viral loads <400 copies per mL) prior to virological failure (three [33%] of nine receiving dolutegravir; 13 [81%] of 16 receiving standard of care). The Bayesian estimated difference in treatment failure (dolutegravir minus standard of care) in participants weighing less than 14 kg was –10% (95% CI –19 to –2; p=0·020), demonstrating superiority of dolutegravir-based ART. Frequentist analysis of the lower weight cohort (<14 kg) alone provided an estimated difference in treatment failure of –18% (–36 to 2; p=0·057). In the pooled cohorts (<14 kg and ≥14 kg), estimated difference was –9% (–14 to –3; p=0·0010; figure 2). A per-protocol analysis of the lower weight (<14 kg) cohort alone gave a similar estimated difference in treatment failure (–17% [–36 to 3; p=0·075]) to the corresponding intention-to-treat analysis. We found no evidence for different treatment effects by cohort (<14 kg *vs* ≥14 kg cohorts; heterogeneity p=0·32). Among children weighing less than 14 kg, treatment effects were consistent with a benefit of dolutegravir over standard of care in children starting first-line or second-line therapy (heterogeneity p=0·81). By 48 weeks, there was already some evidence for a benefit in the dolutegravir group (estimated difference in treatment failure of –16% (–35 to 0; p=0·063; appendix p 19). In an exploratory analysis, we showed suppression happened earlier in the dolutegravir group than in the standard-of-care group (cause-specific HR 2·27 [95% CI 1·37–3·79]; p=0·0016; appendix p 34). Per the FDA snapshot algorithm, 27 (64%) of 42 participants in the dolutegravir group versus 18 (42%) of 43 in the standard-of-care group had an HIV-1 RNA viral load less than 50 copies per mL at 96 weeks (adjusted p=0·035); corresponding proportions for a viral load lower than 400 copies per mL were 32 (76%) participants versus 22 (51%) participants (adjusted p=0·018; appendix pp 27–30).

**Table 2:** Efficacy endpoints comparing dolutegravir-based ART with standard of care*

			**Dolutegravir**	**Standard of care**	**Dolutegravir versus standard of care**
Participants			42	43	..
Primary endpoint: virological failure or clinical failure by 96 weeks					
	Participants with virological or clinical failure by 96 weeks		12 (29%)	21 (49%)	..
Primary endpoint components					
	Insufficient virological response at 24 weeks		0	0	..
	Confirmed viral load ≥400 copies per mL at >36 weeks		9 (21%)	16 (37%)	..
	Severe WHO 3 stage event		0	0	..
	WHO 4 stage event		1 (2%)	1 (2%)	..
	Death		2 (5%)	4 (9%)	..
Estimated probability of virological or clinical failure by 96 weeks (95% CI)					
	Frequentist analysis		0·31 (0·18 to 0·48)	0·48 (0·35 to 0·63)	−0·18 (−0·36 to 0·02), p=0·057†
	Bayesian analysis		NA	NA	−0·10 (−0·19 to −0·02), p=0·020
Secondary endpoint: cross-sectional viral load suppression at 48 weeks‡					
	Participants with viral load <50 copies per mL at 48 weeks		15/34	19/39	..
		Proportion (95% CI)	44% (28 to 61)	49% (33 to 65)	−4% (−26 to 19)
		p value	..	..	p=0·76
	Participants with viral load <400 copies per mL at 48 weeks		25/34	27/39	..
		Proportion (95% CI)	74% (56 to 86)	69% (53 to 82)	5% (−16 to 26)
		p value	..	..	p=0·64
Secondary endpoint: cross-sectional viral load suppression at 96 weeks‡					
	Participants with viral load <50 copies per mL at 96 weeks		27/35	19/36	..
		Proportion (95% CI)	77% (60 to 88)	53% (36 to 69)	26% (6 to 47)
		p value	..	..	p=0·021
	Participants with viral load <400 copies per mL at 96 weeks		33/36	26/36	..
		Proportion (95% CI)	92% (76 to 97)	72% (55 to 85)	19% (2 to 37)
		p value	..	..	p=0·038
Secondary endpoint: CD4 cell count, cells per μL§					
	Mean change (SE) in CD4 cell count from baseline to 96 weeks		72 (116)	51 (118)	30 (95% CI −308 to 368)
	p value		..	..	p=0·86
Secondary endpoint: CD4 percentage§					
	Mean change (SE) in CD4 percentage from baseline to 96 weeks		13% (2)	8% (2)	5% (95% CI 0 to 9)
	p value		..	..	p=0·053

NA=not available.

* Comparisons of treatment groups are presented for the dolutegravir group as compared with the standard of care group; the probability of having virological or clinical treatment failure by 96 weeks (primary endpoint) was estimated using Kaplan-Meier curves adjusted for trial cohort (ODYSSEY A or ODYSSEY B; appendix p 9); proportions of participants who had other endpoint events at or by 48 or 96 weeks were unadjusted.

† p=0·81 for the interaction between trial group (dolutegravir or standard of care) and trial cohort (ODYSSEY A or ODYSSEY B) for the primary endpoint.

‡ The between-group differences in the percentages of participants with a viral load of less than 50 copies per mL and of less than 400 copies per mL at 48 and 96 weeks are the marginal risk differences from the respective logistic regression models and are presented in percentage points.

§ Mean changes in CD4 count and CD4 percentage from baseline to 96 weeks were calculated with the use of normal regression with adjustment for baseline measure; estimates are presented for mean change from a baseline CD4 count of 1550 cells per μL and a mean change from a baseline CD4 percentage of 24%; the between-group difference in the mean change from baseline was calculated with the use of normal regression with adjustment for baseline measure and enrolment in ODYSSEY A or ODYSSEY B.

At week 48, cross-sectional proportions of children with a viral load less than 50 copies per mL and less than 400 copies per mL (ignoring treatment changes and excluding losses to follow-up and deaths before week 48) were similar in both groups. At week 96, significantly more children had virological suppression in the dolutegravir group using either threshold; among the children with available data, 33 of 36 (92% [95% CI 76 to 97]) in the dolutegravir group versus 26 of 36 (72% [55 to 85]) in the standard-of-care group had a viral load less than 400 copies per mL (table 2).

Up to the trial censoring date, there were three new or recurrent severe WHO stage 3 or stage 4 events or deaths (three participants) in the dolutegravir group, versus six events or deaths (six participants) in the standard-of-care group, with no evidence of a difference between groups (appendix pp 35–36). At 96 weeks, we observed a modest difference in CD4 percentage gain from baseline favouring dolutegravir, with a mean difference for dolutegravir versus standard of care of 5% (95% CI 0–9; p=0·053; table 2). CD4 count and CD4 to CD8 ratio did not differ significantly by trial group (table 2; appendix pp 48–50).

All participants with virological failure by week 96 had at least one major IAS–USA drug resistance mutation identified after treatment failure. Of those with virological failure on first-line therapy, one (17%) of six receiving dolutegravir versus ten (83%) of 12 receiving standard of care had NRTI-resistant mutations, six (100%) versus 11 (92%) had NNRTI-resistant mutations, and zero versus two (17%) had protease inhibitor-resistant mutations (appendix p 38). Of five participants with virological failure on first-line dolutegravir (data unavailable for the remaining participant), none had major integrase strand transfer inhibitor (INSTI) resistance. For those on second-line therapy, one (50%) of two participants receiving dolutegravir versus all three (100%) receiving standard of care had at least one NRTI mutation identified after treatment failure, all had NNRTI-resistant mutations, and none had protease inhibitor mutations. One (50%) of the two participants with virological failure on second-line dolutegravir had an Asn155His INSTI mutation (receiving dolutegravir with zidovudine–lamivudine).

Up to the trial censoring date, similar proportions of children in each group had experienced one or more serious adverse events (15 events reported in 11 [26%] of 42 participants in the dolutegravir group *vs* 19 events in 11 [26%] of 43 participants in the standard-of-care group; HR 1·08 [95% CI 0·47–2·49]; p=0·86; table 3). 26 (76%) of 34 serious adverse events were hospitalisations, commonly due to infections (appendix pp 66–67). Six participants died (two in the dolutegravir group and four in the standard-of-care group). Three of six deaths were within one week of enrolment, and two of the three children who died after the first week had WHO stage 4 disease at enrolment (appendix p 37). Similar proportions of children had one or more adverse events of grade 3 or worse (36 events in 19 [45%] participants in the dolutegravir group *vs* 34 events in 21 [49%] participants in the standard-of-care group; HR=0·93 [0·50–1·74]; p=0·83; table 3). Two ART-modifying adverse events (any grade) were reported, both in the standard-of-care group, although one child returned to the same first-line ART regimen after a treatment interruption (table 3). No adverse events were considered related to dolutegravir by investigators or the endpoint review committee. One participant in each group had an immune reconstitution inflammatory syndrome event (tuberculosis in the dolutegravir group and thrombocytopenia in the standard-of-care group). Both events resolved during the trial.

**Table 3:** Safety endpoints comparing dolutegravir-based ART with standard of care*

		**Dolutegravir**	**Standard of care**	**Dolutegravir versus standard of care**
Participants		42	43	..
Serious adverse events†				
	Number of events	15	19	..
	Number of participants	11	11	HR 1·08 (0·47 to 2·49), p=0·86
Grade ≥3 adverse events†				
	Number of events	36	34	..
	Number of participants	19	21	HR 0·93 (0·50 to 1·74), p=0·83
ART-modifying events (any grade)†				
	Number of events	0	2‡	..
	Number of participants	0	2	NE
Metabolic outcomes				
	Mean change (SE) in total cholesterol, mg/dL, from baseline to 96 weeks†§	4·5 (5·6)	29·6 (5·9)	−24·4 (−40·3 to −8·5), p=0·0032
Anthropometric measures				
	Mean change (SE) in weight, kg, from baseline to 96 weeks§¶	5·0 (0·2)	5·1 (0·2)	−0·1 (−0·8 to 0·5), p=0·67
	Mean change (SE) in BMI-for-age z score from baseline to 96 weeks§¶	1·1 (0·3)	1·5 (0·3)	−0·3 (−1·1 to 0·5), p=0·50
	Mean change (SE) in weight-for-age z score from baseline to 96 weeks§¶	1·2 (0·2)	1·2 (0·2)	0·00 (−0·5 to 0·5), p=1·00

ART=antiretroviral therapy. NE=not estimable.

* Comparisons of treatment groups are presented for the dolutegravir group as compared with the standard of care group.

† This secondary safety endpoint was specified in the protocol; additional secondary endpoints are presented in the appendix (pp 54–62); adverse events were compared between treatment groups using HRs for time to first event, adjusting for trial cohort (ODYSSEY A or ODYSSEY B).

‡ One event was raised liver enzymes, which was considered ART-modifying as the event led to the participant stopping their first-line ART regimen for 14 weeks, after which the participant restarted the same first-line ART regimen; the other event was vomiting, which led to substitution of the third agent.

§ Mean changes in continuous measures from baseline to 96 weeks were calculated with the use of normal regression with adjustment for baseline measure; estimates are presented for mean change in total cholesterol from a baseline value of 127·9 mg/dL; for mean change in weight from a baseline weight of 8·1 kg; for mean change in BMI-for-age z score from a baseline z score of −0·9; and for mean change in weight-for-age z score from a baseline z score of −2·2; between-group differences in mean changes were calculated with the use of normal regression with adjustment for baseline measure and enrolment in ODYSSEY A or ODYSSEY B.

¶ This endpoint was not a prespecified secondary endpoint in the protocol, but was included in a planned analysis in the statistical analysis plan.

At the trial censoring date or loss to follow-up, 41 (98%) participants in the dolutegravir group versus 39 (91%) in the standard-of-care group remained on their initial trial regimen (appendix p 80). One participant in the dolutegravir group stopped ART after not attending clinic and subsequently withdrew from the trial. Three participants in the standard-of-care group switched regimen due to treatment failure, and one due to toxicity; an additional child changed from abacavir to zidovudine due to treatment failure but remained on ritonavir-boosted lopinavir (appendix p 81).

Total cholesterol was lower in the dolutegravir group than in the standard-of-care group. At 96 weeks, the estimated difference in mean change from baseline (dolutegravir minus standard of care) was –24·4 mg/dL (95% CI –40·3 to –8·5; p=0·0032; table 3, appendix pp 51–52); differences were mainly in low-density lipoprotein (appendix pp 54–55). We found no differences between groups in changes from baseline in anthropometric measures (table 3, appendix pp 82–96). Among 27 children with a weight-for-agez score lower than –3 at enrolment there were six deaths before week 96 (two in the dolutegravir group and four in the standard-of-care group), two withdrawals before week 96 (one in each group), and two with missing week 96 data on weight-for-age z score (one in each group); among the remaining 17 participants, seven (70%) of ten receiving dolutegravir and four (57%) of seven receiving standard of care had a weight-for-age z score of –2 or higher by week 96 (appendix p 97). Carer-reported adherence was high and similar between groups (42 [10%] of 404 reports indicated a missed dose in the previous week in the dolutegravir group *vs* 53 (13%) of 405 in the standard-of-care group; p=0·50; appendix p 77). Acceptability was also high and similar between the groups (appendix p 78).

## Discussion

Randomised trial data from the below 14 kg ODYSSEY trial cohort provides evidence of superior efficacy of dolutegravir-based ART compared with standard of care in infants and young children living with HIV from 4 weeks of age, weighing 3 kg to less than 14 kg. Findings are consistent with results from the main ODYSSEY trial of older children living with HIV weighing at least 14 kg (median age 12 years).^15^ Of note, most children (n=72, 85%) in this young cohort were starting first-line ART, and 29 (78%) of 37 children receiving first-line ART in the standard-of-care group started ritonavir-boosted lopinavir-based ART.

The risk of treatment failure was significantly lower in the dolutegravir group than in the standard-of-care group. The overall treatment failure across both groups (33 [39%] of 85 children) was approximately 2-times higher than in older children in the main trial.^15^ Baseline viral loads were notably high (30% with viral load ≥500 000 copies per mL) in the young children. Most children (25 [76%] of 33) who met the primary endpoint did so based on confirmed viral load of at least 400 copies per mL after 36 weeks, and most had not achieved virological suppression (<400 copies per mL) before reaching the primary endpoint, consistent with previous studies that have shown longer time to virological suppression in young children.^26^ In an exploratory analysis of time to suppression, dolutegravir performed significantly better than standard of care; and in a cross-sectional analysis at 96 weeks, 92% (95% CI 76–97) of children receiving dolutegravir versus 72% (55–85) receiving standard of care had a viral load less than 400 copies per mL. Virological suppression in the dolutegravir group at 96 weeks according to the FDA snapshot algorithm (which incorporates information on treatment changes, deaths, and losses to follow-up) was similar (32 [76%] of 42 children with viral load <400 copies per mL) to that in older children in the dolutegravir group in the main ODYSSEY trial (82%)^15^ and adults receiving dolutegravir (around 80–90% on first-line ART at 96 weeks and around 75% on second-line ART at 48 weeks).^6^, ^7^, ^8^, ^9^, ^10^, ^11^, ^13^

A lower proportion of participants in the dolutegravir group had NRTI-resistant mutations after treatment failure than in the standard of care group; this finding is likely to be due to the combination of faster suppression and a lower rate of viral rebound with dolutegravir. One child in the below 14 kg cohort who experienced virological failure on second-line dolutegravir-based ART had potential low-level resistance to dolutegravir at treatment failure, with resistance also to zidovudine and lamivudine; the child achieved viral suppression again after failure without a change in ART. Adult and paediatric trials have also shown small numbers of participants, but numerically more on second-line ART than first-line ART, developing resistance to dolutegravir.^6^, ^10^, ^15^, ^27^ In both the adult NADIA trial and the main ODYSSEY trial, three of four participants on second-line ART who developed INSTI-resistant mutations were receiving zidovudine, which might be more challenging for adherence because it is given twice a day.^10^, ^15^

As in adults and older children,^12^, ^15^, ^28^ dolutegravir presented minimal safety concerns. Similar numbers of children experienced adverse events in the dolutegravir and standard-of-care groups, with no adverse events considered related to dolutegravir. Total cholesterol at 96 weeks was significantly lower in children receiving dolutegravir than in those receiving standard of care due to an increase in cholesterol in the standard-of-care group, probably driven by lipid changes associated with ritonavir-boosted lopinavir-based ART in most children receiving standard of care. Switching children from protease inhibitors to dolutegravir has the potential to reduce long-term risks of cardiovascular disease and might be important when children remain on lifelong ART. We observed no excess weight gain in the dolutegravir group, with children in both groups showing healthy weight gain, which is of importance given that 32% presented with very low weight-for-age (z score <–3). Weight gain results are in keeping with the minimal differences between groups observed in the main ODYSSEY trial, but in contrast to data in adults, in whom excess weight gain has been reported on dolutegravir.^11^, ^13^, ^22^, ^23^

Adherence and acceptability were similar between the dolutegravir and standard-of-care groups, although questionnaires might have limited capacity to assess these parameters accurately and trial participants are likely to be more adherent than children in routine care. We observed no treatment changes for treatment failure or toxicity in the dolutegravir group, although as the trial was open-label, clinicians might have been less willing to switch children off dolutegravir than other ART drugs.

The small number of children weighing less than 14 kg included in this analysis was recognised as a limitation for evaluating efficacy, and was addressed by a preplanned primary Bayesian analysis, incorporating an informative prior distribution on the basis of data from the larger main ODYSSEY trial.^24^ Similar Bayesian methods for analysing a small sample with borrowing of information from a relevant larger sample have been proposed previously in the statistical methods literature.^29^ The Bayesian approach allowed us to estimate the treatment effect with increased precision and confirmed the superior efficacy of dolutegravir, although it did rely on expert clinical opinion in terms of similarity of treatment effects in the two weight cohorts, which might not be reliable. To provide transparency, we report the Bayesian analysis alongside results from a stand-alone frequentist analysis and a pooled frequentist analysis. Of note, despite the small sample size, the stand-alone analysis of the 85 children in the below 14 kg cohort demonstrated non-inferiority of dolutegravir to standard care.

Countries around the world, including those in sub-Saharan Africa, have rapidly adopted dolutegravir-based regimens as first-line and second-line therapies for adults and older children (≥20 kg), following pharmacokinetic and efficacy results from the main ODYSSEY trial that showed children weighing at least 20 kg could safely take the once-daily adult formulation,^16^, ^15^ and subsequent recommendations by WHO.^30^ Pharmacokinetic and safety data from nested substudies in ODYSSEY^19^ and from IMPAACT P1093^18^ were submitted by ViiV Healthcare (with collaborative support from the Penta and IMPAACT networks) for regulatory approvals of dispersible dolutegravir for young children (age ≥4 weeks). Cooperation between generic drug manufacturers, the Clinton Health Access Initiative, WHO, Unitaid, and pharmaceutical industry has resulted in rapid evaluation, approval by the FDA, and availability of the 10 mg scored dispersible dolutegravir tablet for young children from age 4 weeks.^31^, ^32^

Before the comparative results from this below 14 kg cohort, there were no data to inform paediatric programmes or guideline committees about the efficacy of dolutegravir versus ritonavir-boosted lopinavir in the youngest children. The favourable efficacy results in the youngest children shown here should now ensure rapid and equitable distribution and access to dolutegravir-based dispersible once-daily ART regimens for infants and young children. This would represent a considerably shorter implementation time for children than the usual lag period in access to the most appropriate treatments after the treatments become available for adults, particularly in low-income and middle-income countries. Availability of dolutegravir across all ages in children and in adults would also allow harmonisation of paediatric and adult HIV treatment programmes.

In conclusion, the increased potency, high resistance barrier, safety and tolerability, acceptability, low cost, and once-daily dosing of dolutegravir compared with standard-of-care-based regimens presents an important milestone in providing improved treatment for infants and young children facing a lifetime of ART. These data support expedited roll-out of dolutegravir-based ART to all infants and children. Use of dolutegravir-based ART would also reduce inequity of ART access for vulnerable children and young people and increase momentum to identify all children living with HIV as early as possible to achieve the UNAIDS 95-95-95 goal for all age groups by 2030.^33^

## Data sharing

The ODYSSEY data are held at the Medical Research Council (MRC) Clinical Trials Unit at University College London, UK, which encourages optimal use of data by using a controlled access approach to data sharing, incorporating a transparent and robust system to review requests and provide secure data access consistent with the relevant ethics committee approvals. All requests for data are considered and can be initiated by contacting mrcctu.ctuenquiries@ucl.ac.uk.

## Declaration of interests

AT has received funding for her service on the Global Paediatrics Advisory Board between Oct 4, 2021, and Nov 14, 2021, with payments made to the MRC Clinical Trials Unit at University College London. All other authors declare no competing interests.
