# Supplementary material for: Once-daily dolutegravir-based antiretroviral therapy in infants and children living with HIV from age 4 weeks: results from the below 14 kg cohort in the randomised ODYSSEY trial
Source: Lancet HIV. 2022 Aug 30;9(9):e638–48. doi: 10.1016/S2352-3018(22)00163-1 (PMC9646993; doi:10.1016/S2352-3018(22)00163-1)
Supplement: Supplementary appendix [file mmc1.pdf]

THE LANCET
HIV

Supplementary appendix

This appendix formed part of the original submission and has been peer reviewed. We post it as supplied by the authors.

Supplement to: Amuge P, Lugemwa A, Wynne B, et al. Once-daily dolutegravir-based antiretroviral therapy in infants and children living with HIV from age 4 weeks: results from the below 14 kg cohort in the randomised ODYSSEY trial. *Lancet HIV* 2022; **9:** e638–48.

Supplementary Appendix

This appendix has been provided by the authors to give readers additional information about their work.

Contents

S1 ODYSSEY Trial Team 5
S2 Primary and secondary outcome measures in ODYSSEY 7
S3 Statistical methods 8
S3.1 Analysis populations 8
S3.2 Primary endpoint 8
S3.3 FDA snapshot algorithm 10
S3.4 Flowchart of the FDA snapshot algorithm 11
S3.5 Analysis of Continuous outcomes 12
S3.6 Analyses of clinical events 12
S3.7 Definition of treatment change 12
S3.8 Adjustment and testing multiple outcomes 12
S4 Impact of Covid-19 12
S5 Dolutegravir dosing throughout ODYSSEY 13
S6 Enrolment and Eligibility 14
S6.1 Screening and randomisation 14
S7 Baseline Demographics 15
S7.1 Baseline characteristics 15
S7.2 Baseline characteristics: Antiretroviral exposure [ODYSSEY B Only] 18
S8 Efficacy 19
S8.1 Comparison of proportion with clinical or virological failure by 48 weeks 19
S8.2 Incidence of clinical or virological failure by 48 weeks 20
S8.3 Comparison of proportion with clinical or virological failure by 96 weeks 21
S8.4 Incidence of clinical or virological failure by 96 weeks (alternative approach to analysis of primary endpoint) 22
S8.5 Time to clinical or virological failure 23
S8.6 Comparison of proportion with clinical or virological failure by 96 weeks 24
S8.7 Comparison of proportion of participants with HIV-1 RNA <50c/ml at 48 weeks 25
S8.8 Comparison of proportion of participants with HIV-1 RNA <400c/ml at 48 weeks 25
S8.9 Comparison of proportion of participants with HIV-1 RNA <50c/ml at 96 weeks 26
S8.10 Comparison of proportion of participants with HIV-1 RNA <400 c/ml at 96 weeks 26
S8.11 FDA snapshot algorithm - comparison of proportion of participants with HIV-1 RNA <50c/ml at 48 weeks 27
S8.12 FDA snapshot algorithm - comparison of proportion of participants with HIV-1 RNA <400 c/ml at 48 weeks 28
S8.13 FDA snapshot algorithm - comparison of proportion of participants with HIV-1 RNA <50 c/ml at 96 weeks 29
S8.14 FDA snapshot algorithm - comparison of proportion of participants with HIV-1 RNA <400 c/ml at 96 weeks 30
S8.15 Per-protocol analysis 31
S8.16 Total population - Subgroup analyses for primary endpoint 32
S8.17 ODYSSEY A - Subgroup analyses for primary endpoint 32
S8.18 Cumulative probability of suppression by treatment group (suppression defined as two consecutive viral loads <200cp/ml) 33
S8.19 Cumulative probability of suppression by treatment group (suppression defined as two consecutive viral loads <400cp/ml) 34
S8.20 Rate of clinical events to trial censoring date: WHO 4, severe WHO 3 and death 35
S8.21 Rate of clinical events to week 96: WHO 4, severe WHO 3 and death 36

S8.22 Details of WHO 3 (Severe) or 4 events or deaths during follow-up 37
S8.23 Genotypic resistance comparing dolutegravir-based ART versus standard of care# 38
S8.24 Resistance mutations identified post-failure in participants* 39
S8.25 Treatment emergent resistance mutations post-failure in participants exposed to drug-class* 41
S9 Immunology 42
S9.1 Total population - Changes in CD4 count over follow-up 42
S9.2 ODYSSEY A - changes in CD4 count over follow-up 42
S9.3 Total population - Changes in CD4 count over follow-up 43
S9.4 ODYSSEY A - changes in CD4 count over follow-up 44
S9.5 Total population - Changes in CD4 percentage over follow-up 45
S9.6 ODYSSEY A - changes in CD4 percentage over follow-up 45
S9.7 Total population - Changes in CD4 percentage over follow-up 46
S9.8 ODYSSEY A - changes in CD4 percentage over follow-up 47
S9.9 Total population - Changes in CD4/CD8 ratio over follow-up 48
S9.10 ODYSSEY A - changes in CD4/CD8 ratio over follow-up 48
S9.11 Total population - Changes in CD4/CD8 ratio over follow-up 49
S9.12 ODYSSEY A - changes in CD4/CD8 ratio over follow-up 50
S10 Lipids 51
S10.1 Total population - Total cholesterol changes over follow-up 51
S10.2 ODYSSEY A - Total cholesterol changes over follow-up 51
S10.3 Total population - changes in Total cholesterol over follow-up 52
S10.4 ODYSSEY A - changes in Total cholesterol over follow-up 53
S10.5 Total population - LDL cholesterol changes over follow-up 54
S10.6 ODYSSEY A - LDL cholesterol changes over follow-up 54
S10.7 Total population - changes in LDL cholesterol over follow-up 55
S10.8 ODYSSEY A - changes in LDL cholesterol over follow-up 56
S10.9 Total population - HDL cholesterol changes over follow-up 57
S10.10 ODYSSEY A - HDL cholesterol changes over follow-up 57
S10.11 Total population - changes in HDL cholesterol over follow-up 58
S10.12 ODYSSEY A - changes in HDL cholesterol over follow-up 59
S10.13 Total population - Triglycerides changes over follow-up 60
S10.14 ODYSSEY A - Triglycerides changes over follow-up 60
S10.15 Total population - changes in Triglycerides over follow-up 61
S10.16 ODYSSEY A - changes in Triglycerides over follow-up 62
S10.17 Total population - Glucose changes over follow-up 63
S10.18 ODYSSEY A - Glucose changes over follow-up 63
S10.19 Total population - changes in Glucose over follow-up 64
S10.20 ODYSSEY A - changes in Glucose over follow-up 65
S11 Adverse Events 66
S11.1 Serious Adverse Events (SAEs) to trial censoring date by SAE type* 66
S11.2 Serious Adverse Events to trial censoring date 67
S11.3 Grade 3 or above clinical and laboratory adverse events to trial censoring date 69

S11.4 Adverse events leading to ART modification (any grade) to trial censoring date ... 71
S11.5 Serious Adverse Events (SAEs) to week 96 by SAE type* ... 71
S11.6 Serious Adverse Events to week 96 ... 72
S11.7 Grade 3 or above clinical and laboratory adverse events to week 96 ... 74
S11.8 Adverse events leading to ART modification (any grade) to week 96 ... 76
S12 Patient Reported Outcomes ... 77
S12.1 Summary of carer reported adherence ... 77
S12.2 Summary of carer reported acceptability of treatment during follow-up ... 78
S13 Antiretroviral Therapy ... 79
S13.1 Summary of initial SOC treatment regimens ... 79
S13.2 Summary of initial DTG regimens ... 79
S13.3 Definitions of Treatment Regimens ... 80
S13.4 Treatment regimen at last visit ... 80
S13.5 Details of Changes to Treatment Regimens for Toxicity or Treatment Failure ... 81
S13.6 Details of Other Changes to Treatment Regimens ... 81
S14 Anthropometric measures ... 82
S14.1 Total population - Height (cm) change over follow-up ... 82
S14.2 ODYSSEY A - Height (cm) change over follow-up ... 82
S14.3 Total population - Height (cm) change over follow-up ... 83
S14.4 ODYSSEY A - Height (cm) change over follow-up ... 84
S14.5 Total population - Weight (kg) change over follow-up ... 85
S14.6 ODYSSEY A - Weight (kg) change over follow-up ... 85
S14.7 Total population - Weight (kg) change over follow-up ... 86
S14.8 ODYSSEY A - Weight (kg) change over follow-up ... 87
S14.9 Total population - BMI-for-age change over follow-up ... 88
S14.10 ODYSSEY A - BMI-for-age change over follow-up ... 88
S14.11 Total population - BMI-for-age change over follow-up ... 89
S14.12 ODYSSEY A - BMI-for-age change over follow-up ... 90
S14.13 Total population - Weight-for-age change over follow-up ... 91
S14.14 ODYSSEY A - Weight-for-age change over follow-up ... 91
S14.15 Total population - Weight-for-age change over follow-up ... 92
S14.16 ODYSSEY A - Weight-for-age change over follow-up ... 93
S14.17 Total population - Weight-for-Height change over follow-up ... 94
S14.18 ODYSSEY A - Weight-for-Height change over follow-up ... 94
S14.19 Total population - Weight-for-Height change over follow-up ... 95
S14.20 ODYSSEY A - Weight-for-Height change over follow-up ... 96
S14.21 Weight-for-age z-scores over follow-up in participants with weight-for-age z-score <-3 at enrolment* ... 97
S15 Biochemistry ... 98
S15.1 Total population - Creatinine changes over follow-up ... 98
S15.2 ODYSSEY A - Creatinine over follow-up ... 98
S15.3 Total population - Creatinine changes over follow-up ... 99
S15.4 ODYSSEY A - Creatinine changes over follow-up ... 100

S16 ODYSSEY sites recruiting children to <14kg cohort 101
S17 ODYSSEY inclusion and exclusion criteria 102
S17.1 Patient Inclusion Criteria 102
S17.2 Patient Exclusion Criteria 102

S1 ODYSSEY Trial Team

Penta Foundation: Carlo Giaquinto, Tiziana Grossele, Daniel Gomez-Pena, Davide Bilardi, Giulio Vecchia

MRC CTU at UCL: Shabinah S. Ali, Abdel Babiker, Shazia Begum, Chiara Borg, Anne-Marie Borges Da Silva, Joanna Calvert, Man Chan, Nimisha Dudakia, Deborah Ford, Joshua Gasa, Diana M. Gibb, Nasir Jamil, Sarah Lensen, Emma Little, Fatima Mohamed, Samuel Montero, Cecilia L. Moore, Rachel Oguntimehin, Anna Parker, Reena Patel, Tasmin Phillips, Tatiana Sarfati, Karen Scott, Clare Shakeshaft, Moira Spyer, Margaret Thomason, Anna Turkova, Rebecca Turner, Nadine Van Looy, Ellen White, Ian White, Kaja Widuch, Helen Wilkes, Ben Wynne

INSERM SC-10-US19--ANRS: Alexandra Compagnucci, Yacine Saidi, Yoann Riault, Alexandra Coelho, Laura Picault, Christelle Kouakam

PHPT: Tim R. Cressey, Suwalai Chalermpantmetagul, Dujrudee Chinwong, Gonzague Jourdain, Rukchanok Peongjakta, Praornsuda Sukrakanchana, Wasna Sirirungsi

Joint Clinical Research Centre, Uganda: Cissy M. Kityo, Victor Musiime, Elizabeth Kaudha, Annet Nanduudu, Emmanuel Mujyambere, Paul Ocitti Labeja, Charity Nankunda, Juliet Ategeka, Peter Erim, Collin Makanga, Esther Nambi, Abbas Lugemwa, Lorna Atwine, Edridah Keminyeto, Deogratiuos Tukwasibwe, Shafic Makumbi, Emily Ninsiima, Mercy Tukamushaba, Rogers Ankunda, Ian Natuhurira, Miriam Kasozi, Baker Rubinga, Diana Antonia Rutebarika, Rashidah Nazzinda, Shamim Nakabuye, Julius Tumusiime, Alice Mulindwa, Ritah Mbabazi, Milly Ndigendawani, Edward Bagirigomwa, Eddie Rubanga, David Eram, Maria Nannungi, Chrispus Katemba, Disan Mulima, Josephine Namusanje, Mariam Nabalamba, Priscilla Kyobutungi, Phyllis Mwesigwa Rubondo, Robinah Kibenge, Claire Nasaazi, Basiimwa Roy Clark, Enock Babu, Alex Musiime, Faith Mbasani, Martin Ojok, Odoch Denis, David Baliruno, Katabalwa Juliet, Benson Ouma, Barbara Ainebyoona, Mariam Naabalamba, Diana A. Rutebarika, Alex V. Musiime, Josephine Kobusungye, Ezra Lutalo

Baylor College of Medicine Children's Foundation, Uganda: Adeodata R. Kekitiinwa, Pauline Amuge, Dickson Bbuye, Justine Nalubwama, Winnie Akobye, Muzamil Nsibuka Kisekka, Anthony Kirabira, Gloria Ninsiima, Sylvia Namanda, Gerald M. Agaba, Immaculate Nagawa, Annet Nalugo, Florence Namuli, Rose J. Kadhuba, Rachael K. Namuddu, Lameck Kiyimba, Angella Baita, Eunice Atim, Olivia Kobusingye, Clementine Namajja, Africanus Byaruhanga, Rogers Besigye, Herbert Murungi, Geoffrey Onen, Lawrence Lekku, Judith Tikabibamu, Henry Balwa

MUJHU Research Collaboration, Uganda: Philippa Musoke, Linda Barlow-Mosha, Grace Ahimbisibwe, Rosemary Namwanje, Hajira Kataike, Mark Ssenyonga, Brenda Kakayi, Rebecca Sakwa, Sarah Nakabuye, Barbara Musoke Nakirya, Gladys Kasangaki, Raymonds Kyambadde, David Balamusani, Winnie Nansamba, Stella Nalusiba, Emmanuel Mayanja, Richard Isabirye, Erinah Kyomukama, Rebecca Wampamba, Mildred Kabasonga, Zaam Zinda Nakawungu, Sarah Babirye, Olivia Kaboggoza, Juliet Nanyonjo, Joanita Nankya Baddokwaya, Alice Elwana, Winfred Kaahwa, Bosco Kafufu, Emmanuel Hakiza, Maria Musisi, Paula Namayanja, Maria Gorreti Nakalema, Robert Serunjogi, Monica Etima, Phionah Kibalama, Joel Maena, Agnes Mary Mugagga, Annet Miwanda, Monica Nolan.

UZCRC, Zimbabwe: James Hakim, Hilda Mujuru, Kusum Nathoo, Mutsa Bwakura-Dangarembizi, Ennie Chidziva, Shepherd Mudzingwa, Secrecy Gondo, Godfrey Musoro, Vivian Mumbiro, Gloria Tinago, Shirley Mutsai, Joy Chimanzi, Columbus Moyo, Ruth Nhema, Misheck Nkalo Phiri, Stuart Chitongo, Joshua Choga, Joyline Bhiri, Wilber Ishemunyoro, Makhosonke Ndlovu, Moses Chitsamatanga, Pia Ngwaru, Tsitsi Gwenzi, Wendy Mapfumo, Dorothy Murungu, Trust Mukanganiki, Prosper Dube, Tapiwa Gwaze, Farai Matimba, Tawona Mudzviti, Zivai Mupambireyi, Sibusisiwe Weza, Cleopatra Langa, Sandra Musarurwa, Shamiso Gwande

FAM-CRU, South Africa: Mark F. Cotton, Anita Janse van Rensburg, Marlize Smuts, Catherine Andrea, Sumaya Dadan, Sonja Pieterse, Vinesh Jeaven, Candice Makola, George Fourie, Kurt Smith, Els Dobbels, Peter Zuidewind, Hesti Van Huyssteen, Mornay Isaacs, Georgina Nentsa, Thabisa Ncgaba, Candice MacDonald, Maria Bester, Wilma Orange, Ronelle Arendze, Mark Mulder, Lucille Malgraaf, Ashley Harley, Yasmeen Akhalwaya

PHRU, South Africa: Avy Violari, Nastassja Ramsagar, Afaaf Liberty, Ruth Mathiba, Mandisa Nyati, Haseena Cassim, Lindiwe Maseko, Nkata Kekane, Busi Khumalo, Mirriam Khunene, Noshalaza Sbisi, Jackie Brown, Tryphina Madonsela, Nokuthula

Mbadaliga, Zaakirah Essack, Reshma Lakha, Aasia Vadee, Derusha Frank, Nazim Akoojee, Maletsatsi Monametsi, Gladness Machache, Yolandie Fourie, Anusha Nanan-kanjee, Juan Erasmus, Angelous Mamiane, Tseleng Daniel, Fatima Mayat, Nomfundo Maduna, Patsy Baliram, Sibongile Sithebe, Emily Lebotsa, Siphiwe Mkhize

Klerksdorp Tshepong Hospital Complex, South Africa: Ebrahim Variava, Modiehi Rakgokong, Dihedile Scheppers, Tumelo Moloantoa, Abdul Hamid Kaka, Tshepiso Masienyane, Akshmi Ori, Kgosimang Mmolawa, Pattamukkil Abraham.

Durban International Clinical Research Site, South Africa: Moherndran Archary, Rosie Mngqibisa, Rejoice Mosia, Sajeeda Mawlana, Rashina Nundlal, Penelope Madlala, Allemah Naidoo, Sphiwee Cebekhulu, Petronelle Casey, Subashinie Sidhoo, Minenhle Chikowore, Lungile Nyantsa, Sheleika Singh

AHRI, South Africa: Nigel Klein, Osee Behuhuma, Olivier Koole, Kristien Bird, Nomzamo Buthelezi, Mumsy Mthethwa, Gugu Gasa, Siva Danaviah and Theresa Smit

PHPT CTU: Tim R. Cressey, Suwalai Chalermpantmetagul, Gonzague Jourdain, Nicole Ngo Giang Huong, Dujrudee Chinwong, Chalermpong Saenjum, Rukchanok Peongjakta, Pra-ornsuda Sukrakanchana, Woottichai Khamduang, Laddawan Laomanit, Ampika Kaewbundit, Jiraporn Khamkon, Kanchana Than-in-at, Sanuphong Chailert, Worathip Sripaoraya, Nitinart.krueduangkam, Namthip Kruenual, Warunee Khamjakkaew, Soraya Klinprung, **Prapokklao Hospital:** Chaiwat Ngampiyaskul, Pisut Greetanukroh, Praechadaporn Khannak, Pathanee Tearsansern, Wanna Chamjamrat, **Phayao Hospital, Thailand:** Nuttawut Chanto, Thitiwat Thapwai, Khanungnit Thungkham, Patcharee Puangmalai, Chutima Ruklao, **Chiangrai Prachanukroh Hospital, Thailand:** Pradthana Ounchanum, Suwimon Khusuwan, Sukanda Denjanta, Yupawan Thaweesombat, Jutarat Thewsoongnoen, Kanyanee Kaewmamueng, Phakamas Kamboua, Supawadee Pongprapass (Sangjan), Warunee Srisuk, Areerat Kongponoi, Juthamas Limplertjareanwanich, **Nakornping Hospital, Thailand:** Suparat Kanjanavanit, Prattana Leenasirimakul, Chayakorn Saewtrakool, Pacharaporn Yingyong, Duangrat Chutima (Suwan), Raungwit Junkaew, Orapin Khatngam, Thannapat Chankun, **Khon Kaen Hospital, Thailand:** Ussanee Srirompotong, Patamawadee Sudsaard, Sookpanee Wimonklang, Turian Petpranee, **Mahasarakam Hospital, Thailand:** Sathaporn Na-Rajsima, Pattira Runarassamee, Nuananong Kunjaroenrut, Arttasid Udomvised, Tassawan Khayanchoomnoom, Watchara Meethaisong, Ketmookda Trairat **HIVNAT, Thailand:** Thanyawee Puthanakit, Suvaporn Anugulruengkitt, Wipaporn Natalie Songtaweesin, Torsak Bunupuradah, Naruporn Kasipong, Sararut Chanthaburanun, Apicha Mahanontharit, Kesdao Nanthapisal, Thidarat Jupimai, Thornthun Noppakaorattanamanee, Chutima Saisaengjan

European Site Investigators: Goethe University Frankfurt**, Germany:** Stephan Schultze-Strasser, Christoph Königs , **UKE Eppendorf, Germany:** Robin Kobbe, Ulf Schulze-Sturm, Felicia Mantkowski, Cornelius Rau, **Heartlands Hospital, UK:** Steve Welch, Jacqui Daglish, Laura Thrasyvoulou, Kate Gandhi, Yvonne Vaughan-Gordon, **Great Ormand Street Hospital, UK:** Delane Singadia, Sophie Foxall, Judith Acero, Malgorzata Pasko-Szcech, Jacquie Flynn, **St Mary's Hospital, UK:** Gareth Tudor-Williams, Amina Farhana Mehar, Caroline Foster, Sobia Mustafa, **Leicester Royal Infirmary, UK:** Srini Bandi, Jin Li, Jackie Philps, **Leeds General Infirmary, UK:** Sean O'Riordan, Dominique Barker, Richard Vowden, Maria Dowie **Kings College Hospital, UK:** Colin Ball Eniola Nsirim, Kathleen McClaughlin, **Hospital 12 de Octubre, Spain:** India Garcia, Pablo Rojo Conejo, Cristina Epalza, Luis Prieto Tato, Maite Fernandez, Luis Escosa Garcia, **Hospital La Paz, Spain:** Maria José Mellado Peña, Talia Sainz Costa, **Hospital San Joan de Déu, Spain:** Claudia Fortuny Guasch, Antoni Noguera Julian, Carolina Estepa, Elena Bruno, Patricia Mendez Garcia, Alba Murciano Cabeza, Biobanco Gregorio Maranon, Maria Angeles Muñoz Fernandez, Jose Luis Jimenez, Coral Gomez Rico, **Centro Materno-infantile do Norte, Portugal:** Laura Marques, Carla Teixeira, Alexandre Fernandes, Rosita Nunes, Helena Nascimento, Andreia Padrao, Joana Tuna, Helena Ramos, Ana Constança Mendes, Helena Pinheiro, Ana Cristina Matos

Local Site Monitors: Flavia Kyomuhendo, Sarah Nakalanzi, Cynthia Mukisa Williams, Leora Sewnarain, Ntombenhle Ngcobo, Deborah Pako, Nompumelelo Yende, Jacky Crisp, Marlize Smuts, Benedictor Dube, Precious Chandiwana, Winnie Gozhora, Thidarat Jumpimai

PK substudies: David Burger, Pauline Bollen, Angela Colbers, Hylke Waalewijn, Tom Jacobs

Virology-immunology substudy: Nigel Klein, Eleni Nastouli, Anita De Rossi, Maria Angeles Munoz Fernandez, Carlota Miranda, Moira Spyer,

Social Science substudy and Youth Trial Board project: Janet Seeley, Sarah Bernays, Stella Namukwaya, Zivai Mupambireyi, Magda Conway, Lungile Jafta, Mercy Shibemba

Independent Trial Steering Committee Members: Ian Weller, Elaine Abrams, Tsitsi Apollo, Polly Clayden, Valériane Leroy

Independent Data Monitoring Committee Members: Anton Pozniak, Jane Crawley, Rodolphe Thiébaut, Helen McIlleron

Endpoint Review Committee Members: Alasdair Bamford, Hermione Lyall, Andrew Prendergast, Felicity Fitzgerald, Anna Goodman

S2 Primary and secondary outcome measures in ODYSSEY

**Primary Efficacy Outcome**
Difference in proportion with clinical or virological failure by 96 weeks, defined as the first occurrence of any of the following components:<br>1. Insufficient virological response defined as <1 log10 drop at week 24 (or viral load ≥50c/mL at week 24 in a participant with viral load <500c/mL at baseline) and switch to second/third line antiretroviral therapy for treatment failure<br>2. Virological failure (defined as a viral load of greater than or equal to 400 copies/mL at or after week 36 confirmed by the next visit)<br>3. New or recurrent AIDS defining event (WHO 4) or severe WHO 3 event, confirmed by the Endpoint Review Committee<br>4. All-cause death
**Secondary efficacy outcomes**
Difference in proportion with clinical or virological failure (as defined above) by 48 weeks<br>Time to any new or recurrent AIDS defining event (WHO 4) or severe WHO 3 events, confirmed by the Endpoint Review Committee<br>Proportion of children with Viral Load <50 c/ml at 48 and 96 weeks<br>Proportion of children with Viral Load <400 c/ml at 48 and 96 weeks<br>Rate of HIV-associated events (WHO 4 and severe WHO 3) and death over 96 weeks<br>Change in CD4 count and percentage and CD4/CD8 ratio from baseline to weeks 48 and 96<br>Proportion developing new resistance mutations
**Secondary safety outcomes**
Change in total cholesterol, triglycerides and lipid fractions (high-density lipoproteins**,** low-density lipoproteins) from baseline to weeks 48 and 96 (change in total cholesterol from baseline to week 96 will be used to formally assess superiority of dolutegravir-based regimen vs. standard-of-care)<br>Incidence of serious adverse events<br>Incidence of new clinical and laboratory grade 3 and 4 adverse events<br>Incidence of adverse events (of any grade) leading to treatment modification
**Other secondary outcomes**
Adherence and acceptability

S3 Statistical methods

S3.1 Analysis populations

The <14kg cohort includes participants weighing <14kg, randomised after recruitment to the main trial was completed, with randomisations between the 5th July 2018 and the 26th August 2019. Follow-up was censored on 28th June 2021, when the last child in the <14kg cohort reached 96 weeks follow-up.

Intent-to-treat population

The intent-to-treat (ITT) population includes all randomised participants, except those considered randomised in error. Randomisation in error was judged by whether the participant met a major violation of the eligibility criteria, including the participant was randomised at a time when the dolutegravir dose was not known for the participant's weight (and participant not participating in PK); and did not depend on treatment allocation or post-randomisation follow-up.

Intent-to-treat analyses are performed on all participants in the ITT population and participants are analysed according to the study group to which they were randomised.

Per protocol population

Participants who did not meet the eligibility criteria or where there was a major protocol deviation, e.g. site prescribed incorrect drug, are excluded from this population. Changes to the third agent (i.e. the non-NRTI component, including adding an additional third agent) for toxicity, failure or pregnancy are defined as a change in therapy and participants are censored at this date. Any stop in regimen for >31 days is defined as stopping therapy and participants are censored at this date.

S3.2 Primary endpoint

The primary outcome was the difference in the cumulative probability of virological or clinical failure by 96 weeks, estimated by Kaplan-Meier method using time to the first occurrence of any of the following components:

- Insufficient virological response defined as <1 log10 drop at week 24 (or not <50 at week 24 if VL<500c/ml at baseline) and switch to second/third-line treatment for treatment failure
- VL ≥400 c/ml at or after 36 weeks confirmed by repeat VL
- Death due to any cause
- Any new or recurrent AIDS defining event (WHO 4) or severe WHO 3 event, confirmed by the Endpoint Review Committee

The primary comparison was the DTG-based regimen versus SOC for the primary efficacy outcome in the combined population (first and second line) adjusted for first and second line (ODYSSEY A and B).

Handling viral load data

Participants were included in ODYSSEY both from sites where HIV RNA was routinely measured frequently and from sites where HIV RNA is routinely measured 6-12 monthly. In the latter case, HIV RNA was measured retrospectively on plasma samples stored at each clinic visit. Confirmatory HIV RNA measures were likely to be performed faster in sites where HIV RNA monitoring occurred routinely more often. The primary endpoint included confirmed HIV RNA≥400 c/ml after 36 weeks.

For consistency across sites, where 2 consecutive HIV RNA were ≥400c/ml, the date of the next scheduled visit following the first HIV RNA ≥400 (using schedule from randomisation) was used to calculate the time to the first confirmed viral load measurement ≥400c/ml (primary endpoint failure date), rather than the date of the confirmatory measure. Where a patient had an HIV RNA≥400c/ml and switched treatment for treatment failure without a confirmatory viral load before switch, the date of the next scheduled visit following the first HIV RNA ≥400c/ml was used for the first confirmed viral load measurement ≥400c/ml.

The primary endpoint included insufficient virological response by week 24, defined by less than a 1 log10 drop in viral load from baseline (or HIV RNA≥50c/ml at week 24 when baseline HIV RNA<500c/ml) with switch to second/third line treatment for treatment failure. A participant was considered a failure if they switched to second- or third-line treatment following insufficient virological response at week 24 without suppressing (VL<50c/ml) between week 24 and switching therapy. Date of switching therapy was used as the primary endpoint failure date.

Analysis details for primary efficacy outcome

In the <14kg cohort, time to clinical or virological failure (ODYSSEY A and B combined) was estimated using Kaplan Meier curves to estimate the proportion of children failing in each arm at any time up to the week 96 censoring date (96 weeks from date of randomisation). The survival curve for each combination of ODYSSEY A/B stratum and randomised group was calculated using a Cox model adjusting for ODYSSEY A/B stratum and randomised group. The average survival curve for each randomised group

was estimated as a weighted average of the corresponding stratum-specific survival curves, with weights proportional to the number of individuals in each stratum across both randomised groups at baseline. The mean of these differences at week 96 was the point estimate for the difference in overall survival function between DTG and SOC arm. As only 85 children were recruited weighing <14kg, confidence intervals for the proportions failing by treatment arm were estimated by bootstrap on the log(-log(S(t)) scale, assuming Normal theory. The confidence interval for the risk difference was estimated using bias-corrected bootstrap. Bootstrapping was sampled 1000 times and stratified by ODYSSEY A/B and treatment group.

In the main trial (children enrolled weighing ≥14kg) the treatment effect had been estimated similarly, although stratification was by: trial cohort (ODYSSEY A or B), routine availability of resistance tests (available or unavailable), abacavir and lamivudine NRTI backbone vs. other NRTI backbone.

The primary outcome was assessed using Bayesian analysis, including an interaction parameter to model the relationship between underlying treatment effects in the ≥14kg and <14kg subgroups. Prior to any results being shared outside of IDMC meetings, elicitation was used to obtain clinical opinions on the likely values of the interaction parameter. Expert clinical opinion was elicited from thirteen experienced paediatricians and demonstrated that substantial borrowing from the ≥14kg cohort was supported. Clinical experts chose on average to allocate a relative weight of 78% (reduced from 90% based on sample size) to data from children weighing ≥14kg in a Bayesian analysis of the cohort of children weighing <14kg. The total effective sample size in the Bayesian analysis was 386 children, providing 84% predictive power to exclude a difference of more than 10% between arms, whereas the 85 younger children weighing <14kg provided only 20% power in a standalone frequentist analysis.[2]

Frequentist analyses of the <14kg cohort alone is adjusted for ODYSSEY A/B. The pooled dataset (whereby all data were analysed together is stratified by weight band (≥/<14kg) in addition to the strata identified above for the ≥14kg group and antiretroviral treatment (ODYSSEY A/B) in the <14kg group, i.e. 10 strata:

- ≥14kg/ODYSSEY A/No Resistance Testing/Abacavir and lamivudine NRTI backbone;
- ≥14kg/ODYSSEY A/Resistance Testing/ Abacavir and lamivudine NRTI backbone;
- ≥14kg/ODYSSEY A/No Resistance Testing/other NRTI backbone;
- ≥14kg/ODYSSEY A/Resistance Testing/ other NRTI backbone;
- ≥14kg/ODYSSEY B/No Resistance Testing/Abacavir and lamivudine NRTI backbone;
- ≥14kg/ODYSSEY B/Resistance Testing/Abacavir and lamivudine NRTI backbone;
- ≥14kg/ODYSSEY B/No Resistance Testing/other NRTI backbone;
- ≥14kg/ODYSSEY B/Resistance Testing/other NRTI backbone;
- <14kg/ODYSSEY A;
- <14kg/ODYSSEY B.

The Kaplan-Meier plot for time to clinical or virological failure follows the KMunicate principles.[3] The risk table presented differs to that proposed initially by Morris et al., where here 'at-risk (no event)' includes those observed without event up to and including time t; 'censored' includes those censored before time t; and 'event' includes those with an event up to and including time t. Using this approach, we assume that the outcome is known before censoring where both occur on same day.

1. Reference: Turkova A, White E, Mujuru HA, Kekitiinwa AR, Kityo CM, Violari A, et al. ODYSSEY: Dolutegravir For First- and Second-Line HIV Treatment in Children. New England Journal of Medicine. 2021.
2. Reference: Turner B, Ford D, Moore C, Gibb D, Turkova A, White I, et al. Analysing small groups within clinical trials, while borrowing information from larger groups. Abstract presented at the 40th Annual Conference of the International Society for Clinical Biostatics; July 16, 2019; Leuven, Belgium.
3. Reference: Morris TP, Jarvis CI, Cragg W, Phillips PPJ, Choodari-Oskooei B, Sydes MR. Proposals on Kaplan–Meier plots in medical research and a survey of stakeholder views: KMunicate. BMJ Open 2019;9:e030215. doi: 10.1136/bmjopen-2019-030215

Per protocol

The per protocol analysis aimed to estimate the "while on treatment" effect,[4] by comparing the primary endpoint between arms in the per protocol population (described above in S3.1).

4. Reference: ICH E9 (R1) addendum on estimands and sensitivity analysis in clinical trials to the guideline on statistical principles for clinical trials. EMA/CHMP/ICH/436221/2017

Sub-group analysis

A sub-group analysis was completed by age at randomisation (</>=1 year).

S3.3 FDA snapshot algorithm

Definitions for FDA snapshot algorithm

The FDA snapshot algorithm in ODYSSEY was used to compare virological suppression (<50 c/ml and <400 c/ml, indicated by Y) in DTG vs. SOC at weeks 48 and 96 (indicated by X).

Here we define changes to the initial ART regimen for the purposes of the FDA snapshot algorithm:

- **Strict initial regimen** - the strict initial regimen assigned at randomisation to ODYSSEY;
- **"Ignored" ART changes** – changes to NRTI backbone for increases in age/weight, simplification (including patient/carer decisions), and drug availability. Changes to 3rd agent are ignored when: (1) DTG arm – changes to the DTG dose for increases in age/weight; (2) SOC arm - changes to 3rd agent for simplification (including patient/carer decision), drug availability, and increases in weight.
- **"Permitted" ART changes** include: changes to NRTI backbone for any reason other than those listed above and treatment failure.
- **"Non-permitted" ART changes** include: (i) changes to the NRTI backbone in the initial regimen for treatment failure; (ii) changes to the third agent for treatment failure; (iii) changes to third agent for toxicity; or (iv) changes to the third agent for all reasons not listed in "Ignored ART changes".

Only post-baseline and on-treatment viral loads were used in the algorithm. Participants interrupting ART prior to or within week X window were assumed to remain on the regimen and were treated as a treatment switch when a new regimen (according to rules above) was initiated (although, viral loads off treatment were not used). In the case where a participant had multiple viral loads within week X window, the latest viral load was used in the FDA snapshot algorithm, provided it was on/prior to trial censoring date (28th June 2021).

S3.4 Flowchart of the FDA snapshot algorithm

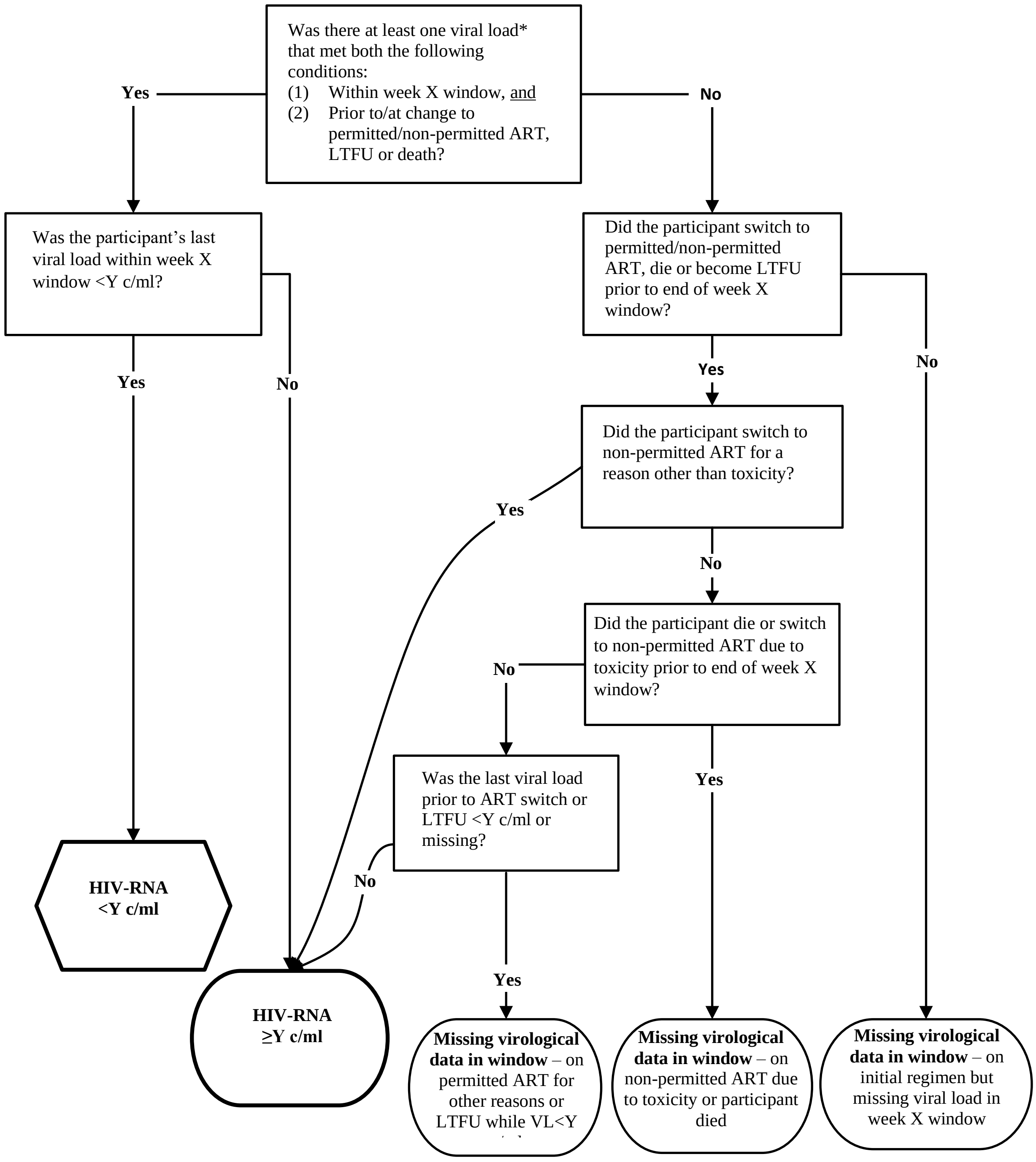

*Off ART viral loads will be excluded from algorithm.

S3.5 Analysis of continuous outcomes

Changes in continuous outcomes were analysed using analysis of covariance, adjusting for baseline value and ODYSSEY A/B; average treatment differences through follow-up were estimated using mixed linear models with random effect for intercept and fixed effects for treatment arm, visit week and ODYSSEY A/B.

S3.6 Analyses of clinical events

Clinical events (WHO severe stage 3 and stage 4 events and adverse events) were compared between arms using the hazard ratio for first event from a Cox proportional hazards regression model.

S3.7 Definition of treatment change

The following are used to define substitutions and changes to initial ART regimens (Appendix, S13):

A strict initial regimen was defined as strictly the regimen allocated at trial entry.

A substituted initial regimen was defined as the strict initial regimen allowing for substitutions to the third agent of the regimen for reasons other than toxicity, treatment failure, pregnancy or major protocol deviation (e.g. site prescribing incorrect drug) or to the NRTI component for any reason. Permissible substitutions to the third agent included reasons for simplification, drug availability, patient/carer decision and increases in weight.

All reductions to NRTIs were also considered substituted initial regimen.

All changes to the third agent not defined above as a substitution to the initial regimen were considered a change from the initial treatment regimen. In the DTG arm, DTG should only be stopped for toxicity, failure or (at clinician's discretion) pregnancy.

Changes to the third agent for reasons of treatment failure were defined as switching to second line therapy in ODYSSEY A or third line therapy in ODYSSEY B.

Any interruptions or reductions to a regimen for less than or equal to 31 consecutive days were allowed; as long as the total number of drugs allocated in the regimen was not increased.

A stop to the initial regimen occurred when a patient interrupted their regimen or their third agent for >31 days.

S3.8 Adjustment and testing multiple outcomes

Unless otherwise specified, adjusted analyses adjust for ODYSSEY A/B and are used in the paper. Unadjusted analyses are also presented through the Supplementary Appendix.

In accordance with the statistical analysis plan, we have made no adjustment to p-values or confidence intervals to allow for testing multiple secondary outcomes.

S4 Impact of Covid-19

Due to the COVID-19 pandemic (March 2020 onwards), and uncertainty about how it would evolve, sites were advised that all participants would remain in randomised follow-up until the final participant recruited <14kg reached 96 weeks follow-up on the 28th June 2021. It was originally intended that participants recruited <14kg would exit randomised follow-up upon reaching week 96.

There may have been a slight excess of missed in person visits between 1st March-31st May 2020 (10 visits missed, 11% of expected visits) and 1st June-31st August 2020 (4 visits missed, 5% of expected visits) compared to the remainder of follow-up (total 2% of expected visits missed). Missed visits within the period 1st March-31st August 2020 were similar in both arms with 8 in DTG and 6 in SOC (DTG 10%; SOC 7%). Two of the 14 missed in-person visits were conducted over the phone (both in SOC).

In total, there were 11 participants with missed in person visits within the period 1st March-31st August 2020, 6 in DTG and 5 in SOC. Of these 11 participants, 1 in SOC was withdrawn (December 2019 for social problems) and 1 in DTG was lost to follow-up (December 2019 for carer relocation). In addition, one participant was withdrawn in August 2020 for moved area. None of these withdrawals or losses to follow-up appear to be related to COVID-19. Of the 9 participants with any missed in person visit within the period 1st March-31st August 2020 who remained in follow-up to the trial censoring date, only one participant missed more than one consecutive visit and they were suppressed at week 96. Drug supply was ensured where in person visits were missed.

S5 Dolutegravir dosing throughout ODYSSEY

	ODYSSEY v2.0*	ODYSSEY v3.0		ODYSSEY v4.0		ODYSSEY v5.0 onwards	
	Main trial participants	Main trial participants	WB PK substudy participants	Main trial participants	WB PK substudy participants	Main trial participants	WB PK substudy participants
3-<6kg	-	-		-	5mg or 10mg DT[ψ]	-	5mg or 10mg DT[ψ]
6-<10kg	-	-		-	15mg DT	-	15mg DT
10-<14kg	-	-		-	20mg DT	-	20mg DT
14-<15kg	-	-	25mg	25mg→25mg DT[§]	25mg DT	25mg DT	N/A
15-<20kg	20mg*	20mg					
20-<25kg	25mg	25mg	25mg	25mg→30mg DT or 50mg[¥]	30mg DT or 50mg†	50mg[π]	N/A
25-<30kg	25mg	25mg→50mg**	50mg	25mg→50mg**	NA	50mg	NA
30-<35kg	35mg	35mg→50mg**	50mg	35mg→50mg**	NA	50mg	NA
35-<40kg	35mg	35mg→50mg**	50mg	35mg→50mg**	NA	50mg	NA
≥40kg	50mg	50mg	NA	50mg	NA	50mg	NA

ψ Infants <6 months of age receive DTG 5mg QD while infants ≥6 months of age receive DTG 10mg QD, both as dispersible tablets.
* In May 2017 the EMA licensed the use of 20mg DTG in children 15 - <20kg and ≥6years, following this, children were able to be recruited in this weight and age-band.
** From 1st of April 2018, after ethics notification, sites following protocol version 3.0 and above were recommended to increase the DTG dose of children 25 - <40kg to 50mg FCT QD at their next scheduled study visit based on the results of the WB-PK2. WB-PK2 participants remained on DTG 50mg with ongoing follow-up. Non-PK participants recruited after implementation were initiated on the 50mg film-coated DTG dose.
† Both doses are examined in WB-PK1 part II substudy in this weight-band.

§ Children 15 - <20kg previously receiving DTG 20mg QD were changed to DTG film-coated 25mg tablets upon the approval of protocol v4.0. Subsequently all children 14-<20kg changed to DTG 25mg QD dispersible tablets following the review of WB-PK1 part I results and approval by the relevant ethical and regulatory authorities.
¥ Children 20 - <25kg previously receiving DTG 25mg QD as one 25mg film-coated tablet changed to either DTG 30mg QD dispersible tablets or DTG 50mg QD (film-coated tablet) depending on site following the review of WB-PK1 part I results and approval by the relevant ethical and regulatory authorities.
π Following the review of PK and safety data children 20-<25kg receiving DTG 30mg dispersible tablets should be switched to DTG 50mg film-coated tablets. Those who prefer to remain on DTG 30mg DT will be able to do so until they move weight band.
DT=dispersible, otherwise film coated tables

S6 Enrolment and Eligibility

S6.1 Screening and randomisation

	Total	A	B
Screened	102	80	22
Randomised	85	72	13
Total ineligible	17	8	9
Reasons for ineligibility			
Failed at least one eligibility criterion	14	5	9
Died before enrolment	1	1	0
Other+	2	2	0

+1 ODYSSEY A participant's carer declined to participate due to the blood volumes required. 1 ODYSSEY A participant was not recruited as the protocol amendment allowing recruitment to this participant's weight group had not been approved at the site.

S7 Baseline Demographics

S7.1 Baseline characteristics

	Total			A			B		
	**DTG**	**SOC**	**Total**	**DTG**	**SOC**	**Total**	**DTG**	**SOC**	**Total**
**Participants randomised**	42	43	85	35	37	72	7	6	13
**Country**									
South Africa	8 (19%)	12 (28%)	20 (24%)	8 (23%)	12 (32%)	20 (28%)	0 (0%)	0 (0%)	0 (0%)
Uganda	22 (52%)	21 (49%)	43 (51%)	17 (49%)	16 (43%)	33 (46%)	5 (71%)	5 (83%)	10 (77%)
Zimbabwe	12 (29%)	10 (23%)	22 (26%)	10 (29%)	9 (24%)	19 (26%)	2 (29%)	1 (17%)	3 (23%)
**Sex**									
male	16 (38%)	25 (58%)	41 (48%)	14 (40%)	21 (57%)	35 (49%)	2 (29%)	4 (67%)	6 (46%)
female	26 (62%)	18 (42%)	44 (52%)	21 (60%)	16 (43%)	37 (51%)	5 (71%)	2 (33%)	7 (54%)
**Age (years)**									
n	42	43	85	35	37	72	7	6	13
mean (SD)	1.6 (1.2)	1.5 (1.1)	1.5 (1.2)	1.3 (1.2)	1.3 (1.1)	1.3 (1.1)	2.7 (0.5)	2.7 (0.5)	2.7 (0.5)
median	1.3	1.5	1.4	1.2	1.1	1.1	2.6	2.7	2.6
[IQR]	[0.5, 2.0]	[0.6, 2.1]	[0.6, 2.0]	[0.4, 2.0]	[0.5, 1.7]	[0.5, 1.9]	[2.1, 3.2]	[2.5, 2.9]	[2.5, 2.9]
[range]	[0.3-5.9]	[0.1-4.5]	[0.1-5.9]	[0.3-5.9]	[0.1-4.5]	[0.1-5.9]	[2.0-3.3]	[1.9-3.5]	[1.9-3.5]
<6months	11 (26%)	8 (19%)	19 (22%)	11 (31%)	8 (22%)	19 (26%)	0 (0%)	0 (0%)	0 (0%)
6months-<1year	5 (12%)	8 (19%)	13 (15%)	5 (14%)	8 (22%)	13 (18%)	0 (0%)	0 (0%)	0 (0%)
1-<3years	22 (52%)	22 (51%)	44 (52%)	17 (49%)	17 (46%)	34 (47%)	5 (71%)	5 (83%)	10 (77%)
3-<6years	4 (10%)	5 (12%)	9 (11%)	2 (6%)	4 (11%)	6 (8%)	2 (29%)	1 (17%)	3 (23%)
**Weight (kg)+**									
n	42	43	85	35	37	72	7	6	13
mean (SD)	8.0 (2.7)	8.1 (3.0)	8.1 (2.8)	7.4 (2.5)	7.7 (2.9)	7.6 (2.7)	10.7 (1.6)	10.8 (1.6)	10.7 (1.6)
median	8.1	8.2	8.1	6.6	7.3	7.2	11.1	11.3	11.1
[IQR]	[5.6, 10.0]	[5.2, 10.3]	[5.4, 10.0]	[5.3, 9.8]	[5.1, 9.7]	[5.1, 9.7]	[9.0, 12.0]	[9.7, 11.7]	[9.5, 11.7]
[range]	[3.8-13.0]	[3.4-13.4]	[3.4-13.4]	[3.8-13.0]	[3.4-13.4]	[3.4-13.4]	[8.8-13.0]	[8.0-12.5]	[8.0-13.0]
<6kg	11 (26%)	12 (28%)	23 (27%)	11 (31%)	12 (32%)	23 (32%)	0 (0%)	0 (0%)	0 (0%)
6-<10kg	20 (48%)	20 (47%)	40 (47%)	17 (49%)	18 (49%)	35 (49%)	3 (43%)	2 (33%)	5 (38%)
10-<14kg	11 (26%)	11 (26%)	22 (26%)	7 (20%)	7 (19%)	14 (19%)	4 (57%)	4 (67%)	8 (62%)
**Height (cm)**									
n	42	43	85	35	37	72	7	6	13
mean (SD)	71.9 (12.1)	72.0 (11.5)	71.9 (11.7)	69.0 (10.8)	70.6 (11.7)	69.8 (11.3)	86.4 (5.6)	80.4 (4.4)	83.6 (5.8)
median	72.3	73.3	73.0	68.2	70.0	69.7	86.2	80.5	83.5
[IQR]	[60.0, 80.3]	[62.0, 80.5]	[61.0, 80.3]	[58.8, 76.3]	[61.0, 78.2]	[59.5, 77.2]	[80.3, 91.0]	[78.0, 83.5]	[79.0, 86.2]
[range]	[53.5-93.8]	[50.0-93.4]	[50.0-93.8]	[53.5-93.8]	[50.0-93.4]	[50.0-93.8]	[79.0-93.8]	[74.0-86.2]	[74.0-93.8]
**Weight-for-Age***									
n	42	43	85	35	37	72	7	6	13
mean (SD)	-2.3 (1.4)	-2.1 (1.7)	-2.2 (1.6)	-2.3 (1.4)	-2.1 (1.8)	-2.2 (1.6)	-1.8 (1.0)	-1.9 (1.7)	-1.9 (1.3)
median	-2.1	-1.8	-1.9	-2.5	-1.8	-1.9	-1.8	-1.2	-1.6
[IQR]	[-3.4, -1.3]	[-3.7, -0.8]	[-3.4, -1.1]	[-3.5, -1.1]	[-3.4, -0.9]	[-3.5, -1.1]	[-2.8, -1.5]	[-3.7, -0.7]	[-2.8, -1.0]
[range]	[-5.1-0.3]	[-5.7-1.4]	[-5.7-1.4]	[-5.1-0.3]	[-5.7-1.4]	[-5.7-1.4]	[-3.0-0.0]	[-4.3--0.3]	[-4.3-0.0]
<-3	14 (33%)	13 (30%)	27 (32%)	14 (40%)	11 (30%)	25 (35%)	0 (0%)	2 (33%)	2 (15%)
-3-<-2	7 (17%)	6 (14%)	13 (15%)	4 (11%)	6 (16%)	10 (14%)	3 (43%)	0 (0%)	3 (23%)
-2-<0	18 (43%)	21 (49%)	39 (46%)	15 (43%)	17 (46%)	32 (44%)	3 (43%)	4 (67%)	7 (54%)
>=0	3 (7%)	3 (7%)	6 (7%)	2 (6%)	3 (8%)	5 (7%)	1 (14%)	0 (0%)	1 (8%)
**BMI-for-Age***									
n	42	43	85	35	37	72	7	6	13
mean (SD)	-0.9 (1.4)	-0.9 (1.9)	-0.9 (1.7)	-0.9 (1.5)	-1.1 (1.9)	-1.0 (1.7)	-1.1 (1.0)	0.6 (1.3)	-0.3 (1.4)
median	-1.1	-0.7	-0.8	-1.1	-0.8	-0.9	-1.4	0.3	-0.0
[IQR]	[-1.9, 0.2]	[-2.2, 0.3]	[-2.0, 0.2]	[-1.9, 0.2]	[-2.3, 0.2]	[-2.1, 0.2]	[-2.0, -0.0]	[0.0, 0.8]	[-1.4, 0.2]

[range]	[-3.6-1.9]	[-5.7-3.1]	[-5.7-3.1]	[-3.6-1.9]	[-5.7-2.3]	[-5.7-2.3]	[-2.3-0.2]	[-0.7-3.1]	[-2.3-3.1]
<-3	3 (7%)	6 (14%)	9 (11%)	3 (9%)	6 (16%)	9 (13%)	0 (0%)	0 (0%)	0 (0%)
-3-<-2	6 (14%)	6 (14%)	12 (14%)	5 (14%)	6 (16%)	11 (15%)	1 (14%)	0 (0%)	1 (8%)
-2-<0	20 (48%)	14 (33%)	34 (40%)	15 (43%)	13 (35%)	28 (39%)	5 (71%)	1 (17%)	6 (46%)
>=0	13 (31%)	17 (40%)	30 (35%)	12 (34%)	12 (32%)	24 (33%)	1 (14%)	5 (83%)	6 (46%)
**Weight-for-Height***									
n	41	43	84	34	37	71	7	6	13
mean (SD)	-0.9 (1.4)	-0.9 (1.9)	-0.9 (1.7)	-0.8 (1.5)	-1.1 (1.9)	-1.0 (1.7)	-1.2 (0.9)	0.1 (1.4)	-0.6 (1.3)
median	-0.7	-0.8	-0.8	-0.5	-1.1	-0.8	-1.5	-0.1	-0.7
[IQR]	[-1.9, 0.1]	[-1.9, 0.5]	[-1.9, 0.3]	[-1.7, 0.3]	[-1.9, 0.5]	[-1.9, 0.3]	[-1.9, -0.4]	[-0.7, 0.4]	[-1.5, 0.1]
[range]	[-3.4-2.2]	[-5.4-2.6]	[-5.4-2.6]	[-3.4-2.2]	[-5.4-2.2]	[-5.4-2.2]	[-2.4-0.1]	[-1.5-2.6]	[-2.4-2.6]
<-3	3 (7%)	5 (12%)	8 (10%)	3 (9%)	5 (14%)	8 (11%)	0 (0%)	0 (0%)	0 (0%)
-3-<-2	6 (15%)	4 (9%)	10 (12%)	5 (15%)	4 (11%)	9 (13%)	1 (14%)	0 (0%)	1 (8%)
-2-<0	21 (51%)	17 (40%)	38 (45%)	16 (47%)	14 (38%)	30 (42%)	5 (71%)	3 (50%)	8 (62%)
>=0	11 (27%)	17 (40%)	28 (33%)	10 (29%)	14 (38%)	24 (34%)	1 (14%)	3 (50%)	4 (31%)
missing	1	0	1	1	0	1	0	0	0
**Mode of infection**									
Mother to child	42 (100%)	42 (98%)	84 (99%)	35 (100%)	36 (97%)	71 (99%)	7 (100%)	6 (100%)	13 (100%)
Unknown	0 (0%)	1 (2%)	1 (1%)	0 (0%)	1 (3%)	1 (1%)	0 (0%)	0 (0%)	0 (0%)
**Ethnic origin**									
Black-African	41 (98%)	42 (98%)	83 (98%)	34 (97%)	36 (97%)	70 (97%)	7 (100%)	6 (100%)	13 (100%)
Black-other	0 (0%)	1 (2%)	1 (1%)	0 (0%)	1 (3%)	1 (1%)	0 (0%)	0 (0%)	0 (0%)
Mixed Black-White	1 (2%)	0 (0%)	1 (1%)	1 (3%)	0 (0%)	1 (1%)	0 (0%)	0 (0%)	0 (0%)
**CD4%****									
n	41	40	81	34	34	68	7	6	13
mean (SD)	25 (12)	22 (12)	24 (12)	25 (12)	21 (11)	23 (12)	25 (12)	28 (13)	26 (12)
median	24	23	23	24	22	23	28	26	27
[IQR]	[17, 35]	[14, 30]	[16, 31]	[18, 35]	[13, 31]	[16, 32]	[13, 40]	[19, 28]	[15, 29]
[range]	[3- 46]	[1- 53]	[1- 53]	[3- 46]	[1- 43]	[1- 46]	[13- 40]	[14- 53]	[13- 53]
<10	3 (7%)	8 (20%)	11 (14%)	3 (9%)	8 (24%)	11 (16%)	0 (0%)	0 (0%)	0 (0%)
10-<15	4 (10%)	3 (8%)	7 (9%)	2 (6%)	2 (6%)	4 (6%)	2 (29%)	1 (17%)	3 (23%)
15-<20	9 (22%)	4 (10%)	13 (16%)	8 (24%)	3 (9%)	11 (16%)	1 (14%)	1 (17%)	2 (15%)
20-<25	6 (15%)	11 (28%)	17 (21%)	6 (18%)	10 (29%)	16 (24%)	0 (0%)	1 (17%)	1 (8%)
25-<30	7 (17%)	3 (8%)	10 (12%)	5 (15%)	1 (3%)	6 (9%)	2 (29%)	2 (33%)	4 (31%)
30-<40	4 (10%)	9 (23%)	13 (16%)	4 (12%)	9 (26%)	13 (19%)	0 (0%)	0 (0%)	0 (0%)
>=40	8 (20%)	2 (5%)	10 (12%)	6 (18%)	1 (3%)	7 (10%)	2 (29%)	1 (17%)	3 (23%)
missing	1	3	4	1	3	4	0	0	0
**CD4 (cells/mm3)****									
n	41	40	81	34	34	68	7	6	13
mean (SD)	1671 (971)	1425 (1108)	1550 (1042)	1685 (1013)	1408 (1119)	1547 (1068)	1604 (800)	1523 (1140)	1567 (929)
median	1639	1221	1391	1662	1221	1421	1639	1145	1315
[IQR]	[1026, 2327]	[633, 1870]	[863, 2060]	[949, 2327]	[559, 1858]	[839, 2102]	[1026, 2558]	[863, 1882]	[976, 1882]
[range]	[67-4676]	[54-5300]	[54-5300]	[67-4676]	[54-5300]	[54-5300]	[510-2672]	[465-3638]	[465-3638]
50-<100	1 (2%)	4 (10%)	5 (6%)	1 (3%)	4 (12%)	5 (7%)	0 (0%)	0 (0%)	0 (0%)
100-<200	2 (5%)	0 (0%)	2 (2%)	2 (6%)	0 (0%)	2 (3%)	0 (0%)	0 (0%)	0 (0%)
200-<350	1 (2%)	0 (0%)	1 (1%)	1 (3%)	0 (0%)	1 (1%)	0 (0%)	0 (0%)	0 (0%)
350-<500	1 (2%)	3 (8%)	4 (5%)	1 (3%)	2 (6%)	3 (4%)	0 (0%)	1 (17%)	1 (8%)
500-<1000	5 (12%)	7 (18%)	12 (15%)	4 (12%)	5 (15%)	9 (13%)	1 (14%)	2 (33%)	3 (23%)
1000-1500	8 (20%)	11 (28%)	19 (23%)	6 (18%)	10 (29%)	16 (24%)	2 (29%)	1 (17%)	3 (23%)
>=1500	23 (56%)	15 (38%)	38 (47%)	19 (56%)	13 (38%)	32 (47%)	4 (57%)	2 (33%)	6 (46%)
missing	1	3	4	1	3	4	0	0	0
**CD4/CD8 ratio****									
n	41	40	81	34	34	68	7	6	13
mean (SD)	0.7 (0.6)	0.6 (0.5)	0.7 (0.6)	0.8 (0.7)	0.6 (0.4)	0.7 (0.6)	0.7 (0.4)	0.9 (0.9)	0.8 (0.7)
median	0.5	0.6	0.5	0.5	0.5	0.5	0.7	0.7	0.7

[IQR]	[0.3, 0.8]	[0.3, 0.8]	[0.3, 0.8]	[0.4, 0.8]	[0.3, 0.8]	[0.3, 0.8]	[0.2, 1.1]	[0.5, 0.8]	[0.3, 0.8]
[range]	[0.0-2.7]	[0.0-2.8]	[0.0-2.8]	[0.0-2.7]	[0.0-1.3]	[0.0-2.7]	[0.2-1.1]	[0.3-2.8]	[0.2-2.8]
>=1	10 (24%)	7 (18%)	17 (21%)	8 (24%)	6 (18%)	14 (21%)	2 (29%)	1 (17%)	3 (23%)
<1	31 (76%)	33 (83%)	64 (79%)	26 (76%)	28 (82%)	54 (79%)	5 (71%)	5 (83%)	10 (77%)
missing	1	3	4	1	3	4	0	0	0
**Log10 Viral load (copies/mL)****									
n	42	38	80	35	32	67	7	6	13
mean (SD)	5.1 (1.0)	5.1 (1.1)	5.1 (1.0)	5.2 (1.0)	5.3 (1.0)	5.2 (1.0)	4.8 (0.7)	4.1 (1.1)	4.5 (0.9)
median	5.2	5.4	5.3	5.2	5.5	5.5	4.8	4.2	4.8
[IQR]	[4.4, 5.8]	[4.8, 5.9]	[4.6, 5.9]	[4.4, 6.0]	[5.0, 5.9]	[4.8, 5.9]	[4.3, 5.2]	[3.3, 4.8]	[4.1, 4.9]
[range]	[2.7-6.6]	[2.1-7.0]	[2.1-7.0]	[2.7-6.6]	[2.1-7.0]	[2.1-7.0]	[4.1-6.1]	[2.9-5.6]	[2.9-6.1]
**Viral load (copies/mL)****									
<400	0 (0%)	1 (3%)	1 (1%)	0 (0%)	1 (3%)	1 (1%)	0 (0%)	0 (0%)	0 (0%)
400-<1,000	2 (5%)	2 (5%)	4 (5%)	2 (6%)	1 (3%)	3 (4%)	0 (0%)	1 (17%)	1 (8%)
1,000-<10,000	2 (5%)	4 (11%)	6 (8%)	2 (6%)	2 (6%)	4 (6%)	0 (0%)	2 (33%)	2 (15%)
10,000-<50,000	9 (21%)	2 (5%)	11 (14%)	6 (17%)	2 (6%)	8 (12%)	3 (43%)	0 (0%)	3 (23%)
50,000-<100,000	4 (10%)	3 (8%)	7 (9%)	2 (6%)	1 (3%)	3 (4%)	2 (29%)	2 (33%)	4 (31%)
100,000-<500,000	12 (29%)	15 (39%)	27 (34%)	11 (31%)	14 (44%)	25 (37%)	1 (14%)	1 (17%)	2 (15%)
500,000-<1,000,000	5 (12%)	6 (16%)	11 (14%)	5 (14%)	6 (19%)	11 (16%)	0 (0%)	0 (0%)	0 (0%)
>=1,000,000	8 (19%)	5 (13%)	13 (16%)	7 (20%)	5 (16%)	12 (18%)	1 (14%)	0 (0%)	1 (8%)
missing	0	5	5	0	5	5	0	0	0
**History of WHO staging**									
stage1	20 (48%)	16 (37%)	36 (42%)	18 (51%)	14 (38%)	32 (44%)	2 (29%)	2 (33%)	4 (31%)
stage2	11 (26%)	9 (21%)	20 (24%)	7 (20%)	7 (19%)	14 (19%)	4 (57%)	2 (33%)	6 (46%)
stage3	6 (14%)	8 (19%)	14 (16%)	6 (17%)	8 (22%)	14 (19%)	0 (0%)	0 (0%)	0 (0%)
stage4	5 (12%)	10 (23%)	15 (18%)	4 (11%)	8 (22%)	12 (17%)	1 (14%)	2 (33%)	3 (23%)
**Total cholesterol (mg/dL)**									
n	41	36	77	34	30	64	7	6	13
mean (SD)	124 (41)	132 (32)	128 (37)	125 (38)	130 (33)	128 (36)	119 (57)	142 (26)	129 (45)
median	126	139	135	130	137	135	94	155	133
[IQR]	[93, 148]	[106, 157]	[101, 154]	[95, 148]	[104, 154]	[101, 151]	[82, 162]	[115, 159]	[94, 159]
[range]	[50-220]	[65-188]	[50-220]	[50-194]	[65-188]	[50-194]	[51-220]	[104-163]	[51-220]
**Triglycerides (mg/dL)**									
n	41	36	77	34	30	64	7	6	13
mean (SD)	153 (121)	166 (86)	159 (106)	168 (127)	159 (74)	164 (105)	81 (47)	204 (134)	137 (113)
median	127	150	146	140	150	149	63	160	126
[IQR]	[75, 181]	[112, 194]	[82, 181]	[80, 192]	[110, 177]	[94, 189]	[39, 141]	[126, 225]	[63, 149]
[range]	[29-642]	[58-462]	[29-642]	[35-642]	[58-408]	[35-642]	[29-147]	[90-462]	[29-462]
**HDL cholesterol (mg/dL)**									
n	34	33	67	27	27	54	7	6	13
mean (SD)	35 (14)	33 (15)	34 (15)	34 (15)	35 (16)	34 (15)	39 (15)	28 (7)	34 (13)
median	34	35	35	33	39	36	40	28	32
[IQR]	[24, 43]	[24, 43]	[24, 43]	[20, 43]	[24, 44]	[24, 43]	[30, 52]	[21, 35]	[24, 40]
[range]	[8- 60]	[4- 67]	[4- 67]	[8- 58]	[4- 67]	[4- 67]	[16- 60]	[18- 36]	[16- 60]
**LDL cholesterol (mg/dL)**									
n	37	35	72	30	29	59	7	6	13
mean (SD)	71 (32)	69 (31)	70 (31)	72 (28)	67 (31)	69 (29)	67 (49)	77 (32)	72 (41)
median	65	75	68	68	75	69	46	76	51
[IQR]	[48, 84]	[39, 91]	[46, 89]	[54, 84]	[39, 89]	[47, 88]	[33, 100]	[51, 107]	[44, 100]
[range]	[18-166]	[7-128]	[7-166]	[18-145]	[7-128]	[7-145]	[30-166]	[35-119]	[30-166]
**PMTCT Exposure**									
No	19 (45%)	23 (53%)	42 (49%)	16 (46%)	22 (59%)	38 (53%)	3 (43%)	1 (17%)	4 (31%)
Yes	22 (52%)	16 (37%)	38 (45%)	18 (51%)	13 (35%)	31 (43%)	4 (57%)	3 (50%)	7 (54%)
Not Known	1 (2%)	4 (9%)	5 (6%)	1 (3%)	2 (5%)	3 (4%)	0 (0%)	2 (33%)	2 (15%)

*WHO Child Growth Charts and WHO Reference 2007 Charts, version WHO. Metrics are considered missing if height-for-age is <-6/>6, weight-for-age <-6/>5, BMI for age <-5/>5.

+One participant was randomised at 9.7kg; site indicated that they would be dosed as 10-<14kg and therefore they were stratified in this weight-band but are included here according to their actual weight.
**Mean of measurement at screening and randomisation if both are available

S7.2 Baseline characteristics: Antiretroviral exposure [ODYSSEY B Only]

	B					
	DTG		SOC		Total	
**Participants randomised**	7		6		13	
**Number of different drugs ever received excluding PMTCT, median [range]**						
n	7		6		13	
All classes	3.0	[3.0-3.0]	3.0	[1.0-3.0]	3.0	[1.0-3.0]
NRTI	2.0	[2.0-2.0]	2.0	[0.0-2.0]	2.0	[0.0-2.0]
NNRTI	1.0	[0.0-1.0]	0.5	[0.0-1.0]	1.0	[0.0-1.0]
PI	0.0	[0.0-1.0]	0.5	[0.0-1.0]	0.0	[0.0-1.0]
**ART Class Exposure+**						
Monotherapy	0	(0%)	1	(17%)	1	(8%)
NRTI/NNRTI	5	(71%)	2	(33%)	7	(54%)
NRTI/PIs	2	(29%)	3	(50%)	5	(38%)
missing	0		0		0	
**Cumulative ART exposure (years), median [range]**						
n	7		6		13	
All classes	1.6	[1.4-3.0]	1.5	[1.1-1.9]	1.6	[1.1-3.0]
NRTI	1.6	[1.4-3.0]	1.5	[0.0-1.9]	1.6	[0.0-3.0]
NNRTI	1.4	[0.0-3.0]	0.5	[0.0-1.7]	1.4	[0.0-3.0]
PI	0.0	[0.0-1.8]	0.7	[0.0-1.9]	0.0	[0.0-1.9]
**ART Regimen Class Prior to Randomisation+**						
Monotherapy	0	(0%)	1	(17%)	1	(8%)
NRTI/NNRTI	5	(71%)	2	(33%)	7	(54%)
NRTI/PIs	2	(29%)	3	(50%)	5	(38%)
missing	0		0		0	

+Site confirms that NVP was used as a monotherapy prior to the start of ODYSSEY for one participant.

S8 Efficacy

S8.1 Comparison of proportion with clinical or virological failure by 48 weeks

	Total			A			B		
	**DTG**	**SOC**	**DTG vs. SOC**	**DTG**	**SOC**	**DTG vs. SOC**	**DTG**	**SOC**	**DTG vs. SOC**
**Participants randomised**	42	43		35	37		7	6	
**Total participants meeting primary endpoint**	7 (17%)	15 (35%)		4 (11%)	13 (35%)		3 (43%)	2 (33%)	
Insufficient virological response	0 (0%)	0 (0%)		0 (0%)	0 (0%)		0 (0%)	0 (0%)	
Confirmed VL>=400 copies/mL	4 (10%)	11 (26%)		2 (6%)	9 (24%)		2 (29%)	2 (33%)	
Severe WHO 3	0 (0%)	0 (0%)		0 (0%)	0 (0%)		0 (0%)	0 (0%)	
WHO 4	1 (2%)	1 (2%)		0 (0%)	1 (3%)		1 (14%)	0 (0%)	
Death	2 (5%)	3 (7%)		2 (6%)	3 (8%)		0 (0%)	0 (0%)	
**Unadjusted estimated probability of failure**	0.18	0.34		0.13	0.34		0.44	0.33	
[95%CI]	[0.08, 0.37]	[0.22, 0.50]		[0.01, 0.89]	[0.21, 0.52]		[0.01, 1.00]	[0.00, 1.00]	
Difference (DTG-SOC)			-0.16			-0.22			0.11
[95%CI]			[-0.32, 0.01]			[-0.40, -0.03]			[-0.44, 0.57]
P-value**			0.070			0.023			0.65
**Adjusted* estimated probability of failure**	0.18	0.34							
[95%CI]	[0.07, 0.44]	[0.22, 0.50]							
Difference (DTG-SOC)			-0.16						
[95%CI]			[-0.35, -0.00]						
P-value**			0.063						
**A/B x arm interaction+**	0.21					.			

*Adjusted for ODYSSEY A and B strata only in Total. Analysis conducted using same approach as analysis of primary endpoint at 96 weeks in <14kg cohort (see S3.2 statistical methods)
+Z-test

S8.2 Incidence of clinical or virological failure by 48 weeks

	Total			A			B		
	**DTG**	**SOC**	**DTG vs SOC**	**DTG**	**SOC**	**DTG vs SOC**	**DTG**	**SOC**	**DTG vs SOC**
**Participants randomised**	42	43		35	37		7	6	
**Total participants meeting primary endpoint**	7 (17%)	15 (35%)		4 (11%)	13 (35%)		3 (43%)	2 (33%)	
**Person years**	35	36		29	31		6	6	
**Event rate (per 100 person years)**<br>(95% CI)	20<br>(9, 42)	42<br>(25, 69)		14<br>(5, 36)	43<br>(25, 73)		53<br>(17, 163)	36<br>(9, 145)	
**Unadjusted Hazard ratio**<br>(95% CI)<br>P-value			0.48<br>(0.20, 1.19)<br>0.11			0.32<br>(0.11, 0.99)<br>0.048			1.46<br>(0.24, 8.71)<br>0.68
**Adj. hazard ratio***<br>(95% CI)<br>P-value			0.48<br>(0.20, 1.18)<br>0.11						
**A/B x arm interaction+**	0.16								

*Adjusted for ODYSSEY A and B strata only in Total.
+Z-test
Note: Hazard ratios from Cox regression models presented as an alternative analysis to the comparison of proportion with clinical or virological failure (see table above)

S8.3 Comparison of proportion with clinical or virological failure by 96 weeks

	**Total**			**A**			**B**		
	**DTG**	**SOC**	**DTG vs. SOC**	**DTG**	**SOC**	**DTG vs. SOC**	**DTG**	**SOC**	**DTG vs. SOC**
**Participants randomised**	42	43		35	37		7	6	
**Total participants meeting primary endpoint**	12 (29%)	21 (49%)		9 (26%)	17 (46%)		3 (43%)	4 (67%)	
Insufficient virological response	0 (0%)	0 (0%)		0 (0%)	0 (0%)		0 (0%)	0 (0%)	
Confirmed VL>=400 copies/mL	9 (21%)	16 (37%)		7 (20%)	13 (35%)		2 (29%)	3 (50%)	
Severe WHO 3	0 (0%)	0 (0%)		0 (0%)	0 (0%)		0 (0%)	0 (0%)	
WHO 4	1 (2%)	1 (2%)		0 (0%)	1 (3%)		1 (14%)	0 (0%)	
Death	2 (5%)	4 (9%)		2 (6%)	3 (8%)		0 (0%)	1 (17%)	
**Unadjusted estimated probability of failure**	0.31	0.48		0.27	0.46		0.48	0.60	
[95%CI]	[0.19, 0.48]	[0.35, 0.63]		[0.15, 0.47]	[0.32, 0.63]		[0.01, 1.00]	[0.17, 0.99]	
Difference (DTG-SOC)			-0.18			-0.19			-0.12
[95%CI]			[-0.37, 0.01]			[-0.41, 0.01]			[-0.61, 0.37]
P-value**			0.071			0.079			0.61
**Adjusted* estimated probability of failure**	0.31	0.48							
[95%CI]	[0.18, 0.48]	[0.35, 0.63]							
Difference (DTG-SOC)			-0.18						
[95%CI]			[-0.36, 0.02]						
P-value**			0.057						
**A/B x arm interaction+**	0.81					.			

*Adjusted for ODYSSEY A and B strata only in Total. Analysis conducted of primary endpoint in <14kg cohort at 96 weeks (see S3.2 statistical methods)
+Z-test

S8.4 Incidence of clinical or virological failure by 96 weeks (alternative approach to analysis of primary endpoint)

	Total			A			B		
	**DTG**	**SOC**	**DTG vs SOC**	**DTG**	**SOC**	**DTG vs SOC**	**DTG**	**SOC**	**DTG vs SOC**
**Participants randomised**	42	43		35	37		7	6	
**Total participants meeting primary endpoint**	12 (29%)	21 (49%)		9 (26%)	17 (46%)		3 (43%)	4 (67%)	
**Person years**	63	59		53	51		9	8	
**Event rate (per 100 person years)**<br>(95% CI)	19<br>(11, 34)	36<br>(23, 55)		17<br>(9, 32)	33<br>(21, 54)		32<br>(10, 99)	49<br>(18, 130)	
**Unadjusted Hazard ratio**<br>(95% CI)<br>P-value			0.55<br>(0.27, 1.13)<br>0.10			0.52<br>(0.23, 1.16)<br>0.11			0.70<br>(0.16, 3.15)<br>0.65
**Adj. hazard ratio***<br>(95% CI)<br>P-value			0.55<br>(0.27, 1.12)<br>0.10						
**A/B x arm interaction+**	0.74								

*Adjusted for ODYSSEY A and B strata only in Total.
+Z-test
Note: Hazard ratios from Cox regression models presented as an alternative analysis to the comparison of proportion with clinical or virological failure (see table above)

S8.5 Time to clinical or virological failure

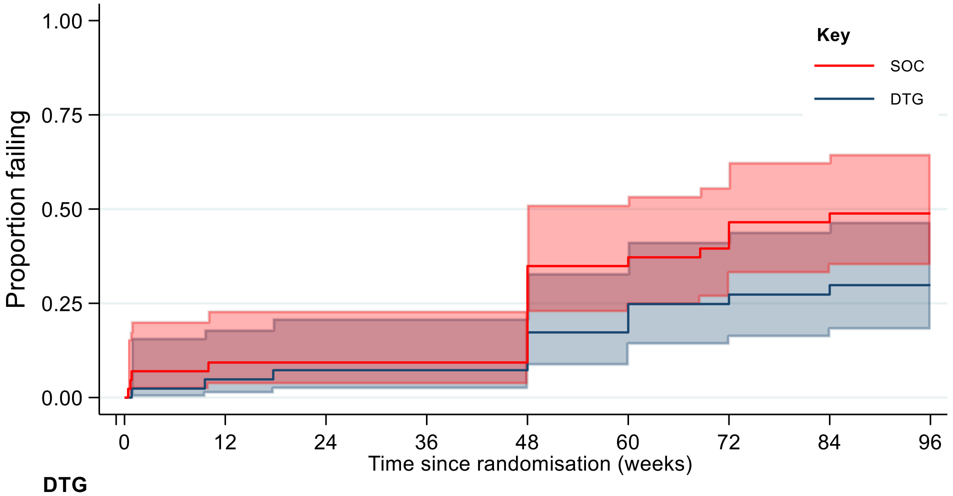

**DTG**									
At-risk (no event)	42	39	38	37	33	30	29	28	28
Censored	0	1	1	2	2	2	2	2	2
Event	0	2	3	3	7	10	11	12	12
**SOC**									
At-risk (no event)	43	39	39	39	28	27	23	22	22
Censored	0	0	0	0	0	0	0	0	0
Event	0	4	4	4	15	16	20	21	21

HR=0.55 (0.27, 1.12) p=0.10 log rank p=0.081

S8.6 Comparison of proportion with clinical or virological failure by 96 weeks

	**Analysis**	**Difference (DTG-SOC)**		**p-value***
Difference (DTG-SOC) [95%CI]	<14kg Bayesian Analysis	-0.102	[-0.187,-0.016]	0.020
	<14kgs	-0.177	[-0.356,0.023]	0.057
	≥14kgs	-0.080	[-0.142,-0.031]	0.0039
	Pooled Analysis	-0.092	[-0.144,-0.034]	0.0010
Test of Heterogeneity between above and below 14kgs	P-Value	0.32		

*bootstrapped p-values in <14kgs, ≥14kgs, and pooled analyses; z test for significance of the effect size in <14kg Bayesian analysis

S8.7 Comparison of proportion of participants with HIV-1 RNA <50c/ml at 48 weeks

		Total		A		B	
		**DTG**	**SOC**	**DTG**	**SOC**	**DTG**	**SOC**
**Participants randomised**		42	43	35	37	7	6
HIV-1 RNA <50c/mL at 48 weeks		15	19	12	18	3	1
HIV-1 RNA >=50c/mL at 48 weeks		19	20	15	15	4	5
**Unadjusted**	Proportion<50c/mL	44	49	44	55	43	17
	[95%Confidence Interval]	[28, 61]	[33, 65]	[27, 64]	[37, 71]	[10, 83]	[1, 77]
	Treatment Difference (DTG-SOC)	-5		-10		26	
	[95% Confidence Interval]	[-28, 18]		[-35, 15]		[-21, 73]	
	P-value+	0.69		0.44		0.31	
**Adjusted#**	Estimated Difference (DTG-SOC)	-4					
	[95% Confidence Interval]	[-26, 19]					
	P-value	0.76					

+P-value for total population and A/B stratum-specific p-values derived from chi-squared test

#Logistic regression adjusted for ODYSSEY A and B strata. Difference between arms in proportion is marginal risk difference from logistic regression model

S8.8 Comparison of proportion of participants with HIV-1 RNA <400c/ml at 48 weeks

		Total		A		B	
		**DTG**	**SOC**	**DTG**	**SOC**	**DTG**	**SOC**
**Participants randomised**		42	43	35	37	7	6
HIV-1 RNA <400c/mL at 48 weeks		25	27	21	23	4	4
HIV-1 RNA >=400c/mL at 48 weeks		9	12	6	10	3	2
	[95%Confidence Interval]	[56, 86]	[53, 82]	[57, 90]	[52, 83]	[17, 90]	[18, 95]
	Treatment Difference (DTG-SOC)	4		8		-10	
	[95% Confidence Interval]	[-16, 25]		[-14, 30]		[-62, 43]	
	P-value+	0.69		0.48		0.72	
**Adjusted#**	Estimated Difference (DTG-SOC)	5					
	[95% Confidence Interval]	[-16, 26]					
	P-value	0.64					

+P-value for total population and A/B stratum-specific p-values derived from chi-squared test

#Logistic regression adjusted for ODYSSEY A and B strata. Difference between arms in proportion is marginal risk difference from logistic regression model

S8.9 Comparison of proportion of participants with HIV-1 RNA <50c/ml at 96 weeks

		Total		A		B	
		**DTG**	**SOC**	**DTG**	**SOC**	**DTG**	**SOC**
**Participants randomised**		42	43	35	37	7	6
HIV-1 RNA <50c/mL at 96 weeks		27	19	23	18	4	1
HIV-1 RNA >=50c/mL at 96 weeks		8	17	5	13	3	4
	[95%Confidence Interval]	[60, 88]	[36, 69]	[63, 93]	[40, 74]	[17, 90]	[1, 85]
	Treatment Difference (DTG-SOC)	24		24		37	
	[95% Confidence Interval]	[3, 46]		[2, 47]		[-14, 88]	
	P-value+	0.032		0.045		0.20	
**Adjusted#**	Estimated Difference (DTG-SOC)	26					
	[95% Confidence Interval]	[6, 47]					
	P-value	0.021					

+P-value for total population and A/B stratum-specific p-values derived from chi-squared test

#Logistic regression adjusted for ODYSSEY A and B strata. Difference between arms in proportion is marginal risk difference from logistic regression model

S8.10 Comparison of proportion of participants with HIV-1 RNA <400 c/ml at 96 weeks

		Total		A		B	
		**DTG**	**SOC**	**DTG**	**SOC**	**DTG**	**SOC**
**Participants randomised**		42	43	35	37	7	6
HIV-1 RNA <400c/mL at 96 weeks		33	26	26	23	7	3
HIV-1 RNA >=400c/mL at 96 weeks		3	10	3	8	0	2
**Unadjusted**	Proportion<400c/mL	92	72	90	74	100	60
	[95%Confidence Interval]	[76, 97]	[55, 85]	[71, 97]	[55, 87]	[., .]	[11, 95]
	Treatment Difference (DTG-SOC)	19		15		40	
	[95% Confidence Interval]	[2, 37]		[-4, 34]		[-3, 83]	
	P-value+	0.032		0.12		0.067	
**Adjusted#**	Estimated Difference (DTG-SOC)	19					
	[95% Confidence Interval]	[2, 37]					
	P-value	0.038					

+P-value for total population and A/B stratum-specific p-values derived from chi-squared test

#Logistic regression adjusted for ODYSSEY A and B strata. Difference between arms in proportion is marginal risk difference from logistic regression model

S8.11 FDA snapshot algorithm - comparison of proportion of participants with HIV-1 RNA <50c/ml at 48 weeks

		Total		A		B	
		**DTG**	**SOC**	**DTG**	**SOC**	**DTG**	**SOC**
**Participants randomised**		42	43	35	37	7	6
**HIV-RNA<50 c/ml (Total participants)**		15	19	12	18	3	1
	Proportion	36%	44%	34%	49%	43%	17%
	Treatment difference (DTG-SOC)	-8		-14		26	
	[95% Confidence Interval]	[-29, 12]		[-37, 8]		[-21, 73]	
	Unadjusted P-value+	0.43		0.22		0.31	
	Adjusted p-value#	0.44					
**HIV-RNA>=50 c/ml (Total participants)**		19	20	15	15	4	5
	Proportion	45%	47%	43%	41%	57%	83%
	Treatment difference (DTG-SOC)	-1		2		-26	
	[95% Confidence Interval]	[-22, 20]		[-20, 25]		[-73, 21]	
	Unadjusted P-value+	0.91		0.84		0.31	
	Adjusted p-value#	0.85					
Components of HIV-RNA>=50 c/ml							
	HIV-RNA >=50 c/ml - in window	18	20	14	15	4	5
	VL>=50 c/ml prior to permitted switch or LTFU	1	0	1	0	0	0
**Missing VL in week 48 window (Total participants)**		8	4	8	4	0	0
	Proportion	19%	9%	23%	11%	0%	0%
	VL<50 c/ml prior to permitted switch or LTFU	1	0	1	0	0	0
	On non-permitted ART due to toxicity or died	2	3	2	3	0	0
	Initial regimen but no VL in window	5	1	5	1	0	0

+P-value for total population and A/B stratum-specific p-values derived from chi-squared test
#Logistic regression p-value adjusted for ODYSSEY A and B strata

S8.12 FDA snapshot algorithm - comparison of proportion of participants with HIV-1 RNA <400 c/ml at 48 weeks

		Total		A		B	
		**DTG**	**SOC**	**DTG**	**SOC**	**DTG**	**SOC**
**Participants randomised**		42	43	35	37	7	6
**HIV-RNA<400 c/ml (Total participants)**		25	27	21	23	4	4
	Proportion	60%	63%	60%	62%	57%	67%
	Treatment difference (DTG-SOC)	-3		-2		-10	
	[95% Confidence Interval]	[-24, 17]		[-25, 20]		[-62, 43]	
	Unadjusted P-value+	0.76		0.85		0.72	
	Adjusted p-value#	.76					
**HIV-RNA>=400 c/ml (Total participants)**		9	12	6	10	3	2
	Proportion	21%	28%	17%	27%	43%	33%
	Treatment difference (DTG-SOC)	-6		-10		10	
	[95% Confidence Interval]	[-25, 12]		[-29, 9]		[-43, 62]	
	Unadjusted P-value+	0.49		0.31		0.72	
	Adjusted p-value#	0.46					
Components of HIV-RNA>=400 c/ml							
	HIV-RNA >=400 c/ml - in window	8	12	5	10	3	2
	VL>=400 c/ml prior to permitted switch or LTFU	1	0	1	0	0	0
**Missing VL in week 48 window (Total participants)**		8	4	8	4	0	0
	Proportion	19%	9%	23%	11%	0%	0%
	VL<400 c/ml prior to permitted switch or LTFU	1	0	1	0	0	0
	On non-permitted ART due to toxicity or died	2	3	2	3	0	0
	Initial regimen but no VL in window	5	1	5	1	0	0

+P-value for total population and A/B stratum-specific p-values derived from chi-squared test
#Logistic regression p-value adjusted for ODYSSEY A and B strata

S8.13 FDA snapshot algorithm - comparison of proportion of participants with HIV-1 RNA <50 c/ml at 96 weeks

		Total		A		B	
		**DTG**	**SOC**	**DTG**	**SOC**	**DTG**	**SOC**
**Participants randomised**		42	43	35	37	7	6
**HIV-RNA<50 c/ml (Total participants)**		27	18	23	17	4	1
	Proportion	64%	42%	66%	46%	57%	17%
	Treatment difference (DTG-SOC)	22		20		40	
	[95% Confidence Interval]	[2, 43]		[-3, 42]		[-7, 88]	
	Unadjusted P-value+	0.038		0.092		0.13	
	Adjusted p-value#	0.035					
**HIV-RNA>=50 c/ml (Total participants)**		8	18	6	14	2	4
	Proportion	19%	42%	17%	38%	29%	67%
	Treatment difference (DTG-SOC)	-23		-21		-38	
	[95% Confidence Interval]	[-42, -4]		[-41, -1]		[-89, 12]	
	Unadjusted P-value+	0.022		0.050		0.17	
	Adjusted p-value#	0.021					
Components of HIV-RNA>=50 c/ml							
	HIV-RNA >=50 c/ml - in window	7	12	5	9	2	3
	Non-permitted ART (other than toxicity)	0	5	0	4	0	1
	VL>=50 c/ml prior to permitted switch or LTFU	1	1	1	1	0	0
**Missing VL in week 96 window (Total participants)**		7	7	6	6	1	1
	Proportion	17%	16%	17%	16%	14%	17%
	VL<50 c/ml prior to permitted switch or LTFU	2	0	2	0	0	0
	On non-permitted ART due to toxicity or died	2	4	2	3	0	1
	Initial regimen but no VL in window	3	3	2	3	1	0

+P-value for total population and A/B stratum-specific p-values derived from chi-squared test

#Logistic regression p-value adjusted for ODYSSEY A and B strata

S8.14 FDA snapshot algorithm - comparison of proportion of participants with HIV-1 RNA <400 c/ml at 96 weeks

		Total		A		B	
		**DTG**	**SOC**	**DTG**	**SOC**	**DTG**	**SOC**
**Participants randomised**		42	43	35	37	7	6
**HIV-RNA<400 c/ml (Total participants)**		32	22	26	20	6	2
	Proportion	76%	51%	74%	54%	86%	33%
	Treatment difference (DTG-SOC)	25		20		52	
	[95% Confidence Interval]	[5, 45]		[-1, 42]		[7, 98]	
	Unadjusted P-value+	.017		.074		.053	
	Adjusted p-value#	.018					
**HIV-RNA>=400 c/ml (Total participants)**		4	14	4	11	0	3
	Proportion	10%	33%	11%	30%	0%	50%
	Treatment difference (DTG-SOC)	-23		-18		-50	
	[95% Confidence Interval]	[-40, -6]		[-36, -0]		[-90, -10]	
	Unadjusted P-value+	0.0094		0.056		0.033	
	Adjusted p-value#	0.013					
Components of HIV-RNA>=400 c/ml							
	HIV-RNA >=400 c/ml - in window	3	8	3	6	0	2
	Non-permitted ART (other than toxicity)	0	5	0	4	0	1
	VL>=400 c/ml prior to permitted switch or LTFU	1	1	1	1	0	0
**Missing VL in week 96 window (Total participants)**		6	7	5	6	1	1
	Proportion	14%	16%	14%	16%	14%	17%
	VL<400 c/ml prior to permitted switch or LTFU	2	0	2	0	0	0
	On non-permitted ART due to toxicity or died	2	4	2	3	0	1
	Initial regimen but no VL in window	2	3	1	3	1	0

+P-value for total population and A/B stratum-specific p-values derived from chi-squared test
#Logistic regression p-value adjusted for ODYSSEY A and B strata

S8.15 Per-protocol analysis

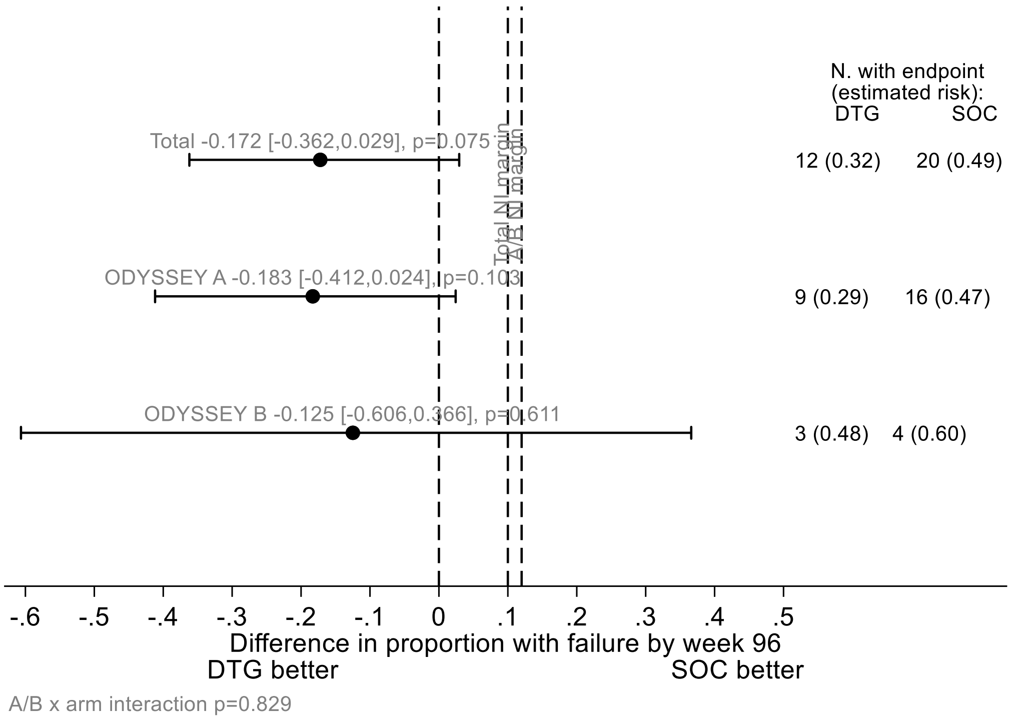

Note: 1 participant was censored prior to meeting the primary endpoint due to ART discontinuation for >31days.

S8.16 Total population - Subgroup analyses for primary endpoint

	DTG				SOC								
	**Participants randomised**	**Total participants meeting primary endpoint**	**Person-years**	**Event rate (95% CI)**	**Participants randomised**	**Total participants meeting primary endpoint**	**Person-years**	**Event rate (95% CI)**	**Hazard ratio (95% CI)**	**p-value**	**Adjusted hazard ratio (95% CI)**	**p-value**	**p-value (interaction)**
**Age at baseline**													
<1 year	16	4 (25.0%)	26	15.2 (5.7, 40.5)	16	11 (68.8%)	18	62.2 (34.4, 112.3)	0.26 (0.08, 0.82)	0.022	0.26 (0.08, 0.82)	0.021	0.11
>=1 year	26	8 (30.8%)	36	22.0 (11.0, 43.9)	27	10 (37.0%)	41	24.2 (13.0, 44.9)	0.92 (0.36, 2.32)	0.85	0.87 (0.34, 2.22)	0.77	

S8.17 ODYSSEY A - Subgroup analyses for primary endpoint

	DTG				SOC						
	**Participants randomised**	**Total participants meeting primary endpoint**	**Person-years**	**Event rate (95% CI)**	**Participants randomised**	**Total participants meeting primary endpoint**	**Person-years**	**Event rate (95% CI)**	**Hazard ratio (95% CI)**	**p-value**	**p-value (interaction)**
**Age at baseline**											
<1 year	16	4 (25.0%)	26	15.2 (5.7, 40.5)	16	11 (68.8%)	18	62.2 (34.4, 112.3)	0.26 (0.08, 0.81)	0.021	0.10
>=1 year	19	5 (26.3%)	27	18.5 (7.7, 44.5)	21	6 (28.6%)	33	18.1 (8.1, 40.3)	1.02 (0.31, 3.34)	0.98	

Please note no participants in ODYSSEY B were <1 year at baseline.

S8.18 Cumulative probability of suppression by treatment group (suppression defined as two consecutive viral loads <200cp/ml)

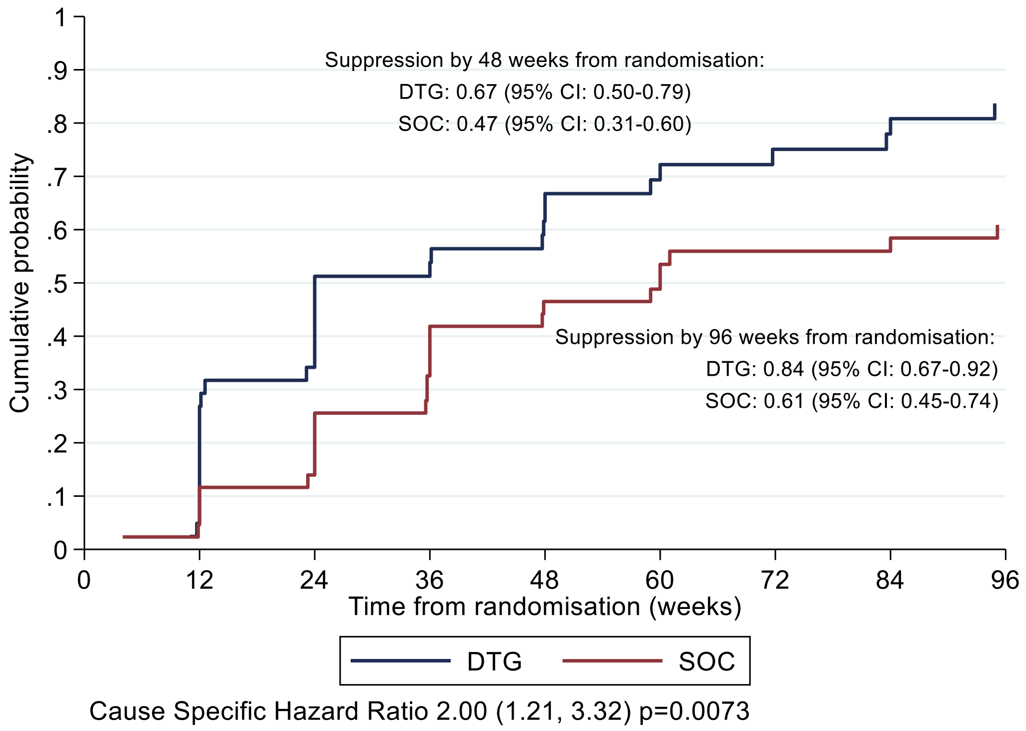

Suppression defined as two consecutive viral loads <200c/mL. Treating switch from initial trial regimen or switch in backbone for failure and death as competing risks

S8.19 Cumulative probability of suppression by treatment group (suppression defined as two consecutive viral loads <400cp/ml)

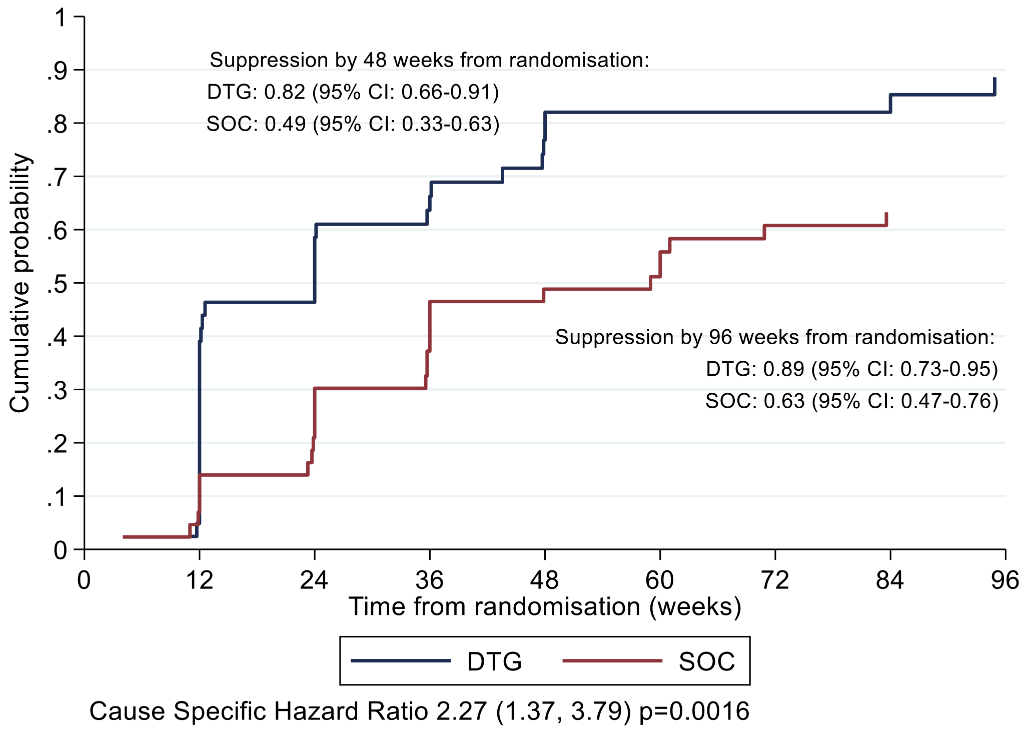

Suppression defined as two consecutive viral loads <400c/mL. Treating switch from initial trial regimen or switch in backbone for failure and death as competing risks

S8.20 Rate of clinical events to trial censoring date: WHO 4, severe WHO 3 and death

	Total			A			B		
	**DTG**	**SOC**	**Total**	**DTG**	**SOC**	**Total**	**DTG**	**SOC**	**Total**
**Participants randomised and included**	42	43	85	35	37	72	7	6	13
Person years	93	95	188	76	81	156	18	14	32
**Total number of events [Number of participants]**	3 [3]	6 [6]	9 [9]	2 [2]	5 [5]	7 [7]	1 [1]	1 [1]	2 [2]
Severe WHO 3	0 [0]	0 [0]	0 [0]	0 [0]	0 [0]	0 [0]	0 [0]	0 [0]	0 [0]
WHO 4	1 [1]	2 [2]	3 [3]	0 [0]	2 [2]	2 [2]	1 [1]	0 [0]	1 [1]
Death	2 [2]	4 [4]	6 [6]	2 [2]	3 [3]	5 [5]	0 [0]	1 [1]	1 [1]
**Number of events [Number of children]**									
**Infectious Disease**	0 [0]	5 [5]	5 [5]	0 [0]	4 [4]	4 [4]	0 [0]	1 [1]	1 [1]
Gastroenteritis *	0 [0]	1 [1]	1 [1]	0 [0]	0 [0]	0 [0]	0 [0]	1 [1]	1 [1]
Pneumonia no organism identified, aspiration pneumonia *	0 [0]	1 [1]	1 [1]	0 [0]	1 [1]	1 [1]	0 [0]	0 [0]	0 [0]
Tuberculosis - abdominal	0 [0]	1 [1]	1 [1]	0 [0]	1 [1]	1 [1]	0 [0]	0 [0]	0 [0]
Tuberculosis - disseminated/miliary	0 [0]	1 [1]	1 [1]	0 [0]	1 [1]	1 [1]	0 [0]	0 [0]	0 [0]
Tuberculosis - pulmonary - smear negative or not done+Severe malnutrition *	0 [0]	1 [1]	1 [1]	0 [0]	1 [1]	1 [1]	0 [0]	0 [0]	0 [0]
**Non HIV related deaths**	1 [1]	0 [0]	1 [1]	1 [1]	0 [0]	1 [1]	0 [0]	0 [0]	0 [0]
Traumatic *	1 [1]	0 [0]	1 [1]	1 [1]	0 [0]	1 [1]	0 [0]	0 [0]	0 [0]
**Systemic**	2 [2]	1 [1]	3 [3]	1 [1]	1 [1]	2 [2]	1 [1]	0 [0]	1 [1]
Chest infection+Severe malnutrition	1 [1]	0 [0]	1 [1]	0 [0]	0 [0]	0 [0]	1 [1]	0 [0]	1 [1]
Kwashiorkor *	1 [1]	0 [0]	1 [1]	1 [1]	0 [0]	1 [1]	0 [0]	0 [0]	0 [0]
Severe malnutrition *	0 [0]	1 [1]	1 [1]	0 [0]	1 [1]	1 [1]	0 [0]	0 [0]	0 [0]
**Event rate (all events)**									
Event rate (per 100 person years)	3.2	6.3	4.8	2.6	6.2	4.5	5.6	7.1	6.3
(95% CI)	(1.0, 10.0)	(2.8, 14.1)	(2.5, 9.2)	(0.7, 10.6)	(2.6, 14.9)	(2.1, 9.4)	(0.8, 40.0)	(1.0, 50.2)	(1.6, 25.1)
Unadjusted Rate ratio	0.51	1 (ref)		0.43	1 (ref)		0.80	1 (ref)	
(95% CI)	(0.12, 2.07)	-		(0.08, 2.32)	-		(0.05, 12.27)		
P-value			0.34			0.32			0.87
Adj. Rate ratio+	0.50	1 (ref)							
(95% CI)	(0.12, 2.01)								
P-value			0.33						
**Time to first event (WHO 4, severe WHO 3 or death)**									
Unadjusted Hazard ratio	0.50	1 (ref)		0.41	1 (ref)		0.93	1 (ref)	
(95% CI)	(0.13, 2.02)	-		(0.08, 2.11)	-		(0.06, 14.83)		
P-value			0.33			0.29			0.96
Adj. hazard ratio+	0.50	1 (ref)							
(95% CI)	(0.13, 2.00)								
P-value			0.33						
A/B x arm interaction	0.6245								

*Resulted in death
+Adjusted for ODYSSEY A and B strata in total.

S8.21 Rate of clinical events to week 96: WHO 4, severe WHO 3 and death

	Total			A			B		
	**DTG**	**SOC**	**Total**	**DTG**	**SOC**	**Total**	**DTG**	**SOC**	**Total**
**Participants randomised and included**	42	43	85	35	37	72	7	6	13
Person years	70	73	143	57	62	120	13	11	23
**Total number of events [Number of participants]**	3 [3]	6 [6]	9 [9]	2 [2]	5 [5]	7 [7]	1 [1]	1 [1]	2 [2]
Severe WHO 3	0 [0]	0 [0]	0 [0]	0 [0]	0 [0]	0 [0]	0 [0]	0 [0]	0 [0]
WHO 4	1 [1]	2 [2]	3 [3]	0 [0]	2 [2]	2 [2]	1 [1]	0 [0]	1 [1]
Death	2 [2]	4 [4]	6 [6]	2 [2]	3 [3]	5 [5]	0 [0]	1 [1]	1 [1]
**Number of events [Number of children]**									
**Infectious Disease**	0 [0]	5 [5]	5 [5]	0 [0]	4 [4]	4 [4]	0 [0]	1 [1]	1 [1]
Gastroenteritis *	0 [0]	1 [1]	1 [1]	0 [0]	0 [0]	0 [0]	0 [0]	1 [1]	1 [1]
Pneumonia no organism identified, aspiration pneumonia *	0 [0]	1 [1]	1 [1]	0 [0]	1 [1]	1 [1]	0 [0]	0 [0]	0 [0]
Tuberculosis - abdominal	0 [0]	1 [1]	1 [1]	0 [0]	1 [1]	1 [1]	0 [0]	0 [0]	0 [0]
Tuberculosis - disseminated/miliary	0 [0]	1 [1]	1 [1]	0 [0]	1 [1]	1 [1]	0 [0]	0 [0]	0 [0]
Tuberculosis - pulmonary - smear negative or not done+Severe malnutrition *	0 [0]	1 [1]	1 [1]	0 [0]	1 [1]	1 [1]	0 [0]	0 [0]	0 [0]
**Non HIV related deaths**	1 [1]	0 [0]	1 [1]	1 [1]	0 [0]	1 [1]	0 [0]	0 [0]	0 [0]
Traumatic *	1 [1]	0 [0]	1 [1]	1 [1]	0 [0]	1 [1]	0 [0]	0 [0]	0 [0]
**Systemic**	2 [2]	1 [1]	3 [3]	1 [1]	1 [1]	2 [2]	1 [1]	0 [0]	1 [1]
Chest infection+Severe malnutrition	1 [1]	0 [0]	1 [1]	0 [0]	0 [0]	0 [0]	1 [1]	0 [0]	1 [1]
Kwashiorkor *	1 [1]	0 [0]	1 [1]	1 [1]	0 [0]	1 [1]	0 [0]	0 [0]	0 [0]
Severe malnutrition *	0 [0]	1 [1]	1 [1]	0 [0]	1 [1]	1 [1]	0 [0]	0 [0]	0 [0]
**Event rate (all events)**									
Event rate (per 100 person years)	4.3	8.3	6.3	3.5	8.1	5.9	7.8	9.5	8.5
(95% CI)	(1.4, 13.2)	(3.7, 18.4)	(3.3, 12.1)	(0.9, 13.9)	(3.4, 19.3)	(2.8, 12.3)	(1.1, 55.1)	(1.3, 67.5)	(2.1, 34.2)
Unadjusted Rate ratio	0.52	1 (ref)		0.43	1 (ref)		0.82	1 (ref)	
(95% CI)	(0.13, 2.10)	-		(0.08, 2.33)	-		(0.05, 12.37)		
P-value			0.36			0.33			0.88
Adj. Rate ratio+	0.51	1 (ref)							
(95% CI)	(0.13, 2.03)								
P-value			0.34						
**Time to first event (WHO 4, severe WHO 3 or death)**									
Unadjusted Hazard ratio	0.50	1 (ref)		0.41	1 (ref)		0.93	1 (ref)	
(95% CI)	(0.13, 2.02)	-		(0.08, 2.11)	-		(0.06, 14.83)		
P-value			0.33			0.29			0.96
Adj. hazard ratio+	0.50	1 (ref)							
(95% CI)	(0.13, 2.00)								
P-value			0.33						
A/B x arm interaction	0.6245								

*Resulted in death

+Adjusted for ODYSSEY A and B strata in total. Unadjusted in A and B comparison therefore identical to the unadjusted

S8.22 Details of WHO 3 (Severe) or 4 events or deaths during follow-up

ID	A/B	Treatment arm	ART regimen at event	Grade	Age (Weeks)	Event Week	Event	Type
1	A	DTG	ABC 3TC DTG	4	67	0 [6days]	Kwashiorkor	Death
2	A	DTG	ABC 3TC DTG	4	34	17	Traumatic	Death
3	B	DTG	ZDV 3TC DTG	4	162	9	Chest infection+Severe malnutrition	WHO4
4	A	SOC	ABC 3TC EFV	3	257	53	Tuberculosis - abdominal	WHO4
5	A	SOC	ABC 3TC LOP	4	28	0 [5days]	Pneumonia no organism identified, aspiration pneumonia	Death
6	A	SOC	ABC 3TC LOP	2	7	0 [3days]	Tuberculosis - disseminated/miliary	WHO4
7	A	SOC	ABC 3TC LOP	4	64	0 [6days]	Severe malnutrition	Death
8	A	SOC	ABC 3TC NVP	4	37	10	Tuberculosis - pulmonary - smear negative or not done+Severe malnutrition	Death
9	B	SOC	ABC 3TC LOP	4	219	68	Gastroenteritis	Death

ID 1, 7, and 8 had their WHO 4 condition (kwashiorkor, severe malnutrition and severe malnutrition respectively) pre-exisiting prior to randomisation, therefore the event in follow-up is death

S8.23 Genotypic resistance comparing dolutegravir-based ART versus standard of care#

	ODYSSEY A				ODYSSEY B			
	DTG		SOC		DTG		SOC	
**Participants with virological failure by 96 weeks^**	7	(20%)	13	(35%)	2	(29%)	3	(50%)
**Participants with resistance post-failure ʅ**								
Any	6/6	(100%)	12/12	(100%)	2/2	(100%)	3/3	(100%)
NRTI	1/6	(17%)	10/12	(83%)	1/2	(50%)	3/3	(100%)
NNRTI	6/6	(100%)	11/12	(92%)	2/2	(100%)	3/3	(100%)
PI	0/6	(0%)	2/12	(17%)	0/2	(0%)	0/3	(0%)
INSTI	0/5	(0%)	-	-	1/2**	(50%)	-	-

ʅ Major IAS-USA drug resistance mutations defined according to 2019 update of the IAS-USA drug resistance mutations.[19] Percentage with resistance post-failure, of those with virological failure by week 96 and post-failure resistance test available for drug-class ('-' indicates integrase gene not sequenced for SOC arm).

**1 participant with INSTI resistance developed Asn155Asn/His mutation

^25 participants met a virological endpoint by week 96 (confirmed viral load≥400 c/mL after week 36 [n=25] or lack of virological response by week 24 followed by ART switch [n=0])

#Note: due to low availability of baseline resistance tests, proportion with emergent drug resistance could not be estimated

DTG, dolutegravir; **SOC**, standard of care; **NRTI**, Nucleoside reverse transcriptase inhibitors; **NNRTI**, Non-nucleoside reverse transcriptase inhibitors; **PI**, protease inhibitor; **INSTI**, Integrase Strand Transfer Inhibitor; **IAS-USA**, International AIDS Society-USA; **c/ml**, copies per millilitre; **ART**, antiretroviral therapy

S8.24 Resistance mutations identified post-failure in participants*

	DTG	SOC
**ODYSSEY A**		
**NRTI**		
Any NRTI	1/6	10/12
M41	0	1
K65	0	1
D67	1	1
K70	1	0
L74	0	2
Y115	0	1
M184	1	9
L210	0	1
T215	0	1
K219	1	2
**NNRTI**		
Any NNRTI	6/6	11/12
K101	1	1
K103	5	8
V106	1	1
E138	0	1
Y181	2	4
Y188	0	2
G190	0	3
H221	1	2
P225	1	1
**PI**		
Any PI	0/6	2/12
M46	0	1
I47	0	1
Q58	0	1
I84	0	1
**INSTI**		
Any INSTI	0/5	-
**ODYSSEY B**		
**NRTI**		
Any NRTI	1/2	3/3
D67	1	0
K70	1	0
M184	1	3
K219	1	0
**NNRTI**		
Any NNRTI	2/2	3/3
K101	0	1
K103	1	3
V106	1	0
E138	1	0
Y181	1	0
G190	0	1
**PI**		
Any PI	0/2	0/3
**INSTI**		
Any INSTI**	1/2	-

N155	1	-

*Reported for participants for whom a resistance test was available post-failure.

**1 participant with INSTI resistance: 1 N155NH.

Each participant may have one or more mutation to one or more drug class.

- indicates gene not sequenced.

S8.25 Treatment emergent resistance mutations post-failure in participants exposed to drug-class*

	DTG	SOC
**ODYSSEY A**		
**NRTI**		
Any NRTI	-	5/5
L74	-	1
M184	-	3
L210	-	1
K219	-	1
**NNRTI**		
Any NNRTI	-	-
**PI**		
Any PI	-	1/1
I47	-	1
**INSTI**		
Any INSTI	0/5	-
**ODYSSEY B**		
**NRTI**		
Any NRTI	1/1	-
D67	1	-
K70	1	-
**NNRTI**		
Any NNRTI	-	-
**PI**		
Any PI	-	-
**INSTI**		
Any INSTI**	1/2	-
N155	1	-

*Reported for participants for whom a resistance test was available post-failure and at baseline and exposed to drug-class during trial. Note: IN gene was only sequenced during follow-up (not at baseline due to lack of prior INSTI exposure) and therefore INSTI resistance identified post-failure is classified as emergent.

**1 participant with INSTI resistance: 1 N155NH.

Each participant may have one or more mutation to one or more drug class.

- indicates no participants with virological failure, exposed to drug-class, and gene sequenced at baseline and post-failure.

S9 Immunology

Note: ODYSSEY B is not presented here due to having few participants

Throughout this section, n refers to the number of participants with available measurement at each visit week. Numbers in models may be lower where there are missing data at baseline.

S9.1 Total population - Changes in CD4 count over follow-up

Weeks since randomisation	DTG			SOC			Unadjusted Difference*				Adjusted Difference**				Interaction++
	n	mean	(SE)	n	mean	(SE)	mean	(SE)	[95% CI]	p	mean	(SE)	[95% CI]	p	p
4	36	334	(104)	36	235	(105)	99	(148)	[-197, 395]	0.51	109	(145)	[-180, 398]	0.45	0.95
12	36	269	(134)	39	198	(132)	71	(190)	[-307, 450]	0.71	72	(190)	[-307, 452]	0.70	0.69
24	36	283	(116)	37	133	(114)	150	(163)	[-176, 475]	0.36	158	(163)	[-167, 483]	0.34	1
48	32	368	(152)	38	239	(141)	129	(208)	[-287, 545]	0.54	165	(202)	[-238, 567]	0.42	0.78
72	34	106	(128)	34	32	(126)	74	(180)	[-285, 433]	0.68	78	(182)	[-285, 441]	0.67	0.94
96	36	72	(116)	35	51	(118)	21	(167)	[-313, 355]	0.90	30	(169)	[-308, 368]	0.86	0.48
Average treatment differences through follow-up+							114	(119)	[-119, 346]	0.34	122	(116)	[-106, 350]	0.29	0.95

S9.2 ODYSSEY A - changes in CD4 count over follow-up

Weeks since randomisation	DTG			SOC			Unadjusted Difference*			
	n	mean	(SE)	n	mean	(SE)	mean	(SE)	[95% CI]	p
4	29	406	(120)	30	312	(119)	94	(170)	[-247 434]	0.58
12	30	319	(158)	33	225	(156)	94	(224)	[-355 542]	0.68
24	29	331	(136)	31	177	(131)	154	(189)	[-226 534]	0.42
48	25	516	(179)	32	331	(160)	186	(242)	[-299 670]	0.45
72	27	122	(157)	29	47	(149)	75	(216)	[-359 510]	0.73
96	29	58	(133)	31	97	(129)	-39	(187)	[-415 336]	0.83
Average treatment differences through follow-up+							116	(135)	[-148, 379]	0.39

*Change calculated using normal regression adjusting for baseline only. Presenting mean change from a baseline of 1549.8 in total and 1546.6 in A.

**Normal regression adjusting for baseline and ODYSSEY A/B strata.

+Linear mixed models fitted with random intercept and fixed effects for treatment group, study visit and adjustment covariates (baseline CD4 count and ODYSSEY A/B strata (total population only))

++ Test of heterogeneity of treatment effect between ODYSSEY A/B

S9.3 Total population - Changes in CD4 count over follow-up

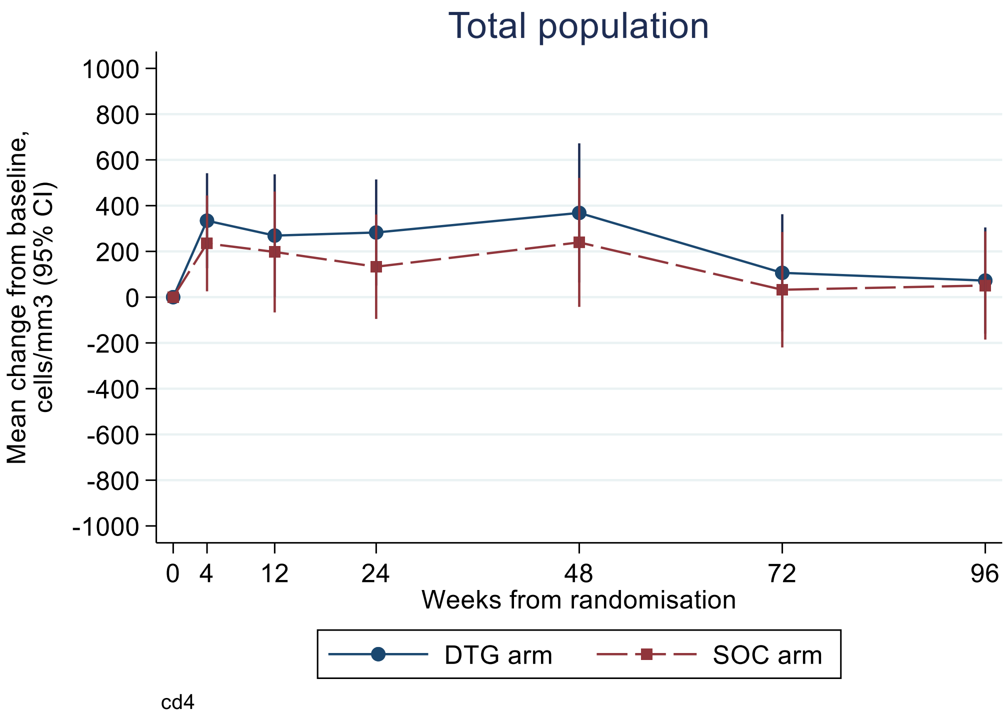

S9.4 ODYSSEY A - changes in CD4 count over follow-up

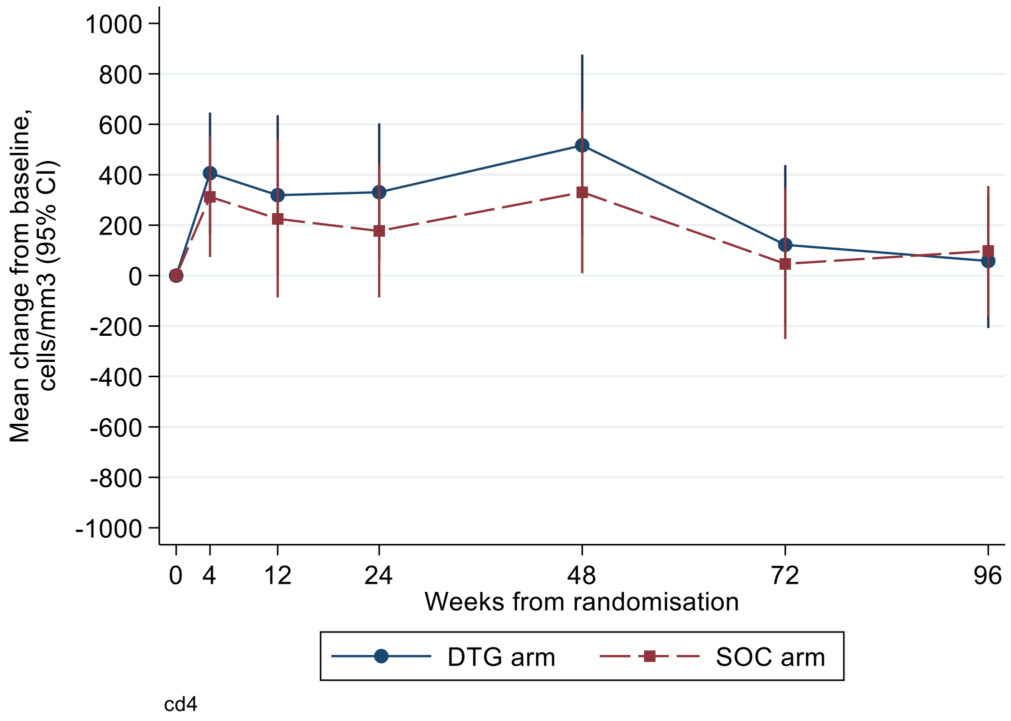

S9.5 Total population - Changes in CD4 percentage over follow-up

Weeks since randomisation	DTG			SOC			Unadjusted Difference*				Adjusted Difference**				Interaction++
	n	mean	(SE)	n	mean	(SE)	mean	(SE)	[95% CI]	p	mean	(SE)	[95% CI]	p	p
4	36	3.3	(1.4)	36	3.1	(1.4)	0.2	(2.0)	[-3.8, 4.2]	0.91	0.3	(2.0)	[-3.7, 4.2]	0.90	0.95
12	36	3.2	(1.4)	39	3.0	(1.4)	0.2	(2.0)	[-3.7, 4.2]	0.92	0.2	(2.0)	[-3.8, 4.2]	0.92	0.43
24	36	5.6	(1.3)	37	3.8	(1.3)	1.8	(1.9)	[-1.9, 5.5]	0.33	1.8	(1.9)	[-1.9, 5.5]	0.34	0.68
48	32	8.3	(1.6)	38	4.3	(1.5)	4.0	(2.2)	[-0.4, 8.4]	0.074	4.1	(2.2)	[-0.3, 8.5]	0.065	0.88
72	34	9.2	(1.7)	34	5.6	(1.6)	3.6	(2.3)	[-1.0, 8.2]	0.12	3.7	(2.3)	[-0.9, 8.4]	0.11	0.93
96	36	13.2	(1.6)	35	8.5	(1.7)	4.8	(2.3)	[0.1, 9.4]	0.047	4.7	(2.4)	[-0.1, 9.4]	0.053	0.37
Average treatment differences through follow-up+							2.7	(1.6)	[-0.3, 5.8]	0.082	2.7	(1.6)	[-0.3, 5.8]	0.080	0.71

S9.6 ODYSSEY A - changes in CD4 percentage over follow-up

Weeks since randomisation	DTG			SOC			Unadjusted Difference*			
	n	mean	(SE)	n	mean	(SE)	mean	(SE)	[95% CI]	p
4	29	3.8	(1.7)	30	3.4	(1.7)	0.4	(2.4)	[-4.4 5.3]	0.86
12	30	2.8	(1.7)	33	3.3	(1.6)	-0.5	(2.4)	[-5.2 4.2]	0.84
24	29	5.2	(1.5)	31	3.8	(1.5)	1.5	(2.1)	[-2.8 5.7]	0.49
48	25	9.0	(2.0)	32	4.9	(1.7)	4.1	(2.6)	[-1.2 9.4]	0.12
72	27	9.8	(2.1)	29	6.0	(1.9)	3.8	(2.8)	[-1.8 9.5]	0.18
96	29	12.7	(2.0)	31	9.0	(1.9)	3.7	(2.8)	[-2.0 9.3]	0.20
Average treatment differences through follow-up+							2.5	(1.9)	[-1.1, 6.1]	0.18

*Change calculated using normal regression adjusting for baseline only. Presenting mean change from a baseline of 23.6 in total and 23.1 in A.

**Normal regression adjusting for baseline and ODYSSEY A/B strata.

+Linear mixed models fitted with random intercept and fixed effects for treatment group, study visit and adjustment covariates (baseline CD4 percentage and ODYSSEY A/B strata (total population only))

++ Test of heterogeneity of treatment effect between ODYSSEY A/B

S9.7 Total population - Changes in CD4 percentage over follow-up

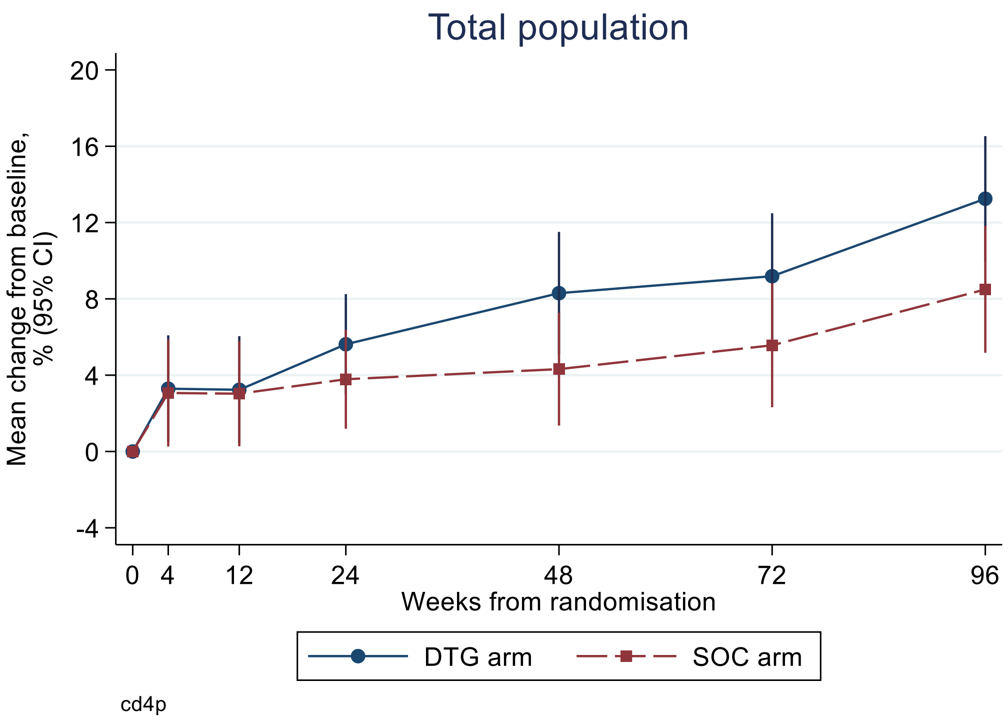

S9.8 ODYSSEY A - changes in CD4 percentage over follow-up

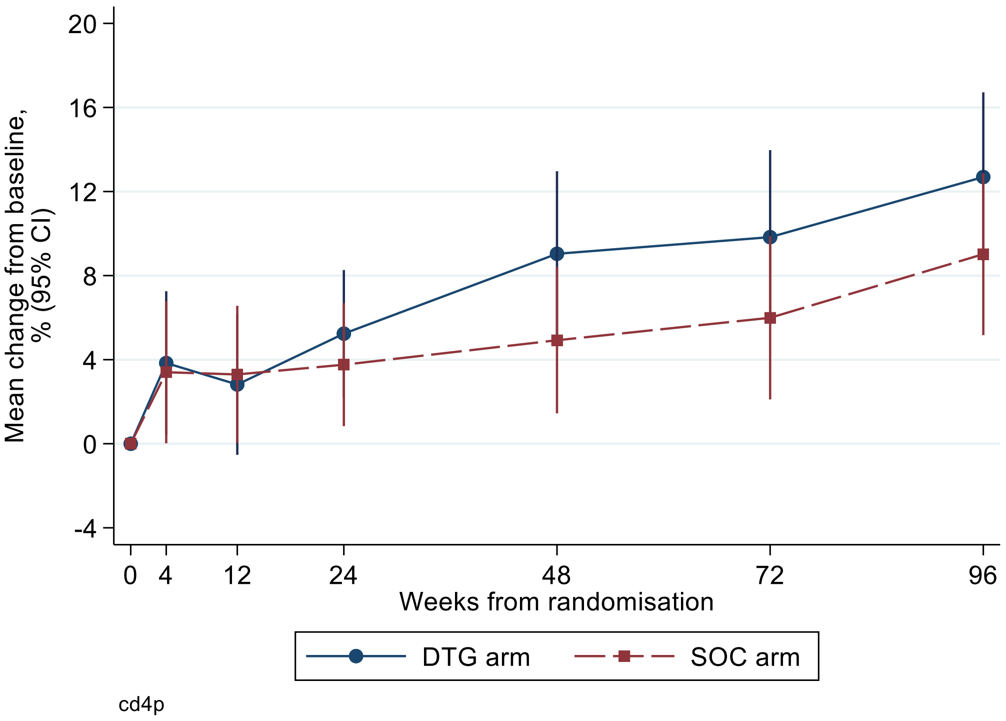

S9.9 Total population - Changes in CD4/CD8 ratio over follow-up

Weeks since randomisation	DTG			SOC			Unadjusted Difference*				Adjusted Difference**				Interaction++
	n	mean	(SE)	n	mean	(SE)	mean	(SE)	[95% CI]	p	mean	(SE)	[95% CI]	p	p
4	36	0.17	(0.08)	36	0.30	(0.08)	-0.13	(0.12)	[-0.36, 0.10]	0.26	-0.13	(0.11)	[-0.36, 0.10]	0.27	0.43
12	36	0.12	(0.06)	39	0.15	(0.06)	-0.03	(0.09)	[-0.20, 0.15]	0.75	-0.03	(0.09)	[-0.21, 0.15]	0.75	0.28
24	36	0.37	(0.08)	37	0.20	(0.07)	0.17	(0.11)	[-0.05, 0.38]	0.12	0.17	(0.11)	[-0.05, 0.38]	0.12	0.64
48	32	0.46	(0.08)	38	0.26	(0.08)	0.19	(0.11)	[-0.03, 0.42]	0.090	0.21	(0.11)	[-0.01, 0.43]	0.066	0.56
72	34	0.49	(0.09)	34	0.26	(0.09)	0.24	(0.12)	[-0.01, 0.49]	0.062	0.25	(0.12)	[0.00, 0.50]	0.048	0.56
96	36	0.57	(0.07)	35	0.44	(0.07)	0.14	(0.10)	[-0.07, 0.34]	0.18	0.14	(0.10)	[-0.06, 0.35]	0.17	0.10
Average treatment differences through follow-up+							0.09	(0.07)	[-0.04, 0.23]	0.18	0.10	(0.07)	[-0.04, 0.23]	0.17	0.21

S9.10 ODYSSEY A - changes in CD4/CD8 ratio over follow-up

Weeks since randomisation	DTG			SOC			Unadjusted Difference*			
	n	mean	(SE)	n	mean	(SE)	mean	(SE)	[95% CI]	p
4	29	0.19	(0.10)	30	0.36	(0.10)	-0.17	(0.14)	[-0.45 0.11]	0.23
12	30	0.10	(0.07)	33	0.18	(0.07)	-0.07	(0.10)	[-0.28 0.14]	0.50
24	29	0.35	(0.09)	31	0.22	(0.08)	0.13	(0.12)	[-0.12 0.38]	0.30
48	25	0.48	(0.09)	32	0.33	(0.08)	0.15	(0.13)	[-0.10 0.40]	0.24
72	27	0.51	(0.11)	29	0.32	(0.10)	0.19	(0.15)	[-0.10 0.49]	0.19
96	29	0.55	(0.09)	31	0.49	(0.08)	0.06	(0.12)	[-0.18 0.30]	0.63
Average treatment differences through follow-up+							0.05	(0.08)	[-0.11, 0.21]	0.56

*Change calculated using normal regression adjusting for baseline only. Presenting mean change from a baseline of 0.7 in total and 0.7 in A.
**Normal regression adjusting for baseline and ODYSSEY A/B strata.
+Linear mixed models fitted with random intercept and fixed effects for treatment group, study visit and adjustment covariates (baseline CD4/CD8 ratio and ODYSSEY A/B strata (total population only))
++ Test of heterogeneity of treatment effect between ODYSSEY A/B

S9.11 Total population - Changes in CD4/CD8 ratio over follow-up

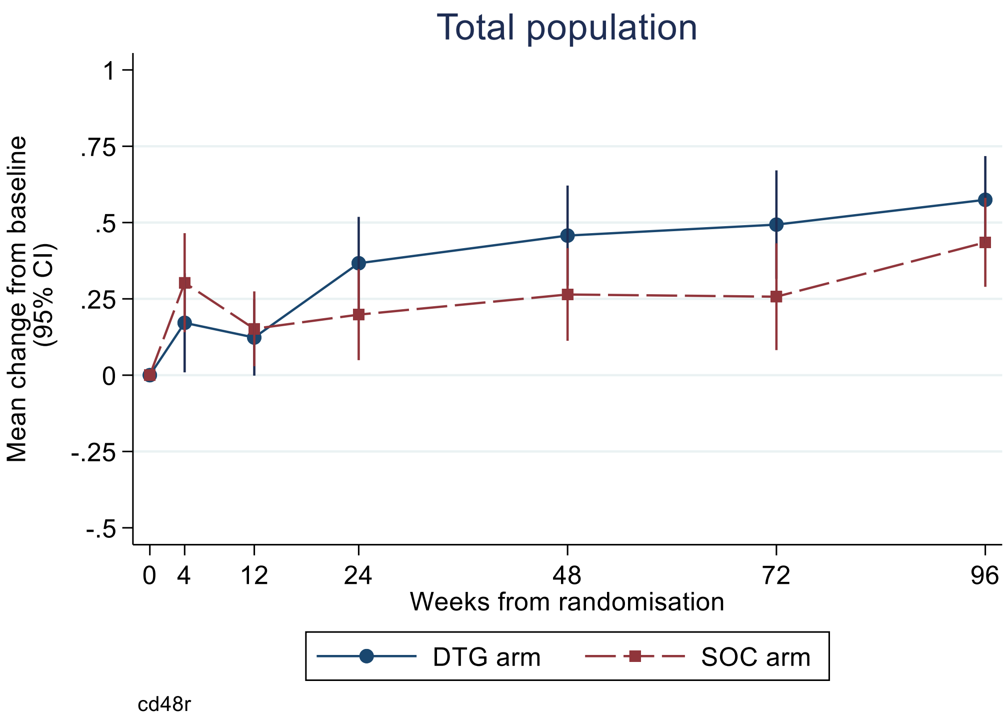

S9.12 ODYSSEY A - changes in CD4/CD8 ratio over follow-up

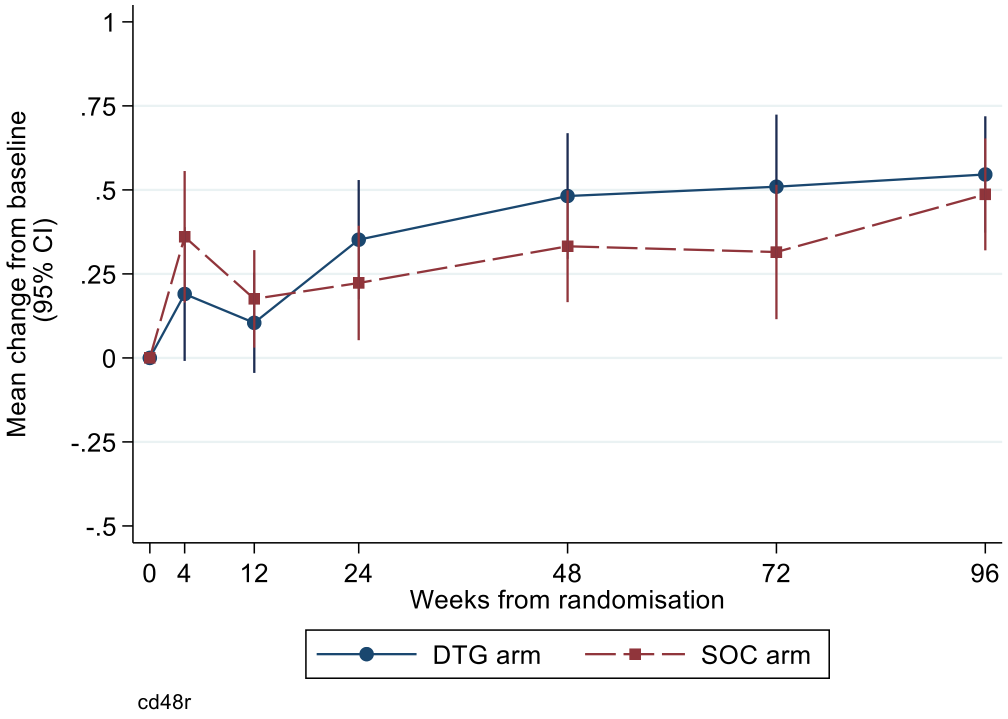

S10 Lipids

Note: ODYSSEY B is not presented here due to having few participants

Throughout this section, n refers to the number of participants with available measurement at each visit week. Numbers in models may be lower where there are missing data at baseline.

S10.1 Total population - Total cholesterol changes over follow-up

Weeks since randomisation	DTG			SOC			Unadjusted Difference*				Adjusted Difference**				Interaction++
	n	mean	(SE)	n	mean	(SE)	mean	(SE)	[95% CI]	p	mean	(SE)	[95% CI]	p	p
48	34	1.7	(4.8)	38	14.7	(4.8)	-13.0	(6.8)	[-26.6, 0.6]	0.061	-12.6	(6.7)	[-26.0, 0.9]	0.067	0.62
96	36	4.5	(5.6)	36	29.6	(5.9)	-25.2	(8.2)	[-41.5, -8.9]	0.0030	-24.4	(8.0)	[-40.3, -8.5]	0.0032	0.25
Average treatment differences through follow-up+							-18.6	(6.0)	[-30.4, -6.7]	0.0021	-18.2	(5.8)	[-29.6, -6.8]	0.0018	0.34

S10.2 ODYSSEY A - Total cholesterol changes over follow-up

Weeks since randomisation	DTG			SOC			Unadjusted Difference*			
	n	mean	(SE)	n	mean	(SE)	mean	(SE)	[95% CI]	p
48	27	4.0	(5.4)	32	18.4	(5.3)	-14.4	(7.6)	[-29.6, 0.8]	0.063
96	29	6.8	(6.2)	31	35.4	(6.5)	-28.6	(9.0)	[-46.7, -10.5]	0.0026
Average treatment differences through follow-up+							-21.0	(6.6)	[-33.8, -8.1]	0.0014

*Change calculated using normal regression adjusting for baseline only. Presenting mean change from a baseline of 127.9 in total and 127.7 in A.

**Normal regression adjusting for baseline and ODYSSEY A/B strata.

+Linear mixed models fitted with random intercept and fixed effects for treatment group, study visit and adjustment covariates (baseline Total cholesterol and ODYSSEY A/B strata (total population only))

++ Test of heterogeneity of treatment effect between ODYSSEY A/B

S10.3 Total population - changes in Total cholesterol over follow-up

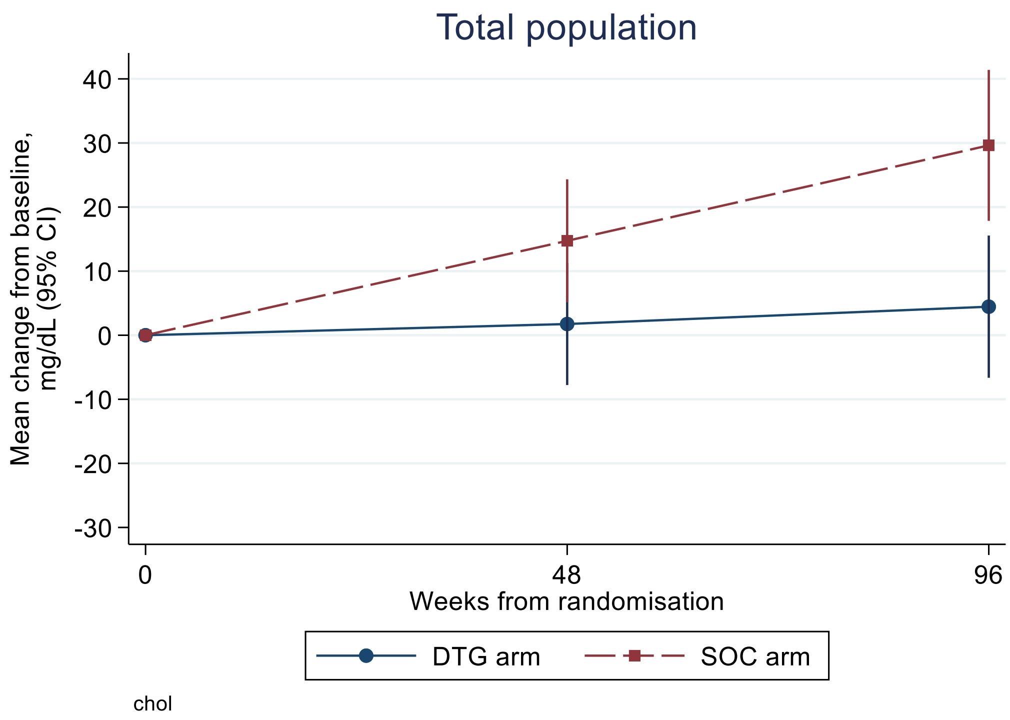

S10.4 ODYSSEY A - changes in Total cholesterol over follow-up

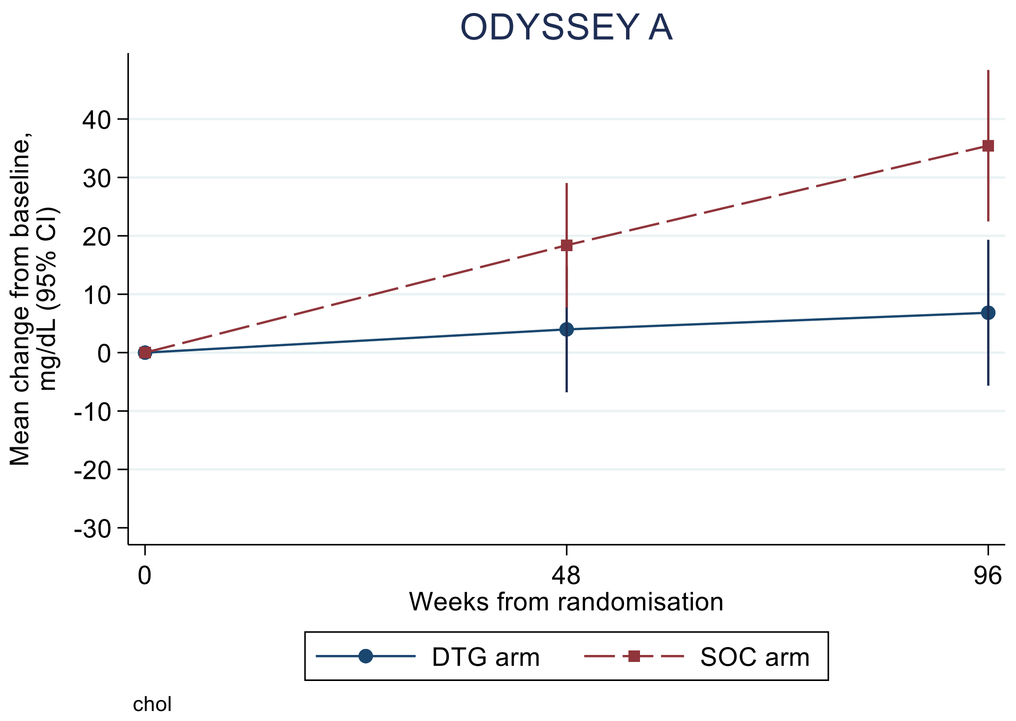

S10.5 Total population - LDL cholesterol changes over follow-up

Weeks since randomisation	DTG			SOC			Unadjusted Difference*				Adjusted Difference**				Interaction++
	n	mean	(SE)	n	mean	(SE)	mean	(SE)	[95% CI]	p	mean	(SE)	[95% CI]	p	p
48	34	-2.5	(3.9)	38	13.6	(3.7)	-16.1	(5.4)	[-26.9, -5.4]	0.0040	-16.0	(5.4)	[-26.8, -5.1]	0.0046	0.79
96	36	-3.2	(5.1)	36	22.4	(5.3)	-25.7	(7.4)	[-40.4, -11.0]	>0.0001	-24.9	(7.3)	[-39.5, -10.4]	0.0011	0.35
Average treatment differences through follow-up+							-20.3	(5.2)	[-30.4, -10.1]	>0.0001	-20.0	(5.1)	[-30.0, -9.9]	>0.0001	0.76

S10.6 ODYSSEY A - LDL cholesterol changes over follow-up

Weeks since randomisation	DTG			SOC			Unadjusted Difference*			
	n	mean	(SE)	n	mean	(SE)	mean	(SE)	[95% CI]	p
48	27	-1.3	(4.2)	32	14.0	(3.9)	-15.3	(5.7)	[-26.9, -3.8]	0.010
96	29	-1.1	(6.1)	31	27.1	(6.1)	-28.2	(8.6)	[-45.5, -11.0]	0.0019
Average treatment differences through follow-up+							-20.7	(6.0)	[-32.4, -9.0]	0.00051

*Change calculated using normal regression adjusting for baseline only. Presenting mean change from a baseline of 69.8 in total and 69.4 in A.

**Normal regression adjusting for baseline and ODYSSEY A/B strata.

+Linear mixed models fitted with random intercept and fixed effects for treatment group, study visit and adjustment covariates (baseline LDL cholesterol and ODYSSEY A/B strata (total population only))

++ Test of heterogeneity of treatment effect between ODYSSEY A/B

S10.7 Total population - changes in LDL cholesterol over follow-up

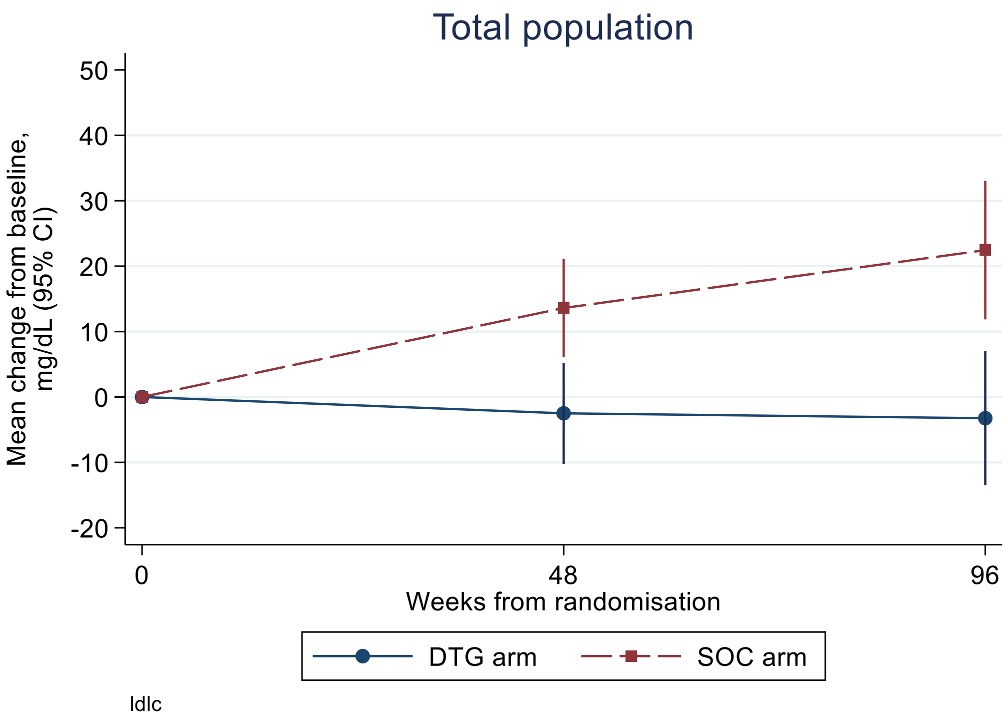

S10.8 ODYSSEY A - changes in LDL cholesterol over follow-up

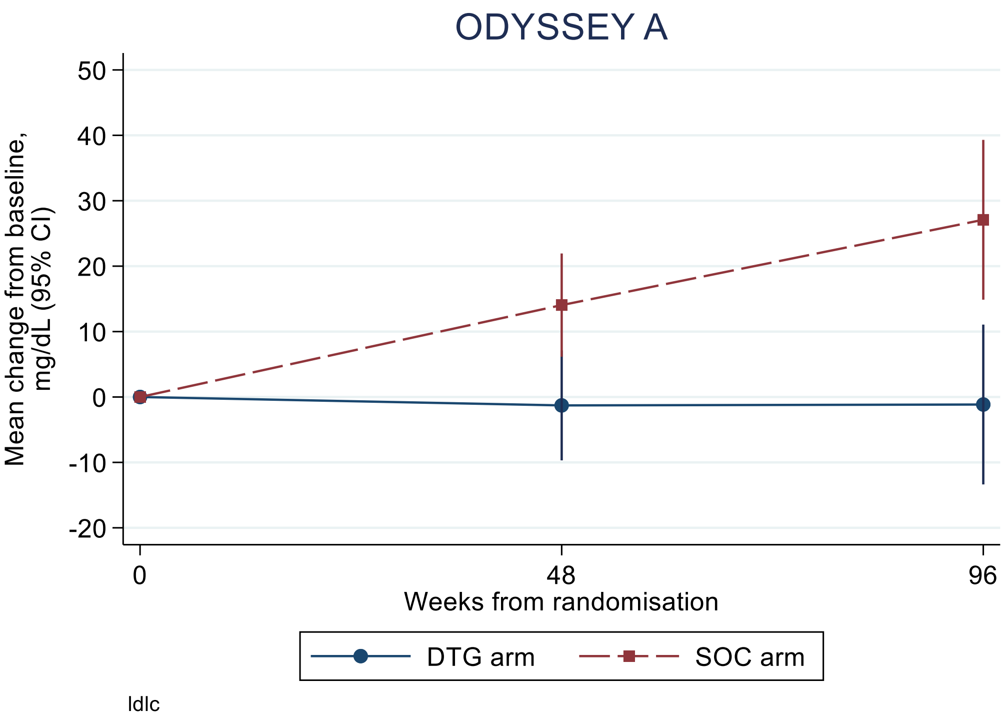

S10.9 Total population - HDL cholesterol changes over follow-up

Weeks since randomisation	DTG			SOC			Unadjusted Difference*				Adjusted Difference**				Interaction++
	n	mean	(SE)	n	mean	(SE)	mean	(SE)	[95% CI]	p	mean	(SE)	[95% CI]	p	p
48	34	9.0	(2.5)	38	10.2	(2.4)	-1.2	(3.5)	[-8.2, 5.9]	0.74	-1.2	(3.6)	[-8.3, 5.9]	0.74	0.18
96	36	13.0	(2.6)	36	16.5	(2.7)	-3.6	(3.7)	[-11.0, 3.9]	0.35	-3.0	(3.6)	[-10.3, 4.3]	0.41	0.17
Average treatment differences through follow-up+							-2.3	(2.6)	[-7.3, 2.7]	0.37	-2.1	(2.5)	[-7.0, 2.9]	0.41	0.97

S10.10 ODYSSEY A - HDL cholesterol changes over follow-up

Weeks since randomisation	DTG			SOC			Unadjusted Difference*			
	n	mean	(SE)	n	mean	(SE)	mean	(SE)	[95% CI]	p
48	27	10.2	(2.9)	32	8.9	(2.7)	1.2	(4.0)	[-6.8, 9.3]	0.76
96	29	13.6	(2.9)	31	19.2	(2.8)	-5.6	(4.0)	[-13.6, 2.4]	0.17
Average treatment differences through follow-up+							-2.1	(2.8)	[-7.6, 3.3]	0.44

*Change calculated using normal regression adjusting for baseline only. Presenting mean change from a baseline of 34.0 in total and 34.1 in A.

**Normal regression adjusting for baseline and ODYSSEY A/B strata.

+Linear mixed models fitted with random intercept and fixed effects for treatment group, study visit and adjustment covariates (baseline HDL cholesterol and ODYSSEY A/B strata (total population only))

++ Test of heterogeneity of treatment effect between ODYSSEY A/B

S10.11 Total population - changes in HDL cholesterol over follow-up

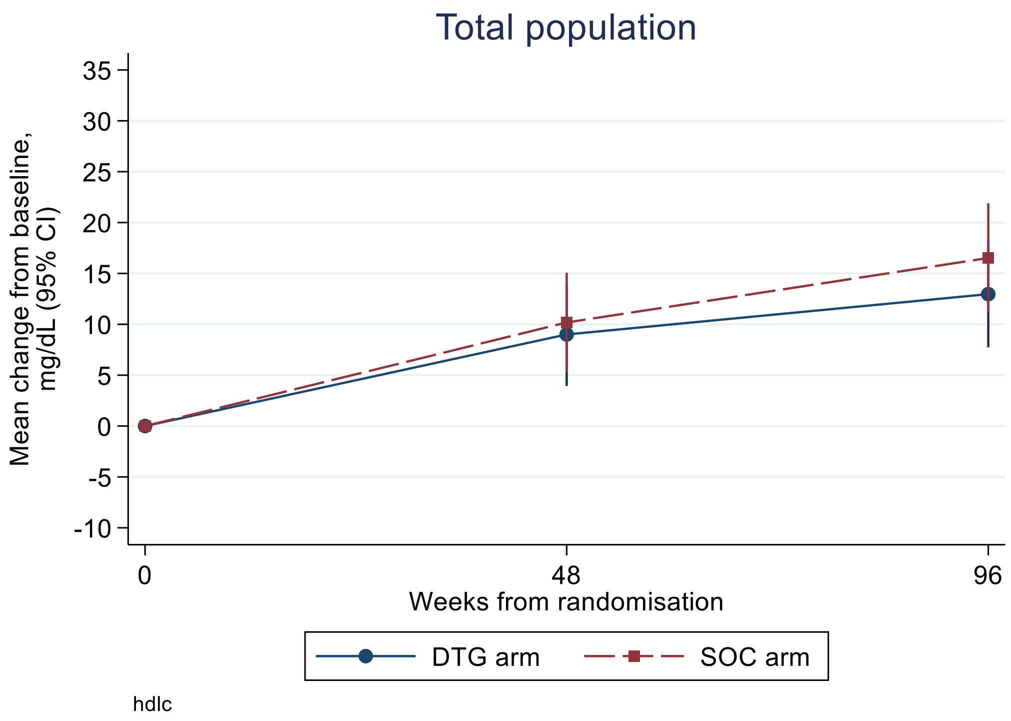

S10.12 ODYSSEY A - changes in HDL cholesterol over follow-up

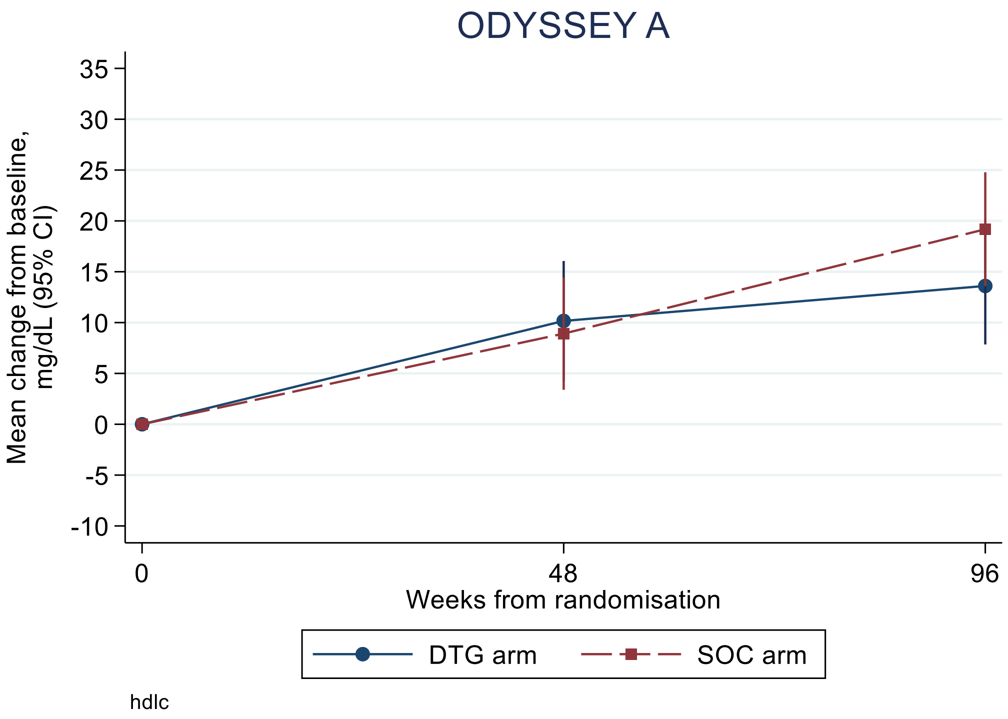

S10.13 Total population - Triglycerides changes over follow-up

Weeks since randomisation	DTG			SOC			Unadjusted Difference*				Adjusted Difference**				Interaction++
	n	mean	(SE)	n	mean	(SE)	mean	(SE)	[95% CI]	p	mean	(SE)	[95% CI]	p	p
48	33	-0.9	(14.9)	37	-28.5	(14.7)	27.6	(21.0)	[-14.5, 69.6]	0.19	28.1	(21.0)	[-13.9, 70.1]	0.19	0.23
96	34	-39.8	(10.5)	34	-30.3	(10.9)	-9.5	(15.3)	[-40.0, 21.1]	0.54	-9.0	(15.3)	[-39.6, 21.7]	0.56	0.73
Average treatment differences through follow-up+							9.9	(13.9)	[-17.4, 37.2]	0.48	10.4	(13.8)	[-16.6, 37.4]	0.45	0.42

S10.14 ODYSSEY A - Triglycerides changes over follow-up

Weeks since randomisation	DTG			SOC			Unadjusted Difference*			
	n	mean	(SE)	n	mean	(SE)	mean	(SE)	[95% CI]	p
48	26	-4.7	(16.0)	31	-21.1	(15.6)	16.4	(22.3)	[-28.4, 61.2]	0.46
96	27	-39.2	(12.4)	29	-32.7	(12.7)	-6.5	(17.7)	[-42.2, 29.1]	0.71
Average treatment differences through follow-up+							5.7	(15.4)	[-24.5, 35.9]	0.71

*Change calculated using normal regression adjusting for baseline only. Presenting mean change from a baseline of 159.2 in total and 163.6 in A.

**Normal regression adjusting for baseline and ODYSSEY A/B strata.

+Linear mixed models fitted with random intercept and fixed effects for treatment group, study visit and adjustment covariates (baseline Triglycerides and ODYSSEY A/B strata (total population only))

++ Test of heterogeneity of treatment effect between ODYSSEY A/B

S10.15 Total population - changes in Triglycerides over follow-up

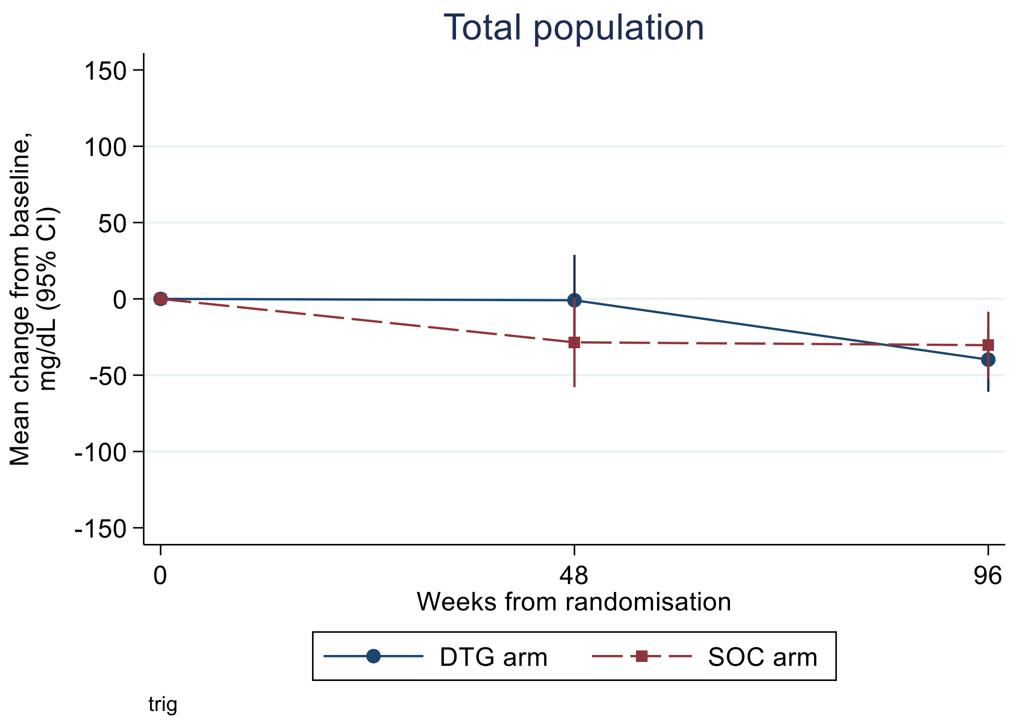

S10.16 ODYSSEY A - changes in Triglycerides over follow-up

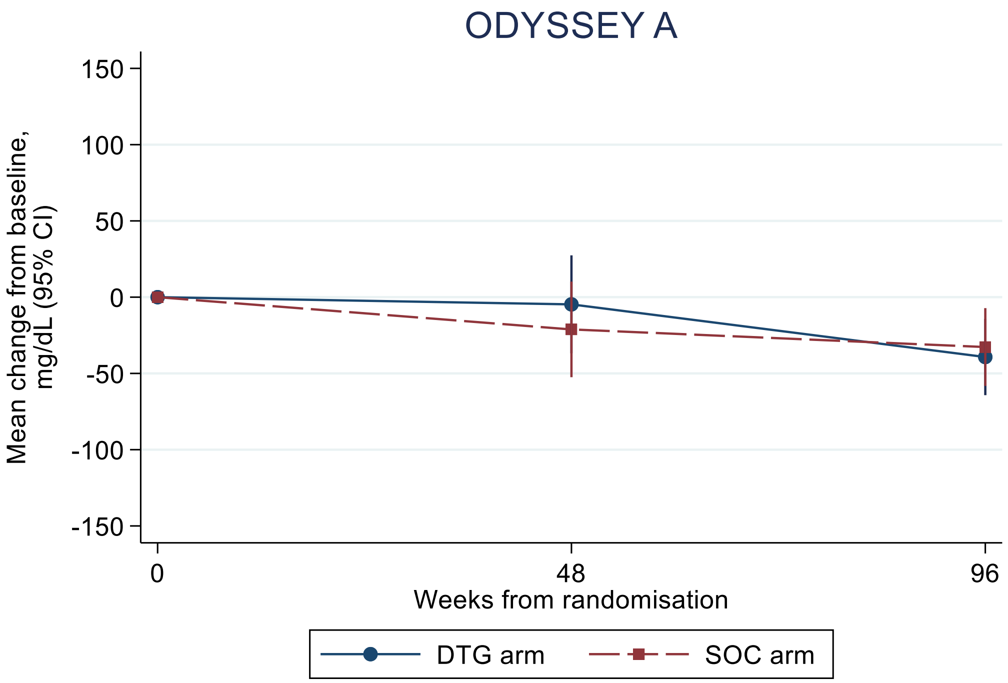

trig

S10.17 Total population - Glucose changes over follow-up

Weeks since randomisation	DTG			SOC			Unadjusted Difference*				Adjusted Difference**				Interaction++
	n	mean	(SE)	n	mean	(SE)	mean	(SE)	[95% CI]	p	mean	(SE)	[95% CI]	p	p
48	31	1.0	(2.8)	37	-0.7	(2.7)	1.7	(3.9)	[-6.0, 9.5]	0.66	1.9	(3.9)	[-5.8, 9.7]	0.62	0.51
96	33	-0.7	(2.2)	34	4.8	(2.4)	-5.5	(3.3)	[-12.0, 1.0]	0.098	-5.4	(3.3)	[-12.0, 1.2]	0.11	0.17
Average treatment differences through follow-up+							-2.0	(2.6)	[-7.0, 3.0]	0.43	-1.8	(2.5)	[-6.8, 3.1]	0.47	0.77

S10.18 ODYSSEY A - Glucose changes over follow-up

Weeks since randomisation	DTG			SOC			Unadjusted Difference*			
	n	mean	(SE)	n	mean	(SE)	mean	(SE)	[95% CI]	p
48	24	1.6	(3.2)	31	1.0	(3.1)	0.6	(4.4)	[-8.3, 9.4]	0.90
96	26	0.5	(2.5)	29	3.9	(2.7)	-3.4	(3.7)	[-10.7, 4.0]	0.36
Average treatment differences through follow-up+							-1.5	(2.8)	[-7.0, 3.9]	0.58

*Change calculated using normal regression adjusting for baseline only. Presenting mean change from a baseline of 81.7 in total and 82.3 in A.
**Normal regression adjusting for baseline and ODYSSEY A/B strata.
+Linear mixed models fitted with random intercept and fixed effects for treatment group, study visit and adjustment covariates (baseline Glucose and ODYSSEY A/B strata (total population only))
++ Test of heterogeneity of treatment effect between ODYSSEY A/B

S10.19 Total population - changes in Glucose over follow-up

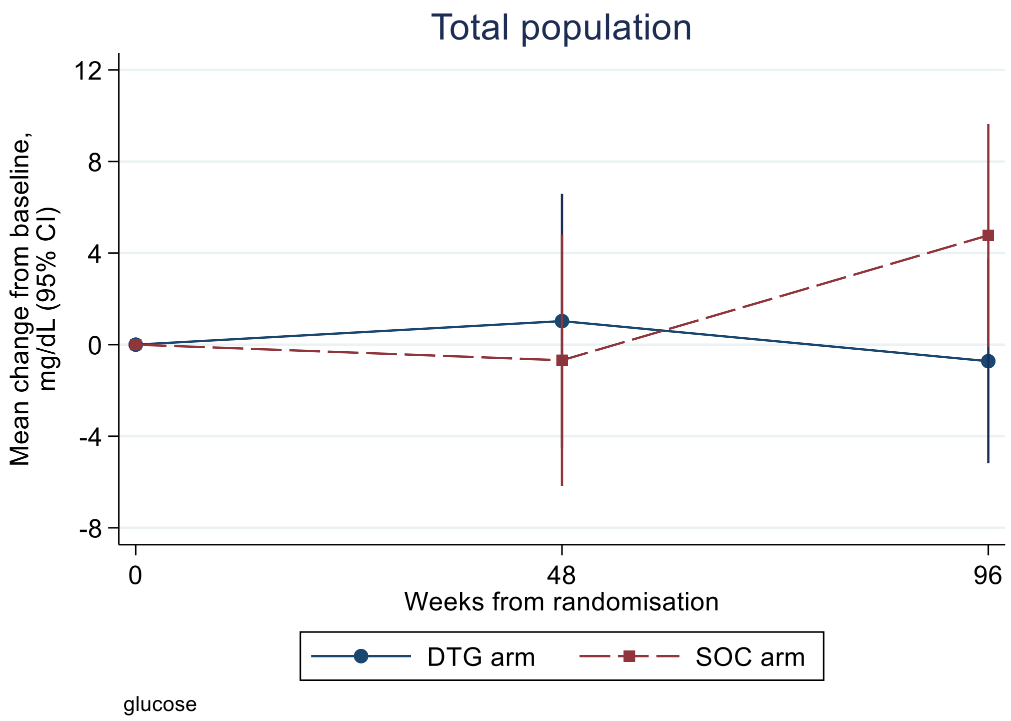

S10.20 ODYSSEY A - changes in Glucose over follow-up

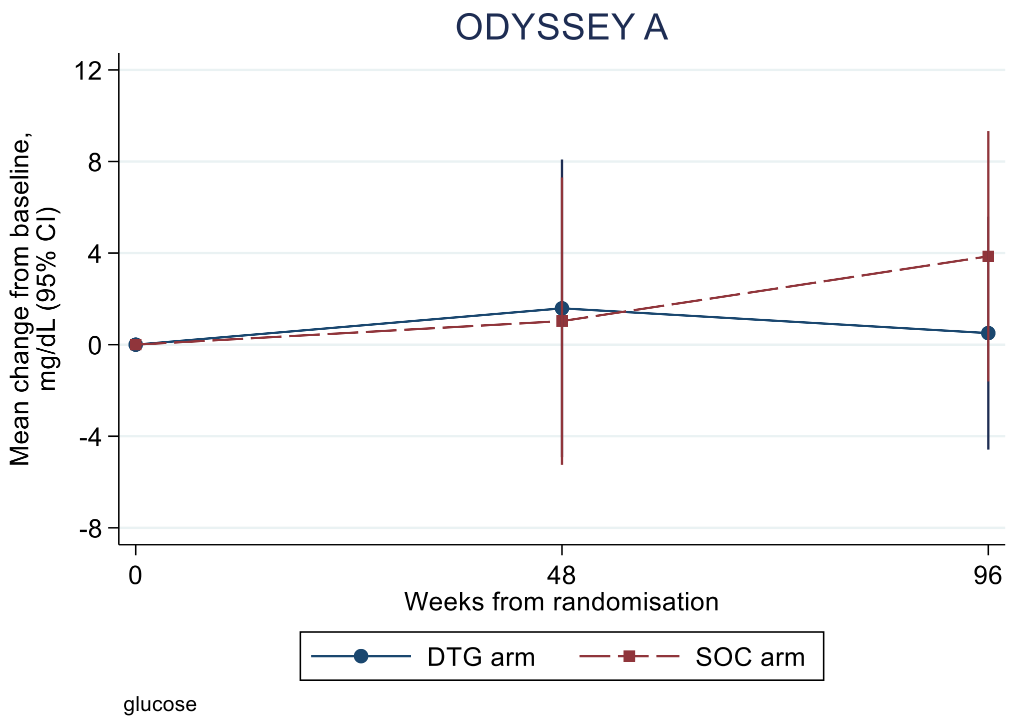

S11 Adverse Events

S11.1 Serious Adverse Events (SAEs) to trial censoring date by SAE type*

	Total			A			B		
	**DTG**	**SOC**	**Total**	**DTG**	**SOC**	**Total**	**DTG**	**SOC**	**Total**
**Participants randomised and included**	42	43	85	35	37	72	7	6	13
Number of events [Number of participants]	15 [11]	19 [11]	34 [22]	13 [10]	18 [10]	31 [20]	2 [1]	1 [1]	3 [2]
Death	2 [2]	4 [4]	6 [6]	2 [2]	3 [3]	5 [5]	0 [0]	1 [1]	1 [1]
Hospitalisation	12 [9]	14 [7]	26 [16]	10 [8]	14 [7]	24 [15]	2 [1]	0 [0]	2 [1]
Other	1 [1]	1 [1]	2 [2]	1 [1]	1 [1]	2 [2]	0 [0]	0 [0]	0 [0]

*Non-overlapping categories, worst taken.

S11.2 Serious Adverse Events to trial censoring date

	Total			A			B		
	**DTG**	**SOC**	**Total**	**DTG**	**SOC**	**Total**	**DTG**	**SOC**	**Total**
**Participants randomised and included**	42	43	85	35	37	72	7	6	13
**Person years**	93	95	188	76	81	156	18	14	32
**Number of events [Number of young people]**	15 [11]	19 [11]	34 [22]	13 [10]	18 [10]	31 [20]	2 [1]	1 [1]	3 [2]
**Biochemical**	0 [0]	1 [1]	1 [1]	0 [0]	1 [1]	1 [1]	0 [0]	0 [0]	0 [0]
Raised liver enzymes	0 [0]	1 [1]	1 [1]	0 [0]	1 [1]	1 [1]	0 [0]	0 [0]	0 [0]
**Haematological**	1 [1]	1 [1]	2 [2]	1 [1]	1 [1]	2 [2]	0 [0]	0 [0]	0 [0]
Thrombocytopenia	0 [0]	1 [1]	1 [1]	0 [0]	1 [1]	1 [1]	0 [0]	0 [0]	0 [0]
Thrombocytopenia+Neutropenia	1 [1]	0 [0]	1 [1]	1 [1]	0 [0]	1 [1]	0 [0]	0 [0]	0 [0]
**Infectious Disease**	10 [9]	16 [9]	26 [18]	9 [8]	15 [8]	24 [16]	1 [1]	1 [1]	2 [2]
Acute diarrhoea not investigated+Dehydration	1 [1]	0 [0]	1 [1]	1 [1]	0 [0]	1 [1]	0 [0]	0 [0]	0 [0]
Bronchiolitis	0 [0]	1 [1]	1 [1]	0 [0]	1 [1]	1 [1]	0 [0]	0 [0]	0 [0]
Chest infection	1 [1]	1 [1]	2 [2]	0 [0]	1 [1]	1 [1]	1 [1]	0 [0]	1 [1]
Chronic diarrhoea with no pathogen	1 [1]	0 [0]	1 [1]	1 [1]	0 [0]	1 [1]	0 [0]	0 [0]	0 [0]
Encephalitis - presumed infectious	1 [1]	0 [0]	1 [1]	1 [1]	0 [0]	1 [1]	0 [0]	0 [0]	0 [0]
Gastroenteritis	1 [1]	0 [0]	1 [1]	1 [1]	0 [0]	1 [1]	0 [0]	0 [0]	0 [0]
Gastroenteritis *	0 [0]	1 [1]	1 [1]	0 [0]	0 [0]	0 [0]	0 [0]	1 [1]	1 [1]
Measles	0 [0]	1 [1]	1 [1]	0 [0]	1 [1]	1 [1]	0 [0]	0 [0]	0 [0]
Measles+Chest infection	1 [1]	0 [0]	1 [1]	1 [1]	0 [0]	1 [1]	0 [0]	0 [0]	0 [0]
P falciparum malaria	1 [1]	0 [0]	1 [1]	1 [1]	0 [0]	1 [1]	0 [0]	0 [0]	0 [0]
Pneumonia - other bacterial	0 [0]	1 [1]	1 [1]	0 [0]	1 [1]	1 [1]	0 [0]	0 [0]	0 [0]
Pneumonia no organism identified, aspiration pneumonia	1 [1]	1 [1]	2 [2]	1 [1]	1 [1]	2 [2]	0 [0]	0 [0]	0 [0]
Pneumonia no organism identified, aspiration pneumonia *	0 [0]	1 [1]	1 [1]	0 [0]	1 [1]	1 [1]	0 [0]	0 [0]	0 [0]
Pneumonia no organism identified, aspiration pneumonia+Acute otitis media	1 [1]	0 [0]	1 [1]	1 [1]	0 [0]	1 [1]	0 [0]	0 [0]	0 [0]
Presumed septicaemia/bacteremia - no organism	0 [0]	1 [1]	1 [1]	0 [0]	1 [1]	1 [1]	0 [0]	0 [0]	0 [0]
Pyogenic meningitis - organism	0 [0]	1 [1]	1 [1]	0 [0]	1 [1]	1 [1]	0 [0]	0 [0]	0 [0]
Tuberculosis - abdominal	0 [0]	1 [1]	1 [1]	0 [0]	1 [1]	1 [1]	0 [0]	0 [0]	0 [0]
Tuberculosis - disseminated/miliary	0 [0]	2 [1]	2 [1]	0 [0]	2 [1]	2 [1]	0 [0]	0 [0]	0 [0]
Tuberculosis - pulmonary - smear negative or not done	1 [1]	1 [1]	2 [2]	1 [1]	1 [1]	2 [2]	0 [0]	0 [0]	0 [0]
Tuberculosis - pulmonary - smear negative or not done+Severe malnutrition	0 [0]	2 [1]	2 [1]	0 [0]	2 [1]	2 [1]	0 [0]	0 [0]	0 [0]
Tuberculosis - pulmonary - smear negative or not done+Severe malnutrition *	0 [0]	1 [1]	1 [1]	0 [0]	1 [1]	1 [1]	0 [0]	0 [0]	0 [0]
**Non HIV related deaths**	1 [1]	0 [0]	1 [1]	1 [1]	0 [0]	1 [1]	0 [0]	0 [0]	0 [0]
Traumatic *	1 [1]	0 [0]	1 [1]	1 [1]	0 [0]	1 [1]	0 [0]	0 [0]	0 [0]
**Systemic**	3 [3]	1 [1]	4 [4]	2 [2]	1 [1]	3 [3]	1 [1]	0 [0]	1 [1]
Chest infection+Severe malnutrition	1 [1]	0 [0]	1 [1]	0 [0]	0 [0]	0 [0]	1 [1]	0 [0]	1 [1]
Kwashiorkor	1 [1]	0 [0]	1 [1]	1 [1]	0 [0]	1 [1]	0 [0]	0 [0]	0 [0]
Kwashiorkor *	1 [1]	0 [0]	1 [1]	1 [1]	0 [0]	1 [1]	0 [0]	0 [0]	0 [0]
Severe malnutrition *	0 [0]	1 [1]	1 [1]	0 [0]	1 [1]	1 [1]	0 [0]	0 [0]	0 [0]
**Event rate (all events)**									
Event rate (per 100 person years)	16.1	20.0	18.1	17.2	22.3	19.8	11.3	7.1	9.4
(95% CI)	(9.7, 26.6)	(12.8, 31.4)	(12.9, 25.3)	(10.0, 29.6)	(14.0, 35.4)	(13.9, 28.2)	(2.8, 45.0)	(1.0, 50.2)	(3.0, 29.2)
Unadjusted Rate ratio	0.80	1 (ref)		0.77	1 (ref)		1.59	1 (ref)	
(95% CI)	(0.32, 2.04)	-		(0.29, 2.06)	-		(0.10, 24.54)		
P-value			0.64			0.61			0.74
Adj. Rate ratio**	0.82	1 (ref)							
(95% CI)	(0.32, 2.08)	-							
P-value			0.68						
**Time to first event**									
Unadjusted Hazard ratio	1.06	1 (ref)		1.09	1 (ref)		0.93	1 (ref)	
(95% CI)	(0.46, 2.45)	-		(0.45, 2.62)	-		(0.06, 14.83)	-	
P-value			0.89			0.85			0.96

Adj. hazard ratio**	1.08	1 (ref)							
(95% CI)	(0.47, 2.49)	-							
P-value			0.86						
A/B x arm interaction	0.90								

*Resulted in death

**Adjusted for ODYSSEY A and B strata in total.

S11.3 Grade 3 or above clinical and laboratory adverse events to trial censoring date

	Total			A			B		
	**DTG**	**SOC**	**Total**	**DTG**	**SOC**	**Total**	**DTG**	**SOC**	**Total**
**Participants randomised and included**	42	43	85	35	37	72	7	6	13
**Person years**	93	95	188	76	81	156	18	14	32
**Number of events [Number of young people]**	36 [19]	34 [21]	70 [40]	29 [17]	33 [20]	62 [37]	7 [2]	1 [1]	8 [3]
**Biochemical**	4 [4]	3 [2]	7 [6]	3 [3]	3 [2]	6 [5]	1 [1]	0 [0]	1 [1]
Raised ALT	0 [0]	1 [1]	1 [1]	0 [0]	1 [1]	1 [1]	0 [0]	0 [0]	0 [0]
Raised AST	2 [2]	0 [0]	2 [2]	1 [1]	0 [0]	1 [1]	1 [1]	0 [0]	1 [1]
Raised alkaline phosphatase (ALK)	1 [1]	0 [0]	1 [1]	1 [1]	0 [0]	1 [1]	0 [0]	0 [0]	0 [0]
Raised cholesterol	0 [0]	1 [1]	1 [1]	0 [0]	1 [1]	1 [1]	0 [0]	0 [0]	0 [0]
Raised liver enzymes	0 [0]	1 [1]	1 [1]	0 [0]	1 [1]	1 [1]	0 [0]	0 [0]	0 [0]
Raised triglycerides	1 [1]	0 [0]	1 [1]	1 [1]	0 [0]	1 [1]	0 [0]	0 [0]	0 [0]
**Haematological**	12 [10]	13 [12]	25 [22]	10 [8]	13 [12]	23 [20]	2 [2]	0 [0]	2 [2]
Anaemia with no clinical symptoms	6 [6]	8 [8]	14 [14]	5 [5]	8 [8]	13 [13]	1 [1]	0 [0]	1 [1]
Leucopenia	0 [0]	1 [1]	1 [1]	0 [0]	1 [1]	1 [1]	0 [0]	0 [0]	0 [0]
Neutropenia	3 [3]	1 [1]	4 [4]	2 [2]	1 [1]	3 [3]	1 [1]	0 [0]	1 [1]
Thrombocytopenia	3 [3]	3 [3]	6 [6]	3 [3]	3 [3]	6 [6]	0 [0]	0 [0]	0 [0]
**Hepatic**	0 [0]	1 [1]	1 [1]	0 [0]	1 [1]	1 [1]	0 [0]	0 [0]	0 [0]
drug induced liver injury	0 [0]	1 [1]	1 [1]	0 [0]	1 [1]	1 [1]	0 [0]	0 [0]	0 [0]
**Infectious Disease**	15 [11]	15 [11]	30 [22]	12 [9]	14 [10]	26 [19]	3 [2]	1 [1]	4 [3]
Acute diarrhoea not investigated	2 [2]	0 [0]	2 [2]	2 [2]	0 [0]	2 [2]	0 [0]	0 [0]	0 [0]
Acute otitis media	1 [1]	0 [0]	1 [1]	1 [1]	0 [0]	1 [1]	0 [0]	0 [0]	0 [0]
Bronchiolitis	0 [0]	1 [1]	1 [1]	0 [0]	1 [1]	1 [1]	0 [0]	0 [0]	0 [0]
Chest infection	3 [2]	1 [1]	4 [3]	1 [1]	1 [1]	2 [2]	2 [1]	0 [0]	2 [1]
Chronic diarrhoea not investigated	0 [0]	1 [1]	1 [1]	0 [0]	1 [1]	1 [1]	0 [0]	0 [0]	0 [0]
Chronic diarrhoea with no pathogen	1 [1]	0 [0]	1 [1]	1 [1]	0 [0]	1 [1]	0 [0]	0 [0]	0 [0]
Encephalitis - presumed infectious	1 [1]	0 [0]	1 [1]	1 [1]	0 [0]	1 [1]	0 [0]	0 [0]	0 [0]
Gastroenteritis	1 [1]	0 [0]	1 [1]	1 [1]	0 [0]	1 [1]	0 [0]	0 [0]	0 [0]
Gastroenteritis *	0 [0]	1 [1]	1 [1]	0 [0]	0 [0]	0 [0]	0 [0]	1 [1]	1 [1]
Hepatitis A	1 [1]	0 [0]	1 [1]	0 [0]	0 [0]	0 [0]	1 [1]	0 [0]	1 [1]
Internal abscess	0 [0]	1 [1]	1 [1]	0 [0]	1 [1]	1 [1]	0 [0]	0 [0]	0 [0]
Measles	1 [1]	1 [1]	2 [2]	1 [1]	1 [1]	2 [2]	0 [0]	0 [0]	0 [0]
P falciparum malaria	1 [1]	0 [0]	1 [1]	1 [1]	0 [0]	1 [1]	0 [0]	0 [0]	0 [0]
Pneumonia - other bacterial	0 [0]	1 [1]	1 [1]	0 [0]	1 [1]	1 [1]	0 [0]	0 [0]	0 [0]
Pneumonia no organism identified, aspiration pneumonia	2 [2]	0 [0]	2 [2]	2 [2]	0 [0]	2 [2]	0 [0]	0 [0]	0 [0]
Pneumonia no organism identified, aspiration pneumonia *	0 [0]	1 [1]	1 [1]	0 [0]	1 [1]	1 [1]	0 [0]	0 [0]	0 [0]
Presumed septicaemia/bacteremia - no organism	0 [0]	1 [1]	1 [1]	0 [0]	1 [1]	1 [1]	0 [0]	0 [0]	0 [0]
Pyogenic meningitis - organism	0 [0]	1 [1]	1 [1]	0 [0]	1 [1]	1 [1]	0 [0]	0 [0]	0 [0]
Tuberculosis - abdominal	0 [0]	1 [1]	1 [1]	0 [0]	1 [1]	1 [1]	0 [0]	0 [0]	0 [0]
Tuberculosis - disseminated/miliary	0 [0]	1 [1]	1 [1]	0 [0]	1 [1]	1 [1]	0 [0]	0 [0]	0 [0]
Tuberculosis - pulmonary - smear negative or not done	1 [1]	2 [2]	3 [3]	1 [1]	2 [2]	3 [3]	0 [0]	0 [0]	0 [0]
Tuberculosis - pulmonary - smear negative or not done *	0 [0]	1 [1]	1 [1]	0 [0]	1 [1]	1 [1]	0 [0]	0 [0]	0 [0]
**Non HIV related deaths**	1 [1]	0 [0]	1 [1]	1 [1]	0 [0]	1 [1]	0 [0]	0 [0]	0 [0]
Traumatic *	1 [1]	0 [0]	1 [1]	1 [1]	0 [0]	1 [1]	0 [0]	0 [0]	0 [0]
**Systemic**	4 [4]	2 [2]	6 [6]	3 [3]	2 [2]	5 [5]	1 [1]	0 [0]	1 [1]
Dehydration	1 [1]	0 [0]	1 [1]	1 [1]	0 [0]	1 [1]	0 [0]	0 [0]	0 [0]
Kwashiorkor	1 [1]	0 [0]	1 [1]	1 [1]	0 [0]	1 [1]	0 [0]	0 [0]	0 [0]
Kwashiorkor *	1 [1]	0 [0]	1 [1]	1 [1]	0 [0]	1 [1]	0 [0]	0 [0]	0 [0]
Severe malnutrition	1 [1]	0 [0]	1 [1]	0 [0]	0 [0]	0 [0]	1 [1]	0 [0]	1 [1]
Severe malnutrition *	0 [0]	2 [2]	2 [2]	0 [0]	2 [2]	2 [2]	0 [0]	0 [0]	0 [0]
**Event rate (all events)**									
Event rate (per 100 person years)	38.5	35.8	37.2	38.3	40.8	39.6	39.4	7.1	25.1

(95% CI)	(27.8, 53.4)	(25.6, 50.1)	(29.4, 47.0)	(26.6, 55.2)	(29.0, 57.4)	(30.9, 50.8)	(18.8, 82.7)	(1.0, 50.2)	(12.5, 50.2)
Unadjusted Rate ratio	1.08	1 (ref)		0.94	1 (ref)		5.57	1 (ref)	
(95% CI)	(0.59, 1.96)	-		(0.51, 1.73)	-		(0.53, 58.84)	-	
P-value			0.81			0.84			0.15
Adj. Rate ratio**	1.09	1 (ref)							
(95% CI)	(0.61, 1.97)	-							
P-value			0.76						
**Time to first event**									
Unadjusted Hazard ratio	0.92	1 (ref)		0.88	1 (ref)		2.03	1 (ref)	
(95% CI)	(0.50, 1.72)	-		(0.46, 1.68)	-		(0.18, 22.45)	-	
P-value			0.80			0.70			0.57
Adj. hazard ratio**	0.93	1 (ref)							
(95% CI)	(0.50, 1.74)	-							
P-value			0.83						
A/B x arm interaction	0.55								

*Resulted in death

**Adjusted for ODYSSEY A and B strata in total.

S11.4 Adverse events leading to ART modification (any grade) to trial censoring date

	Total			A			B		
	**DTG**	**SOC**	**Total**	**DTG**	**SOC**	**Total**	**DTG**	**SOC**	**Total**
**Participants randomised and included**	42	43	85	35	37	72	7	6	13
**Person years**	93	95	188	76	81	156	18	14	32
**Number of events [Number of young people+]**	0 [0]	2 [2]	2 [2]	0 [0]	2 [2]	2 [2]	0 [0]	0 [0]	0 [0]
**Biochemical**	0 [0]	1 [1]	1 [1]	0 [0]	1 [1]	1 [1]	0 [0]	0 [0]	0 [0]
Raised liver enzymes	0 [0]	1 [1]	1 [1]	0 [0]	1 [1]	1 [1]	0 [0]	0 [0]	0 [0]
**Gastrointestinal**	0 [0]	1 [1]	1 [1]	0 [0]	1 [1]	1 [1]	0 [0]	0 [0]	0 [0]
Vomiting	0 [0]	1 [1]	1 [1]	0 [0]	1 [1]	1 [1]	0 [0]	0 [0]	0 [0]

+ Raised Liver Enzymes - Event is considered ART modifying as the event led to the participant stopping their ART regimen. The participant did subsequently re-start the same ART and hence is not contained within the table of treatment changes. Vomiting - Event led to substitution of 3rd agent due to adverse event.

S11.5 Serious Adverse Events (SAEs) to week 96 by SAE type*

	Total			A			B		
	**DTG**	**SOC**	**Total**	**DTG**	**SOC**	**Total**	**DTG**	**SOC**	**Total**
**Participants randomised and included**	42	43	85	35	37	72	7	6	13
Number of events [Number of participants]	15 [11]	19 [11]	34 [22]	13 [10]	18 [10]	31 [20]	2 [1]	1 [1]	3 [2]
Death	2 [2]	4 [4]	6 [6]	2 [2]	3 [3]	5 [5]	0 [0]	1 [1]	1 [1]
Hospitalisation	12 [9]	14 [7]	26 [16]	10 [8]	14 [7]	24 [15]	2 [1]	0 [0]	2 [1]
Other	1 [1]	1 [1]	2 [2]	1 [1]	1 [1]	2 [2]	0 [0]	0 [0]	0 [0]

*Non-overlapping categories, worst taken.

S11.6 Serious Adverse Events to week 96

	Total			A			B		
	**DTG**	**SOC**	**Total**	**DTG**	**SOC**	**Total**	**DTG**	**SOC**	**Total**
**Participants randomised and included**	42	43	85	35	37	72	7	6	13
**Person years**	70	73	143	57	62	120	13	11	23
**Number of events [Number of young people]**	15 [11]	19 [11]	34 [22]	13 [10]	18 [10]	31 [20]	2 [1]	1 [1]	3 [2]
**Biochemical**	0 [0]	1 [1]	1 [1]	0 [0]	1 [1]	1 [1]	0 [0]	0 [0]	0 [0]
Raised liver enzymes	0 [0]	1 [1]	1 [1]	0 [0]	1 [1]	1 [1]	0 [0]	0 [0]	0 [0]
**Haematological**	1 [1]	1 [1]	2 [2]	1 [1]	1 [1]	2 [2]	0 [0]	0 [0]	0 [0]
Thrombocytopenia	0 [0]	1 [1]	1 [1]	0 [0]	1 [1]	1 [1]	0 [0]	0 [0]	0 [0]
Thrombocytopenia+Neutropenia	1 [1]	0 [0]	1 [1]	1 [1]	0 [0]	1 [1]	0 [0]	0 [0]	0 [0]
**Infectious Disease**	10 [9]	16 [9]	26 [18]	9 [8]	15 [8]	24 [16]	1 [1]	1 [1]	2 [2]
Acute diarrhoea not investigated+Dehydration	1 [1]	0 [0]	1 [1]	1 [1]	0 [0]	1 [1]	0 [0]	0 [0]	0 [0]
Bronchiolitis	0 [0]	1 [1]	1 [1]	0 [0]	1 [1]	1 [1]	0 [0]	0 [0]	0 [0]
Chest infection	1 [1]	1 [1]	2 [2]	0 [0]	1 [1]	1 [1]	1 [1]	0 [0]	1 [1]
Chronic diarrhoea with no pathogen	1 [1]	0 [0]	1 [1]	1 [1]	0 [0]	1 [1]	0 [0]	0 [0]	0 [0]
Encephalitis - presumed infectious	1 [1]	0 [0]	1 [1]	1 [1]	0 [0]	1 [1]	0 [0]	0 [0]	0 [0]
Gastroenteritis	1 [1]	0 [0]	1 [1]	1 [1]	0 [0]	1 [1]	0 [0]	0 [0]	0 [0]
Gastroenteritis *	0 [0]	1 [1]	1 [1]	0 [0]	0 [0]	0 [0]	0 [0]	1 [1]	1 [1]
Measles	0 [0]	1 [1]	1 [1]	0 [0]	1 [1]	1 [1]	0 [0]	0 [0]	0 [0]
Measles+Chest infection	1 [1]	0 [0]	1 [1]	1 [1]	0 [0]	1 [1]	0 [0]	0 [0]	0 [0]
P falciparum malaria	1 [1]	0 [0]	1 [1]	1 [1]	0 [0]	1 [1]	0 [0]	0 [0]	0 [0]
Pneumonia - other bacterial	0 [0]	1 [1]	1 [1]	0 [0]	1 [1]	1 [1]	0 [0]	0 [0]	0 [0]
Pneumonia no organism identified, aspiration pneumonia	1 [1]	1 [1]	2 [2]	1 [1]	1 [1]	2 [2]	0 [0]	0 [0]	0 [0]
Pneumonia no organism identified, aspiration pneumonia *	0 [0]	1 [1]	1 [1]	0 [0]	1 [1]	1 [1]	0 [0]	0 [0]	0 [0]
Pneumonia no organism identified, aspiration pneumonia+Acute otitis media	1 [1]	0 [0]	1 [1]	1 [1]	0 [0]	1 [1]	0 [0]	0 [0]	0 [0]
Presumed septicaemia/bacteremia - no organism	0 [0]	1 [1]	1 [1]	0 [0]	1 [1]	1 [1]	0 [0]	0 [0]	0 [0]
Pyogenic meningitis - organism	0 [0]	1 [1]	1 [1]	0 [0]	1 [1]	1 [1]	0 [0]	0 [0]	0 [0]
Tuberculosis - abdominal	0 [0]	1 [1]	1 [1]	0 [0]	1 [1]	1 [1]	0 [0]	0 [0]	0 [0]
Tuberculosis - disseminated/miliary	0 [0]	2 [1]	2 [1]	0 [0]	2 [1]	2 [1]	0 [0]	0 [0]	0 [0]
Tuberculosis - pulmonary - smear negative or not done	1 [1]	1 [1]	2 [2]	1 [1]	1 [1]	2 [2]	0 [0]	0 [0]	0 [0]
Tuberculosis - pulmonary - smear negative or not done+Severe malnutrition	0 [0]	2 [1]	2 [1]	0 [0]	2 [1]	2 [1]	0 [0]	0 [0]	0 [0]
Tuberculosis - pulmonary - smear negative or not done+Severe malnutrition *	0 [0]	1 [1]	1 [1]	0 [0]	1 [1]	1 [1]	0 [0]	0 [0]	0 [0]
**Non HIV related deaths**	1 [1]	0 [0]	1 [1]	1 [1]	0 [0]	1 [1]	0 [0]	0 [0]	0 [0]
Traumatic *	1 [1]	0 [0]	1 [1]	1 [1]	0 [0]	1 [1]	0 [0]	0 [0]	0 [0]
**Systemic**	3 [3]	1 [1]	4 [4]	2 [2]	1 [1]	3 [3]	1 [1]	0 [0]	1 [1]
Chest infection+Severe malnutrition	1 [1]	0 [0]	1 [1]	0 [0]	0 [0]	0 [0]	1 [1]	0 [0]	1 [1]
Kwashiorkor	1 [1]	0 [0]	1 [1]	1 [1]	0 [0]	1 [1]	0 [0]	0 [0]	0 [0]
Kwashiorkor *	1 [1]	0 [0]	1 [1]	1 [1]	0 [0]	1 [1]	0 [0]	0 [0]	0 [0]
Severe malnutrition *	0 [0]	1 [1]	1 [1]	0 [0]	1 [1]	1 [1]	0 [0]	0 [0]	0 [0]
**Event rate (all events)**									
Event rate (per 100 person years)	21.3	26.2	23.8	22.6	29.0	25.9	15.5	9.5	12.8
(95% CI)	(12.9, 35.4)	(16.7, 41.0)	(17.0, 33.3)	(13.1, 39.0)	(18.3, 46.0)	(18.2, 36.9)	(3.9, 62.1)	(1.3, 67.5)	(4.1, 39.8)
Unadjusted Rate ratio	0.81	1 (ref)		0.78	1 (ref)		1.63	1 (ref)	
(95% CI)	(0.32, 2.05)	-		(0.30, 2.06)	-		(0.11, 24.75)		
P-value			0.66			0.62			0.72
Adj. Rate ratio**	0.83	1 (ref)							
(95% CI)	(0.33, 2.08)	-							
P-value			0.69						
**Time to first event**									
Unadjusted Hazard ratio	1.06	1 (ref)		1.09	1 (ref)		0.93	1 (ref)	
(95% CI)	(0.46, 2.45)	-		(0.45, 2.62)	-		(0.06, 14.83)	-	
P-value			0.89			0.85			0.96

Adj. hazard ratio**<br>(95% CI)	1.08<br>(0.47, 2.49)	1 (ref)<br>-							
P-value			0.86						
A/B x arm interaction	0.90								

*Resulted in death

**Adjusted for ODYSSEY A and B strata in total.

S11.7 Grade 3 or above clinical and laboratory adverse events to week 96

	Total			A			B		
	**DTG**	**SOC**	**Total**	**DTG**	**SOC**	**Total**	**DTG**	**SOC**	**Total**
**Participants randomised and included**	42	43	85	35	37	72	7	6	13
**Person years**	70	73	143	57	62	120	13	11	23
**Number of events [Number of young people]**	35 [18]	33 [20]	68 [38]	28 [16]	32 [19]	60 [35]	7 [2]	1 [1]	8 [3]
**Biochemical**	4 [4]	3 [2]	7 [6]	3 [3]	3 [2]	6 [5]	1 [1]	0 [0]	1 [1]
Raised ALT	0 [0]	1 [1]	1 [1]	0 [0]	1 [1]	1 [1]	0 [0]	0 [0]	0 [0]
Raised AST	2 [2]	0 [0]	2 [2]	1 [1]	0 [0]	1 [1]	1 [1]	0 [0]	1 [1]
Raised alkaline phosphatase (ALK)	1 [1]	0 [0]	1 [1]	1 [1]	0 [0]	1 [1]	0 [0]	0 [0]	0 [0]
Raised cholesterol	0 [0]	1 [1]	1 [1]	0 [0]	1 [1]	1 [1]	0 [0]	0 [0]	0 [0]
Raised liver enzymes	0 [0]	1 [1]	1 [1]	0 [0]	1 [1]	1 [1]	0 [0]	0 [0]	0 [0]
Raised triglycerides	1 [1]	0 [0]	1 [1]	1 [1]	0 [0]	1 [1]	0 [0]	0 [0]	0 [0]
**Haematological**	11 [9]	12 [11]	23 [20]	9 [7]	12 [11]	21 [18]	2 [2]	0 [0]	2 [2]
Anaemia with no clinical symptoms	6 [6]	8 [8]	14 [14]	5 [5]	8 [8]	13 [13]	1 [1]	0 [0]	1 [1]
Leucopenia	0 [0]	1 [1]	1 [1]	0 [0]	1 [1]	1 [1]	0 [0]	0 [0]	0 [0]
Neutropenia	3 [3]	1 [1]	4 [4]	2 [2]	1 [1]	3 [3]	1 [1]	0 [0]	1 [1]
Thrombocytopenia	2 [2]	2 [2]	4 [4]	2 [2]	2 [2]	4 [4]	0 [0]	0 [0]	0 [0]
**Hepatic**	0 [0]	1 [1]	1 [1]	0 [0]	1 [1]	1 [1]	0 [0]	0 [0]	0 [0]
drug induced liver injury	0 [0]	1 [1]	1 [1]	0 [0]	1 [1]	1 [1]	0 [0]	0 [0]	0 [0]
**Infectious Disease**	15 [11]	15 [11]	30 [22]	12 [9]	14 [10]	26 [19]	3 [2]	1 [1]	4 [3]
Acute diarrhoea not investigated	2 [2]	0 [0]	2 [2]	2 [2]	0 [0]	2 [2]	0 [0]	0 [0]	0 [0]
Acute otitis media	1 [1]	0 [0]	1 [1]	1 [1]	0 [0]	1 [1]	0 [0]	0 [0]	0 [0]
Bronchiolitis	0 [0]	1 [1]	1 [1]	0 [0]	1 [1]	1 [1]	0 [0]	0 [0]	0 [0]
Chest infection	3 [2]	1 [1]	4 [3]	1 [1]	1 [1]	2 [2]	2 [1]	0 [0]	2 [1]
Chronic diarrhoea not investigated	0 [0]	1 [1]	1 [1]	0 [0]	1 [1]	1 [1]	0 [0]	0 [0]	0 [0]
Chronic diarrhoea with no pathogen	1 [1]	0 [0]	1 [1]	1 [1]	0 [0]	1 [1]	0 [0]	0 [0]	0 [0]
Encephalitis - presumed infectious	1 [1]	0 [0]	1 [1]	1 [1]	0 [0]	1 [1]	0 [0]	0 [0]	0 [0]
Gastroenteritis	1 [1]	0 [0]	1 [1]	1 [1]	0 [0]	1 [1]	0 [0]	0 [0]	0 [0]
Gastroenteritis *	0 [0]	1 [1]	1 [1]	0 [0]	0 [0]	0 [0]	0 [0]	1 [1]	1 [1]
Hepatitis A	1 [1]	0 [0]	1 [1]	0 [0]	0 [0]	0 [0]	1 [1]	0 [0]	1 [1]
Internal abscess	0 [0]	1 [1]	1 [1]	0 [0]	1 [1]	1 [1]	0 [0]	0 [0]	0 [0]
Measles	1 [1]	1 [1]	2 [2]	1 [1]	1 [1]	2 [2]	0 [0]	0 [0]	0 [0]
P falciparum malaria	1 [1]	0 [0]	1 [1]	1 [1]	0 [0]	1 [1]	0 [0]	0 [0]	0 [0]
Pneumonia - other bacterial	0 [0]	1 [1]	1 [1]	0 [0]	1 [1]	1 [1]	0 [0]	0 [0]	0 [0]
Pneumonia no organism identified, aspiration pneumonia	2 [2]	0 [0]	2 [2]	2 [2]	0 [0]	2 [2]	0 [0]	0 [0]	0 [0]
Pneumonia no organism identified, aspiration pneumonia *	0 [0]	1 [1]	1 [1]	0 [0]	1 [1]	1 [1]	0 [0]	0 [0]	0 [0]
Presumed septicaemia/bacteremia - no organism	0 [0]	1 [1]	1 [1]	0 [0]	1 [1]	1 [1]	0 [0]	0 [0]	0 [0]
Pyogenic meningitis - organism	0 [0]	1 [1]	1 [1]	0 [0]	1 [1]	1 [1]	0 [0]	0 [0]	0 [0]
Tuberculosis - abdominal	0 [0]	1 [1]	1 [1]	0 [0]	1 [1]	1 [1]	0 [0]	0 [0]	0 [0]
Tuberculosis - disseminated/miliary	0 [0]	1 [1]	1 [1]	0 [0]	1 [1]	1 [1]	0 [0]	0 [0]	0 [0]
Tuberculosis - pulmonary - smear negative or not done	1 [1]	2 [2]	3 [3]	1 [1]	2 [2]	3 [3]	0 [0]	0 [0]	0 [0]
Tuberculosis - pulmonary - smear negative or not done *	0 [0]	1 [1]	1 [1]	0 [0]	1 [1]	1 [1]	0 [0]	0 [0]	0 [0]
**Non HIV related deaths**	1 [1]	0 [0]	1 [1]	1 [1]	0 [0]	1 [1]	0 [0]	0 [0]	0 [0]
Traumatic *	1 [1]	0 [0]	1 [1]	1 [1]	0 [0]	1 [1]	0 [0]	0 [0]	0 [0]
**Systemic**	4 [4]	2 [2]	6 [6]	3 [3]	2 [2]	5 [5]	1 [1]	0 [0]	1 [1]
Dehydration	1 [1]	0 [0]	1 [1]	1 [1]	0 [0]	1 [1]	0 [0]	0 [0]	0 [0]
Kwashiorkor	1 [1]	0 [0]	1 [1]	1 [1]	0 [0]	1 [1]	0 [0]	0 [0]	0 [0]
Kwashiorkor *	1 [1]	0 [0]	1 [1]	1 [1]	0 [0]	1 [1]	0 [0]	0 [0]	0 [0]
Severe malnutrition	1 [1]	0 [0]	1 [1]	0 [0]	0 [0]	0 [0]	1 [1]	0 [0]	1 [1]
Severe malnutrition *	0 [0]	2 [2]	2 [2]	0 [0]	2 [2]	2 [2]	0 [0]	0 [0]	0 [0]
**Event rate (all events)**									
Event rate (per 100 person years)	49.8	45.5	47.6	48.7	51.5	50.2	54.4	9.5	34.2

(95% CI)	(35.7, 69.3)	(32.3, 63.9)	(37.5, 60.3)	(33.6, 70.6)	(36.4, 72.9)	(39.0, 64.6)	(25.9, 114.0)	(1.3, 67.5)	(17.1, 68.4)
Unadjusted Rate ratio	1.09	1 (ref)		0.95	1 (ref)		5.71	1 (ref)	
(95% CI)	(0.59, 2.02)	-		(0.50, 1.77)	-		(0.57, 57.77)	-	
P-value			0.77			0.86			0.14
Adj. Rate ratio**	1.11	1 (ref)							
(95% CI)	(0.61, 2.03)	-							
P-value			0.74						
**Time to first event**									
Unadjusted Hazard ratio	0.93	1 (ref)		0.88	1 (ref)		2.03	1 (ref)	
(95% CI)	(0.49, 1.75)	-		(0.45, 1.71)	-		(0.18, 22.45)	-	
P-value			0.82			0.71			0.57
Adj. hazard ratio**	0.94	1 (ref)							
(95% CI)	(0.50, 1.77)	-							
P-value			0.84						
A/B x arm interaction	0.54								

*Resulted in death

**Adjusted for ODYSSEY A and B strata in total.

S11.8 Adverse events leading to ART modification (any grade) to week 96

	Total			A			B		
	**DTG**	**SOC**	**Total**	**DTG**	**SOC**	**Total**	**DTG**	**SOC**	**Total**
**Participants randomised and included**	42	43	85	35	37	72	7	6	13
**Person years**	70	73	143	57	62	120	13	11	23
**Number of events [Number of young people+]**	0 [0]	1 [1]	1 [1]	0 [0]	1 [1]	1 [1]	0 [0]	0 [0]	0 [0]
**Biochemical**	0 [0]	1 [1]	1 [1]	0 [0]	1 [1]	1 [1]	0 [0]	0 [0]	0 [0]
Raised liver enzymes	0 [0]	1 [1]	1 [1]	0 [0]	1 [1]	1 [1]	0 [0]	0 [0]	0 [0]

+ Raised Liver Enzymes - Event is considered ART modifying as the event led to the participant stopping their ART regimen. The participant did subsequently re-start the same ART and hence is not contained within the table of treatment changes.

S12 Patient Reported Outcomes

Note: ODYSSEY B is not presented here due to having few participants

S12.1 Summary of carer reported adherence

	Total						A					
	DTG		SOC		Total		DTG		SOC		Total	
**Participants randomised**	42		43		85		35		37		72	
**Dose missed in the last week #missed [#questionnaires completed]**												
Week 4	3	[40]	5	[38]	8	[78]	3	[33]	4	[32]	7	[65]
Week 12	4	[40]	5	[38]	9	[78]	4	[33]	4	[33]	8	[66]
Week 24	2	[38]	4	[39]	6	[77]	1	[31]	3	[33]	4	[64]
Week 36	5	[36]	6	[40]	11	[76]	4	[29]	6	[34]	10	[63]
Week 48	3	[34]	7	[36]	10	[70]	3	[27]	7	[31]	10	[58]
Week 60	4	[35]	2	[34]	6	[69]	3	[28]	2	[28]	5	[56]
Week 72	4	[35]	5	[35]	9	[70]	3	[28]	4	[30]	7	[58]
Week 84	2	[34]	3	[33]	5	[67]	1	[27]	3	[29]	4	[56]
Week 96	4	[34]	6	[38]	10	[72]	2	[27]	6	[33]	8	[60]
Week 108	5	[33]	4	[30]	9	[63]	4	[26]	3	[26]	7	[52]
Week 120	4	[25]	3	[22]	7	[47]	1	[21]	3	[18]	4	[39]
Week 132	1	[14]	3	[14]	4	[28]	1	[11]	2	[11]	3	[22]
Total # of reports of dose missed in the last week over follow-up (% of all follow-up questionnaires)	42	(10%)	53	(13%)	95	(12%)	31	(10%)	47	(14%)	78	(12%)
# of participants reporting at >=1 follow-up visit	25	(61%)	30	(73%)	55	(67%)	21	(62%)	25	(71%)	46	(67%)
P-value*					0.50						0.49	
**Dose missed in the last month #missed [#questionnaires completed]**												
Week 4	2	[40]	3	[38]	5	[78]	2	[33]	2	[32]	4	[65]
Week 12	3	[39]	8	[39]	11	[78]	2	[32]	7	[33]	9	[65]
Week 24	4	[38]	4	[39]	8	[77]	2	[31]	4	[33]	6	[64]
Week 36	4	[36]	5	[40]	9	[76]	4	[29]	4	[34]	8	[63]
Week 48	3	[34]	7	[36]	10	[70]	3	[27]	7	[31]	10	[58]
Week 60	5	[35]	3	[34]	8	[69]	4	[28]	3	[28]	7	[56]
Week 72	5	[35]	6	[35]	11	[70]	4	[28]	6	[30]	10	[58]
Week 84	4	[33]	3	[33]	7	[66]	4	[27]	3	[29]	7	[56]
Week 96	3	[35]	5	[38]	8	[73]	3	[28]	5	[33]	8	[61]
Week 108	3	[33]	6	[30]	9	[63]	3	[26]	5	[26]	8	[52]
Week 120	2	[25]	4	[22]	6	[47]	1	[21]	3	[18]	4	[39]
Week 132	1	[14]	0	[14]	1	[28]	1	[11]	0	[11]	1	[22]
Total # of reports of dose missed in the last week over follow-up (% of all follow-up questionnaires)	39	(10%)	55	(14%)	94	(12%)	33	(10%)	50	(14%)	83	(12%)
# of participants reporting at >=1 follow-up visit	22	(54%)	26	(63%)	48	(59%)	18	(53%)	22	(63%)	40	(58%)
P-value*					0.27						0.35	

*Mixed logistic regression model with a random effect for participant and fixed effects for treatment arm, visit week and ODYSSEY A/B (total population only).

S12.2 Summary of carer reported acceptability of treatment during follow-up

	Total						A					
	DTG		SOC		Total		DTG		SOC		Total	
Participants randomised	42		43		85		35		37		72	
**Problems with taste/does not like taste #with a problem [#questionnaires completed]**												
Week 4	5	[35]	6	[32]	11	[67]	5	[29]	6	[27]	11	[56]
Week 12	5	[34]	8	[32]	13	[66]	5	[27]	8	[27]	13	[54]
Week 24	3	[35]	3	[34]	6	[69]	3	[28]	3	[29]	6	[57]
Week 48	3	[30]	3	[31]	6	[61]	3	[23]	3	[25]	6	[48]
Week 72	3	[32]	4	[33]	7	[65]	3	[26]	4	[28]	7	[54]
Week 96	4	[33]	2	[37]	6	[70]	3	[27]	2	[32]	5	[59]
Week 120	1	[24]	1	[19]	2	[43]	1	[19]	1	[15]	2	[34]
Total # of reports of problems over follow-up (% of all follow-up questionnaires)	24	(10%)	28	(12%)	52	(11%)	23	(13%)	28	(15%)	51	(14%)
# of participants reporting at >=1 follow-up visit	13	(32%)	14	(34%)	27	(33%)	12	(35%)	14	(40%)	26	(38%)
P-value*					0.78						0.62	
**Problems with swallowing/not easy to swallow #with a problem [#questionnaires completed]**												
Week 4	3	[35]	6	[32]	9	[67]	3	[29]	6	[27]	9	[56]
Week 12	4	[34]	4	[32]	8	[66]	4	[27]	4	[27]	8	[54]
Week 24	2	[35]	2	[34]	4	[69]	2	[28]	2	[29]	4	[57]
Week 48	2	[30]	0	[31]	2	[61]	2	[23]	0	[25]	2	[48]
Week 72	2	[32]	1	[33]	3	[65]	2	[26]	1	[28]	3	[54]
Week 96	0	[33]	2	[35]	2	[68]	0	[27]	1	[30]	1	[57]
Week 120	0	[24]	0	[18]	0	[42]	0	[19]	0	[14]	0	[33]
Total # of reports of problems over follow-up (% of all follow-up questionnaires)	13	(6%)	17	(8%)	30	(7%)	13	(7%)	16	(9%)	29	(8%)
# of participants reporting at >=1 follow-up visit	10	(24%)	13	(32%)	23	(28%)	10	(29%)	12	(34%)	22	(32%)
P-value*					0.51						0.68	

*Mixed logistic regression model with a random effect for participant and fixed effects for treatment arm, visit week and ODYSSEY A/B (total population only).

S13 Antiretroviral Therapy

S13.1 Summary of initial SOC treatment regimens

		Total		A		B	
		SOC		SOC		SOC	
**Participants randomised**		43		37		6	
**ART regimen**							
**Non-NRTI component**	NRTI component						
EFV	ABC 3TC	4	(9%)	4	(11%)	0	(%)
LOP	ABC 3TC	30	(70%)	29	(78%)	1	(17%)
LOP	ZDV 3TC	2	(5%)	0	(0%)	2	(33%)
NVP	ABC 3TC	3	(7%)	3	(8%)	0	(%)
NVP	ZDV 3TC	2	(5%)	1	(3%)	1	(17%)
RAL	ZDV 3TC	2	(5%)	0	(0%)	2	(33%)

S13.2 Summary of initial DTG regimens

		Total		A		B	
		DTG		DTG		DTG	
**Participants randomised**		42		35		7	
**ART regimen**							
**Non-NRTI component**	NRTI component						
DTG	ABC 3TC	38	(90%)	35	(100%)	3	(43%)
DTG	ZDV 3TC	4	(10%)			4	(57%)

S13.3 Definitions of Treatment Regimens

1st ODYSSEY Regimen: Can either be strictly the regimen allocated at trial entry or initial regimen allowing for NRTI substitutions or reductions for any reason and/or third agent substitutions for reasons other than toxicity, treatment failure, pregnancy or a major protocol deviation (these include simplification, drug availability, patient/carer decision and increases in weight).

2nd ODYSSEY Regimen: Participants will considered to be on their second ODYSSEY regimen if changed the third agent in initial regimen for reasons of toxicity, treatment failure, pregnancy or a major protocol deviation.

3rd ODYSSEY Regimen: Participants will considered to be on their third ODYSSEY regimen if changed the third agent in their second regimen for reasons of toxicity, treatment failure, pregnancy or a major protocol deviation.

S13.4 Treatment regimen at last visit

	Total			A			B		
	**DTG**	**SOC**	**Total**	**DTG**	**SOC**	**Total**	**DTG**	**SOC**	**Total**
**Participants randomised**	42	43	85	35	37	72	7	6	13
**ODYSSEY regimen at last visit, number of participants (%):**									
1st:strict initial	41 (98%)	37 (86%)	78 (92%)	34 (97%)	32 (86%)	66 (92%)	7 (100%)	5 (83%)	12 (92%)
1st:NRTI substitution/reduction	0 (0%)	1 (2%)	1 (1%)	0 (0%)	1 (3%)	1 (1%)	0 (0%)	0 (0%)	0 (0%)
1st:substitution 3rd agent	0 (0%)	1 (2%)	1 (1%)	0 (0%)	1 (3%)	1 (1%)	0 (0%)	0 (0%)	0 (0%)
2nd regimen	0 (0%)	4 (9%)	4 (5%)	0 (0%)	3 (8%)	3 (4%)	0 (0%)	1 (17%)	1 (8%)
Stopped	1 (2%)	0 (0%)	1 (1%)	1 (3%)	0 (0%)	1 (1%)	0 (0%)	0 (0%)	0 (0%)

The following two tables present all ART changes from strict initial regimen and stops of >31 days. Stops of ART of <=31 days are ignored here.

S13.5 Details of Changes to Treatment Regimens for Toxicity or Treatment Failure

ID	Arm	Treatment	Date of Change	Week	ART Regimen	Treatment Line	Reason for Change
5	A	SOC	15nov2018	-	ABC 3TC LOP	1st:Strict initial	Start ODYSSEY Trial
5	A	SOC	18feb2020	65	ZDV 3TC LOP	1st:NRTI substitution/reduction	Treatment failure
6	A	SOC	19feb2019	-	ABC 3TC NVP	1st:Strict initial	Start ODYSSEY Trial
6	A	SOC	28feb2020	53	ABC 3TC EFV	1st:substitution 3rd agent	TB
6	A	SOC	12oct2020	85	ZDV 3TC LOP	2nd: treatment failure	Treatment failure
7	A	SOC	16aug2018	-	ABC 3TC EFV	1st:Strict initial	Start ODYSSEY Trial
7	A	SOC	25feb2021	132	ABC 3TC LOP	1st:substitution 3rd agent	Change in National Guidelines
7	A	SOC	03mar2021	132	ABC 3TC EFV	2nd: toxicity	Adverse event
8	A	SOC	24jan2019	-	ABC 3TC LOP	1st:Strict initial	Start ODYSSEY Trial
8	A	SOC	25jun2020	74	ZDV 3TC RAL	2nd: treatment failure	Treatment failure
9	A	SOC	07jun2019	-	ABC 3TC LOP	1st:Strict initial	Start ODYSSEY Trial
9	A	SOC	22jun2019	2	-	stopped	Adverse event
9	A	SOC	27aug2019	11	ABC 3TC LOP	1st:Strict initial	Restart previous ART
10	A	SOC	26aug2019	-	ABC 3TC LOP	1st:Strict initial	Start ODYSSEY Trial
10	A	SOC	01nov2019	9	-	stopped	Adverse event
10	A	SOC	08feb2020	23	ABC 3TC LOP	1st:Strict initial	Restart previous ART
13	B	SOC	06nov2018	-	ZDV 3TC NVP	1st:Strict initial	Start ODYSSEY Trial
13	B	SOC	01sep2020	95	ZDV 3TC LOP	2nd: treatment failure	Treatment failure

ID 7 – Participant switched from efavrenz to lopinavir due to a change in national guidelines and subsequently changed back to their initial regimen of efavrenz due to an adverse event and are hence considered to be on their second regimen

S13.6 Details of Other Changes to Treatment Regimens

ID	Arm	Treatment	Date of Change	Week	ART Regimen	Treatment Line	Reason for Change
1	A	DTG	12jul2018	-	ABC 3TC DTG	1st:Strict initial	Start ODYSSEY Trial
1	A	DTG	07aug2018	3	-	stopped	Missed/Forgot/Compliance
2	A	DTG	15jan2019	-	ABC 3TC DTG	1st:Strict initial	Start ODYSSEY Trial
2	A	DTG	16dec2019	47	-	stopped	Patient/carer decision
2	A	DTG	12mar2020	60	ABC 3TC DTG	1st:Strict initial	Restart previous ART
3	A	DTG	30jan2019	-	ABC 3TC DTG	1st:Strict initial	Start ODYSSEY Trial
3	A	DTG	19jun2020	72	-	stopped	Unable/failed to attend clinic
3	A	DTG	27jul2020	77	ABC 3TC DTG	1st:Strict initial	Unable/failed to attend clinic
4	A	DTG	13feb2019	-	ABC 3TC DTG	1st:Strict initial	Start ODYSSEY Trial
4	A	DTG	24oct2019	36	-	stopped	Unable/failed to attend clinic
4	A	DTG	07sep2020	81	ABC 3TC DTG	1st:Strict initial	Unable/failed to attend clinic
11	A	SOC	25jul2018	-	ZDV 3TC NVP	1st:Strict initial	Start ODYSSEY Trial
11	A	SOC	11dec2019	72	ZDV 3TC DTG	1st:substitution 3rd agent	TB
12	A	SOC	31jul2018	-	ABC 3TC EFV	1st:Strict initial	Start ODYSSEY Trial
12	A	SOC	15aug2018	2	-	stopped	Unable/failed to attend clinic
12	A	SOC	22oct2018	11	ABC 3TC EFV	1st:Strict initial	Restart previous ART

ID 1 - participant was considered as stopped ART at their last visit, they subsequently withdrew

S14 Anthropometric measures

Note: ODYSSEY B is not presented here due to having few participants

Throughout this section, n refers to the number of participants with available measurement at each visit week. Numbers in models may be lower where there are missing data at baseline.

S14.1 Total population - Height (cm) change over follow-up

Weeks since randomisation	DTG			SOC			Unadjusted Difference*				Adjusted Difference**				Interaction++
	n	mean	(SE)	n	mean	(SE)	mean	(SE)	[95% CI]	p	mean	(SE)	[95% CI]	p	p
12	39	3.2	(0.3)	40	2.6	(0.3)	0.6	(0.4)	[-0.2, 1.3]	0.12	0.6	(0.4)	[-0.2, 1.3]	0.13	0.70
24	39	6.0	(0.4)	40	5.2	(0.4)	0.8	(0.5)	[-0.3, 1.8]	0.17	0.7	(0.5)	[-0.4, 1.8]	0.18	0.081
36	35	8.1	(0.5)	39	7.1	(0.4)	1.0	(0.6)	[-0.3, 2.3]	0.11	1.0	(0.6)	[-0.3, 2.3]	0.13	0.16
48	35	10.3	(0.5)	39	9.1	(0.5)	1.2	(0.8)	[-0.3, 2.7]	0.11	1.2	(0.8)	[-0.3, 2.7]	0.12	0.25
60	33	12.1	(0.6)	36	11.8	(0.5)	0.3	(0.8)	[-1.3, 1.9]	0.71	0.3	(0.8)	[-1.3, 1.9]	0.73	0.27
72	32	13.7	(0.6)	33	14.2	(0.6)	-0.5	(0.9)	[-2.3, 1.2]	0.54	-0.5	(0.9)	[-2.3, 1.3]	0.56	0.46
84	35	16.4	(0.7)	37	15.7	(0.7)	0.7	(1.0)	[-1.3, 2.6]	0.49	0.7	(1.0)	[-1.3, 2.7]	0.48	0.17
96	32	18.8	(0.7)	36	18.5	(0.7)	0.4	(1.0)	[-1.6, 2.4]	0.72	0.4	(1.0)	[-1.7, 2.4]	0.71	0.33
Average treatment differences through follow-up+							0.5	(0.6)	[-0.6, 1.7]	0.37	0.5	(0.6)	[-0.6, 1.7]	0.38	0.12

S14.2 ODYSSEY A - Height (cm) change over follow-up

Weeks since randomisation	DTG			SOC			Unadjusted Difference*			
	n	mean	(SE)	n	mean	(SE)	mean	(SE)	[95% CI]	p
12	32	3.4	(0.3)	34	2.8	(0.3)	0.6	(0.4)	[-0.2, 1.4]	0.13
24	32	6.5	(0.4)	34	5.3	(0.4)	1.2	(0.6)	[0.0, 2.3]	0.050
36	28	8.6	(0.5)	33	7.2	(0.5)	1.4	(0.7)	[-0.0, 2.9]	0.058
48	28	10.9	(0.6)	33	9.3	(0.6)	1.6	(0.8)	[-0.1, 3.2]	0.065
60	26	12.8	(0.6)	30	12.1	(0.6)	0.7	(0.9)	[-1.1, 2.5]	0.44
72	25	14.4	(0.7)	28	14.7	(0.7)	-0.2	(1.0)	[-2.2, 1.8]	0.82
84	28	17.3	(0.8)	33	16.0	(0.8)	1.3	(1.1)	[-1.0, 3.5]	0.26
96	26	19.7	(0.8)	32	19.0	(0.8)	0.8	(1.1)	[-1.5, 3.1]	0.50
Average treatment differences through follow-up+							0.9	(0.7)	[-0.4, 2.2]	0.16

*Change calculated using normal regression adjusting for baseline only. Presenting mean change from a baseline of 71.9 in total and 69.8 in A.
**Normal regression adjusting for baseline and ODYSSEY A/B strata.
+Linear mixed models fitted with random intercept and fixed effects for treatment group, study visit and adjustment covariates (baseline Height (cm) and ODYSSEY A/B strata (total population only))
++ Test of heterogeneity of treatment effect between ODYSSEY A/B

S14.3 Total population - Height (cm) change over follow-up

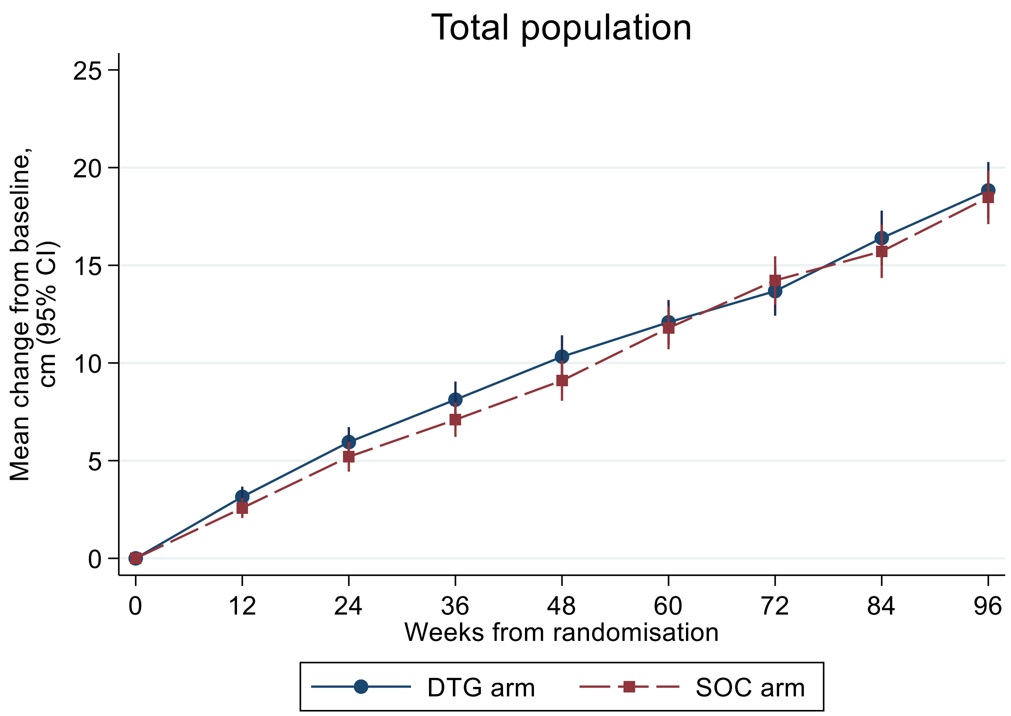

S14.4 ODYSSEY A - Height (cm) change over follow-up

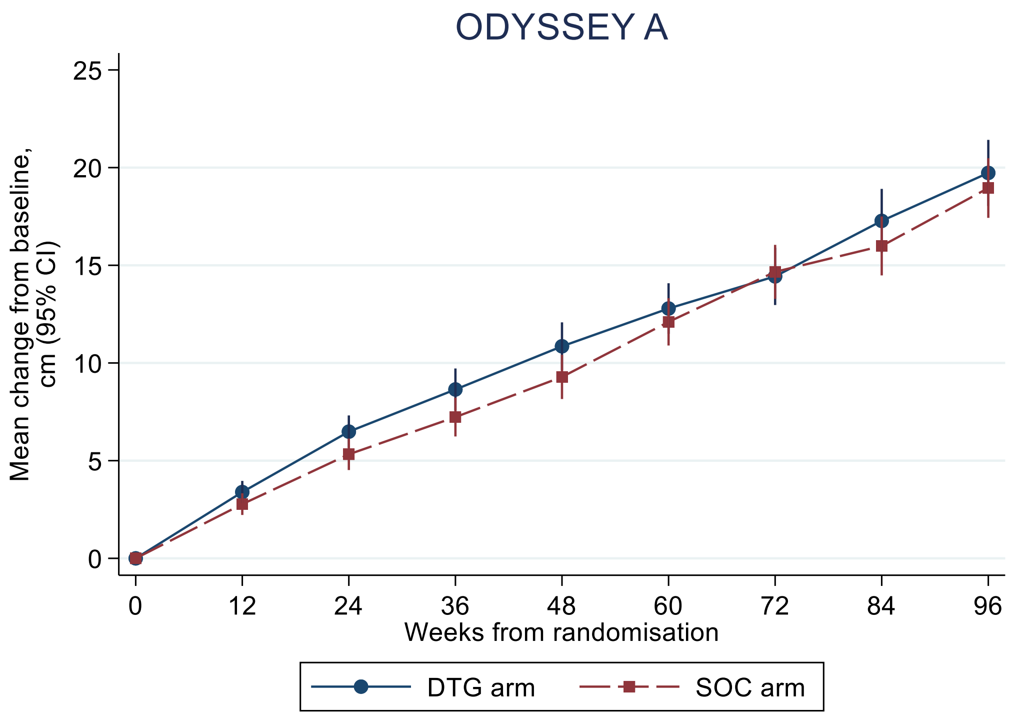

S14.5 Total population - Weight (kg) change over follow-up

Weeks since randomisation	DTG			SOC			Unadjusted Difference*				Adjusted Difference**				Interaction++
	n	mean	(SE)	n	mean	(SE)	mean	(SE)	[95% CI]	p	mean	(SE)	[95% CI]	p	p
12	39	0.97	(0.14)	40	1.12	(0.14)	-0.15	(0.19)	[-0.53, 0.24]	0.45	-0.14	(0.20)	[-0.53, 0.25]	0.47	0.75
24	39	1.89	(0.16)	40	1.73	(0.16)	0.16	(0.23)	[-0.29, 0.62]	0.48	0.16	(0.23)	[-0.30, 0.62]	0.48	0.37
36	36	2.43	(0.20)	40	2.42	(0.19)	0.01	(0.27)	[-0.53, 0.54]	0.98	0.01	(0.27)	[-0.53, 0.55]	0.96	0.94
48	35	3.18	(0.21)	40	2.95	(0.20)	0.23	(0.29)	[-0.35, 0.82]	0.42	0.26	(0.29)	[-0.32, 0.85]	0.37	0.98
60	34	3.56	(0.24)	37	3.62	(0.23)	-0.06	(0.33)	[-0.71, 0.59]	0.86	-0.05	(0.33)	[-0.70, 0.61]	0.89	0.84
72	33	4.19	(0.27)	33	4.25	(0.27)	-0.05	(0.39)	[-0.83, 0.72]	0.89	-0.00	(0.39)	[-0.78, 0.78]	1	0.52
84	36	4.57	(0.24)	37	4.70	(0.23)	-0.13	(0.33)	[-0.79, 0.53]	0.70	-0.09	(0.34)	[-0.76, 0.59]	0.80	0.55
96	35	4.96	(0.24)	36	5.14	(0.24)	-0.18	(0.34)	[-0.86, 0.49]	0.60	-0.15	(0.34)	[-0.83, 0.54]	0.67	0.71
Average treatment differences through follow-up+							-0.01	(0.24)	[-0.48, 0.46]	0.97	0.00	(0.24)	[-0.47, 0.48]	0.99	0.87

S14.6 ODYSSEY A - Weight (kg) change over follow-up

Weeks since randomisation	DTG			SOC			Unadjusted Difference*			
	n	mean	(SE)	n	mean	(SE)	mean	(SE)	[95% CI]	p
12	32	1.06	(0.14)	34	1.22	(0.14)	-0.16	(0.20)	[-0.57, 0.24]	0.42
24	32	1.95	(0.17)	34	1.87	(0.17)	0.08	(0.24)	[-0.41, 0.57]	0.75
36	29	2.57	(0.21)	34	2.54	(0.19)	0.03	(0.28)	[-0.54, 0.60]	0.91
48	28	3.39	(0.24)	34	3.12	(0.22)	0.27	(0.32)	[-0.37, 0.91]	0.40
60	27	3.75	(0.26)	31	3.76	(0.24)	-0.00	(0.35)	[-0.71, 0.70]	0.99
72	26	4.56	(0.31)	28	4.43	(0.30)	0.13	(0.43)	[-0.74, 1.00]	0.76
84	29	4.88	(0.26)	33	4.87	(0.24)	0.01	(0.35)	[-0.70, 0.72]	0.98
96	29	5.24	(0.27)	32	5.32	(0.25)	-0.08	(0.37)	[-0.82, 0.66]	0.83
Average treatment differences through follow-up+							0.03	(0.25)	[-0.47, 0.53]	0.90

*Change calculated using normal regression adjusting for baseline only. Presenting mean change from a baseline of 8.1 in total and 7.6 in A.

**Normal regression adjusting for baseline and ODYSSEY A/B strata.

+Linear mixed models fitted with random intercept and fixed effects for treatment group, study visit and adjustment covariates (baseline Weight (kg) and ODYSSEY A/B strata (total population only))

++ Test of heterogeneity of treatment effect between ODYSSEY A/B

S14.7 Total population - Weight (kg) change over follow-up

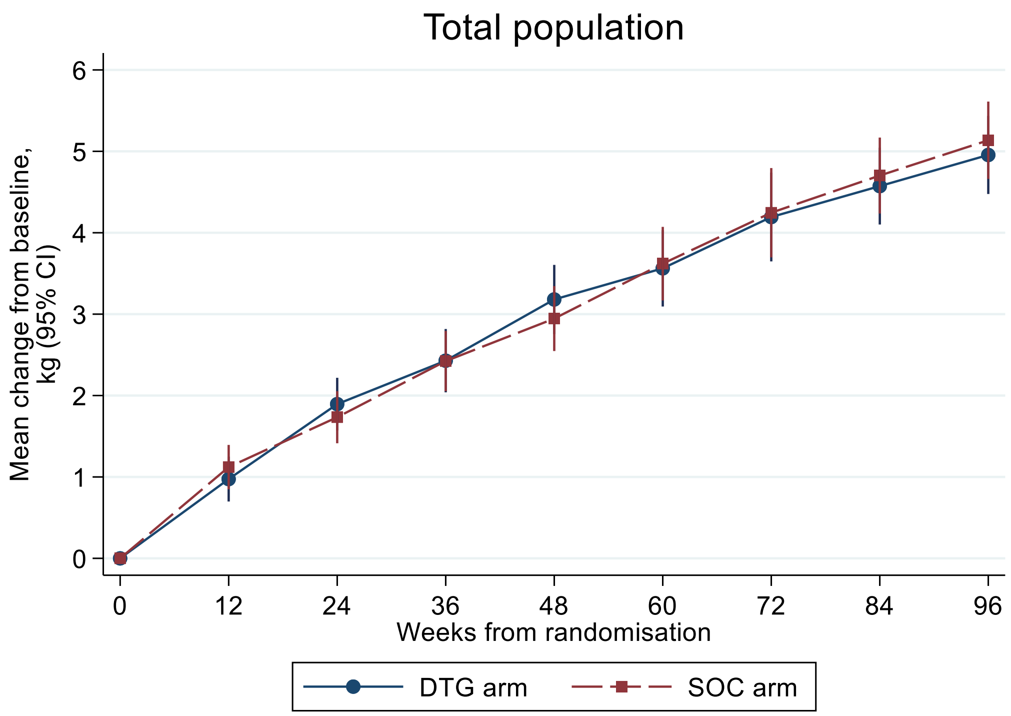

S14.8 ODYSSEY A - Weight (kg) change over follow-up

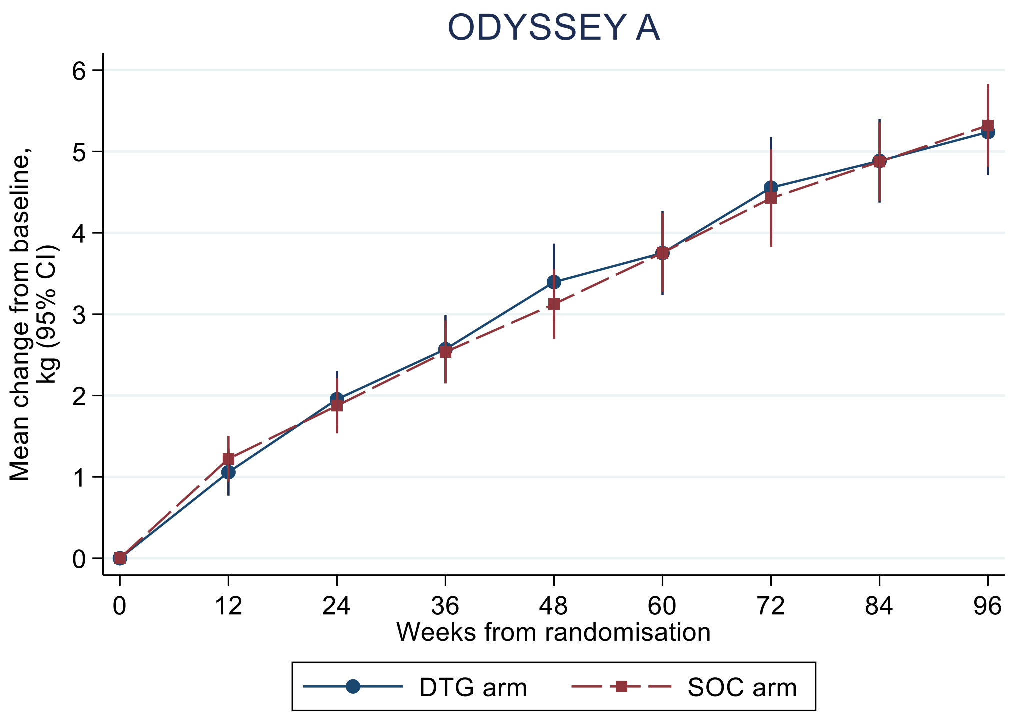

S14.9 Total population - BMI-for-age change over follow-up

Weeks since randomisation	DTG			SOC			Unadjusted Difference*				Adjusted Difference**				Interaction++
	n	mean	(SE)	n	mean	(SE)	mean	(SE)	[95% CI]	p	mean	(SE)	[95% CI]	p	p
12	39	0.5	(0.2)	40	1.0	(0.2)	-0.5	(0.3)	[-1.0, 0.1]	0.097	-0.4	(0.3)	[-1.0, 0.1]	0.10	0.42
24	39	1.0	(0.3)	40	1.1	(0.3)	-0.1	(0.4)	[-0.8, 0.6]	0.78	-0.1	(0.4)	[-0.8, 0.6]	0.79	0.64
36	35	1.0	(0.3)	39	1.4	(0.2)	-0.4	(0.4)	[-1.2, 0.3]	0.22	-0.4	(0.4)	[-1.1, 0.3]	0.25	0.90
48	35	1.3	(0.3)	39	1.6	(0.3)	-0.2	(0.4)	[-1.0, 0.5]	0.55	-0.2	(0.4)	[-0.9, 0.6]	0.64	0.81
60	33	1.4	(0.3)	36	1.5	(0.3)	-0.1	(0.4)	[-0.9, 0.7]	0.78	-0.1	(0.4)	[-0.8, 0.7]	0.83	0.42
72	32	1.7	(0.3)	33	1.8	(0.3)	-0.2	(0.4)	[-1.0, 0.7]	0.70	-0.1	(0.4)	[-0.9, 0.8]	0.86	0.20
84	35	1.4	(0.3)	37	1.8	(0.3)	-0.4	(0.4)	[-1.2, 0.4]	0.30	-0.3	(0.4)	[-1.1, 0.5]	0.42	0.33
96	32	1.1	(0.3)	36	1.5	(0.3)	-0.3	(0.4)	[-1.2, 0.5]	0.41	-0.3	(0.4)	[-1.1, 0.5]	0.5	0.45
Average treatment differences through follow-up+							-0.2	(0.3)	[-0.8, 0.4]	0.46	-0.2	(0.3)	[-0.8, 0.4]	0.50	0.60

S14.10 ODYSSEY A - BMI-for-age change over follow-up

Weeks since randomisation	DTG			SOC			Unadjusted Difference*			
	n	mean	(SE)	n	mean	(SE)	mean	(SE)	[95% CI]	p
12	32	0.70	(0.22)	34	1.05	(0.21)	-0.35	(0.30)	[-0.96, 0.26]	0.25
24	32	1.04	(0.28)	34	1.20	(0.28)	-0.17	(0.40)	[-0.96, 0.62]	0.67
36	28	1.14	(0.30)	33	1.54	(0.28)	-0.40	(0.41)	[-1.22, 0.41]	0.33
48	28	1.63	(0.31)	33	1.76	(0.28)	-0.14	(0.42)	[-0.97, 0.70]	0.75
60	26	1.74	(0.32)	30	1.67	(0.30)	0.07	(0.44)	[-0.81, 0.95]	0.87
72	25	2.15	(0.35)	28	1.99	(0.33)	0.16	(0.48)	[-0.80, 1.13]	0.73
84	28	1.80	(0.33)	33	1.97	(0.31)	-0.17	(0.45)	[-1.07, 0.73]	0.71
96	26	1.43	(0.34)	32	1.59	(0.31)	-0.15	(0.46)	[-1.07, 0.76]	0.74
Average treatment differences through follow-up+							-0.13	(0.33)	[-0.78, 0.52]	0.70

*Change calculated using normal regression adjusting for baseline only. Presenting mean change from a baseline of -0.9 in total and -1.0 in A.
**Normal regression adjusting for baseline and ODYSSEY A/B strata.
+Linear mixed models fitted with random intercept and fixed effects for treatment group, study visit and adjustment covariates (baseline BMI-for-age and ODYSSEY A/B strata (total population only))
++ Test of heterogeneity of treatment effect between ODYSSEY A/B

S14.11 Total population - BMI-for-age change over follow-up

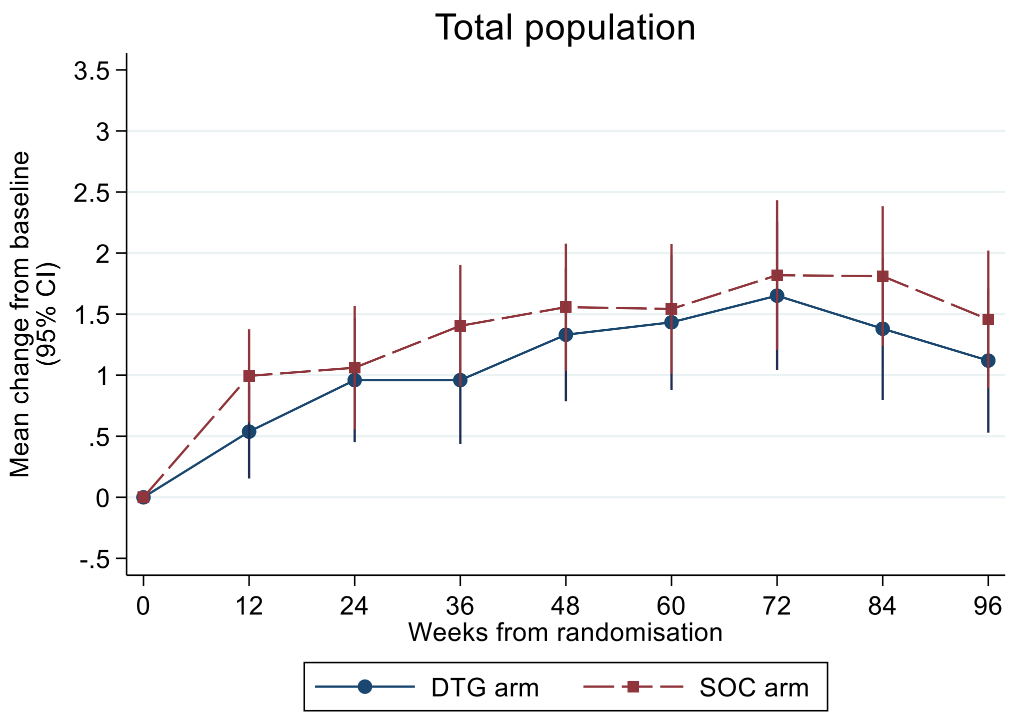

S14.12 ODYSSEY A - BMI-for-age change over follow-up

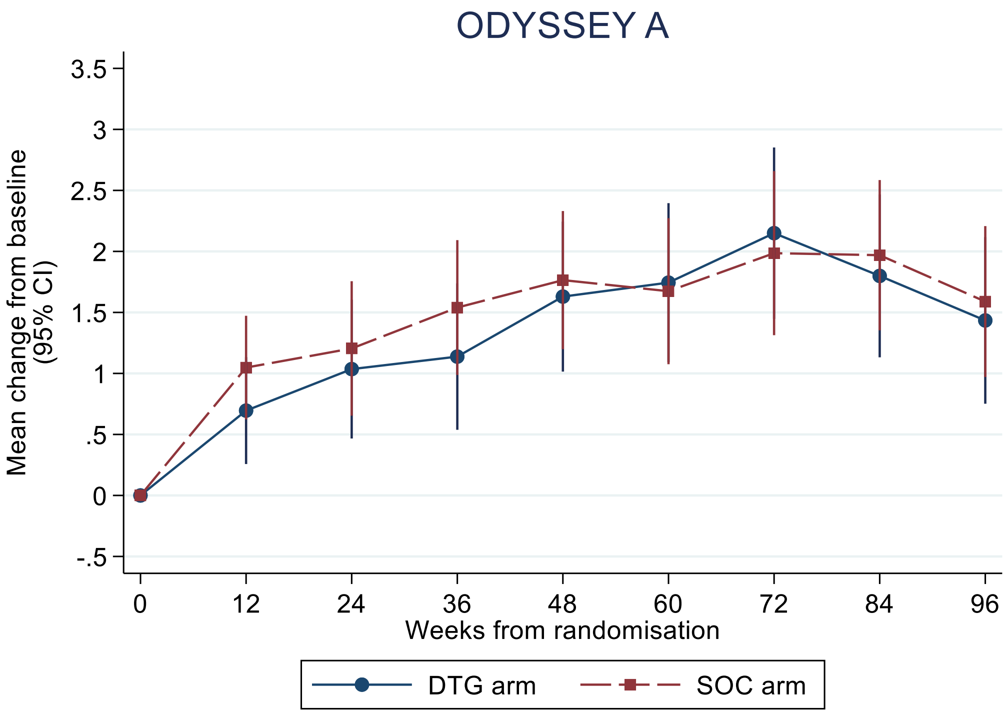

S14.13 Total population - Weight-for-age change over follow-up

Weeks since randomisation	DTG			SOC			Unadjusted Difference*				Adjusted Difference**				Interaction++
	n	mean	(SE)	n	mean	(SE)	mean	(SE)	[95% CI]	p	mean	(SE)	[95% CI]	p	p
12	39	0.44	(0.15)	40	0.54	(0.15)	-0.10	(0.21)	[-0.51, 0.31]	0.64	-0.09	(0.21)	[-0.50, 0.33]	0.68	0.82
24	39	0.83	(0.17)	40	0.61	(0.17)	0.22	(0.24)	[-0.25, 0.69]	0.36	0.23	(0.24)	[-0.25, 0.70]	0.34	0.56
36	36	0.88	(0.17)	40	0.80	(0.16)	0.09	(0.23)	[-0.38, 0.55]	0.71	0.10	(0.23)	[-0.37, 0.56]	0.67	0.99
48	35	1.09	(0.17)	40	0.87	(0.17)	0.23	(0.24)	[-0.25, 0.71]	0.35	0.25	(0.24)	[-0.22, 0.73]	0.29	1
60	34	1.05	(0.17)	37	1.03	(0.16)	0.02	(0.24)	[-0.45, 0.49]	0.93	0.04	(0.24)	[-0.43, 0.51]	0.87	0.85
72	33	1.18	(0.18)	33	1.20	(0.19)	-0.02	(0.26)	[-0.55, 0.50]	0.93	0.03	(0.26)	[-0.49, 0.54]	0.92	0.51
84	36	1.17	(0.16)	37	1.21	(0.16)	-0.04	(0.23)	[-0.50, 0.42]	0.86	0.02	(0.23)	[-0.44, 0.48]	0.94	0.56
96	35	1.16	(0.16)	36	1.20	(0.16)	-0.03	(0.23)	[-0.49, 0.42]	0.88	0.00	(0.23)	[-0.45, 0.45]	1	0.82
Average treatment differences through follow-up+							0.06	(0.19)	[-0.31, 0.44]	0.74	0.08	(0.19)	[-0.29, 0.45]	0.67	0.96

S14.14 ODYSSEY A - Weight-for-age change over follow-up

Weeks since randomisation	DTG			SOC			Unadjusted Difference*			
	n	mean	(SE)	n	mean	(SE)	mean	(SE)	[95% CI]	p
12	32	0.51	(0.17)	34	0.61	(0.16)	-0.11	(0.23)	[-0.57, 0.36]	0.66
24	32	0.87	(0.20)	34	0.70	(0.19)	0.17	(0.27)	[-0.38, 0.72]	0.54
36	29	0.97	(0.20)	34	0.86	(0.19)	0.11	(0.27)	[-0.43, 0.65]	0.69
48	28	1.23	(0.21)	34	0.97	(0.19)	0.26	(0.28)	[-0.30, 0.82]	0.35
60	27	1.17	(0.20)	31	1.11	(0.19)	0.07	(0.27)	[-0.48, 0.62]	0.81
72	26	1.40	(0.21)	28	1.27	(0.21)	0.13	(0.30)	[-0.47, 0.73]	0.67
84	29	1.36	(0.18)	33	1.27	(0.18)	0.09	(0.26)	[-0.43, 0.60]	0.73
96	29	1.30	(0.18)	32	1.27	(0.18)	0.03	(0.26)	[-0.48, 0.55]	0.89
Average treatment differences through follow-up+							0.09	(0.22)	[-0.33, 0.52]	0.67

*Change calculated using normal regression adjusting for baseline only. Presenting mean change from a baseline of -2.2 in total and -2.2 in A.

**Normal regression adjusting for baseline and ODYSSEY A/B strata.

+Linear mixed models fitted with random intercept and fixed effects for treatment group, study visit and adjustment covariates (baseline Weight-for-age and ODYSSEY A/B strata (total population only))

++ Test of heterogeneity of treatment effect between ODYSSEY A/B

S14.15 Total population - Weight-for-age change over follow-up

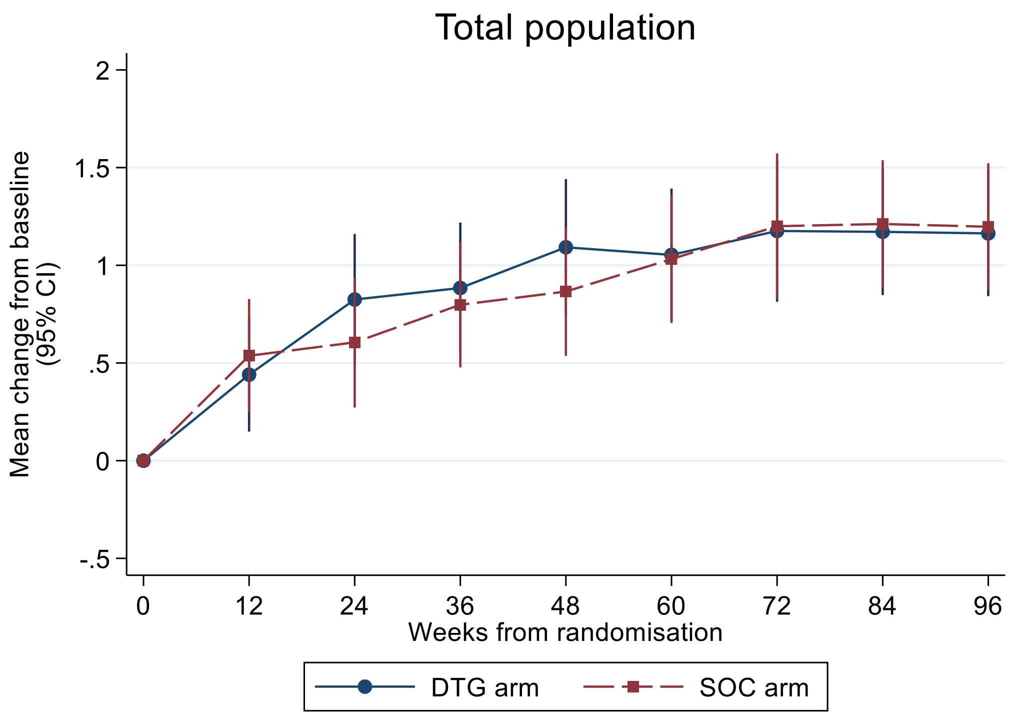

S14.16 ODYSSEY A - Weight-for-age change over follow-up

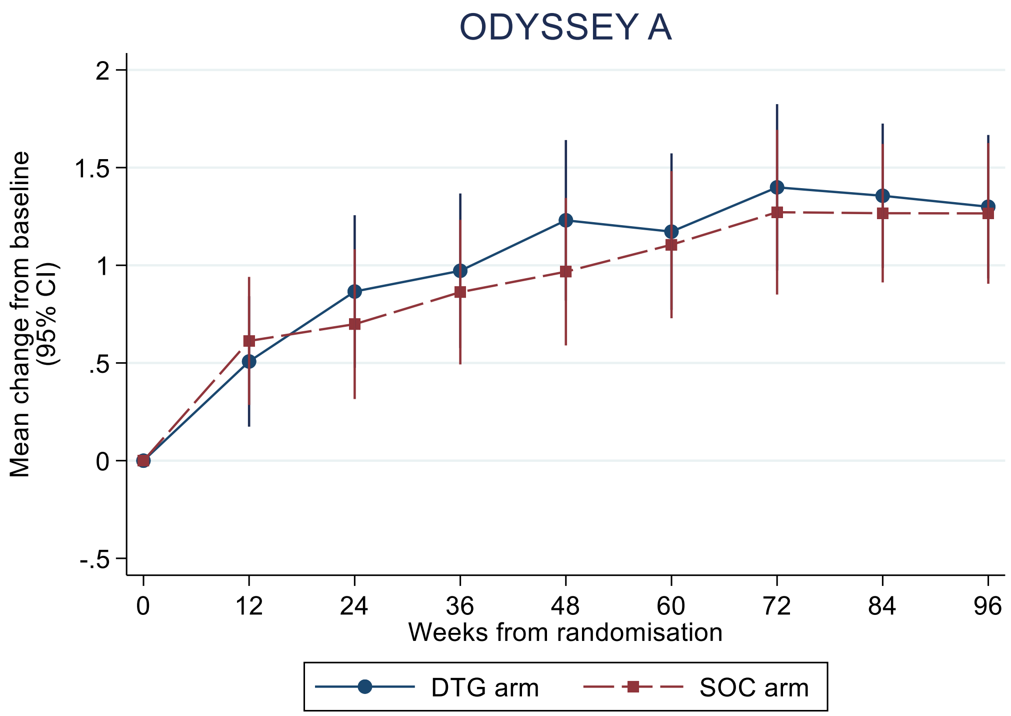

S14.17 Total population - Weight-for-Height change over follow-up

Weeks since randomisation	DTG			SOC			Unadjusted Difference*				Adjusted Difference**				Interaction++
	n	mean	(SE)	n	mean	(SE)	mean	(SE)	[95% CI]	p	mean	(SE)	[95% CI]	p	p
12	38	0.4	(0.2)	40	0.9	(0.2)	-0.5	(0.3)	[-1.0, 0.0]	0.056	-0.5	(0.3)	[-1.0, 0.0]	0.062	0.55
24	38	0.7	(0.2)	40	0.9	(0.2)	-0.1	(0.3)	[-0.8, 0.5]	0.71	-0.1	(0.3)	[-0.8, 0.6]	0.72	0.60
36	34	0.7	(0.2)	38	1.1	(0.2)	-0.4	(0.3)	[-1.1, 0.3]	0.24	-0.4	(0.3)	[-1.1, 0.3]	0.26	0.91
48	34	1.0	(0.3)	38	1.2	(0.2)	-0.2	(0.4)	[-0.9, 0.6]	0.67	-0.1	(0.4)	[-0.8, 0.6]	0.75	0.81
60	31	1.1	(0.3)	35	1.2	(0.3)	-0.1	(0.4)	[-0.9, 0.6]	0.73	-0.1	(0.4)	[-0.8, 0.6]	0.78	0.45
72	30	1.3	(0.3)	30	1.6	(0.3)	-0.2	(0.4)	[-1.1, 0.6]	0.59	-0.2	(0.4)	[-1.0, 0.7]	0.71	0.23
84	33	1.2	(0.3)	33	1.5	(0.3)	-0.3	(0.4)	[-1.1, 0.4]	0.38	-0.2	(0.4)	[-0.9, 0.6]	0.63	0.87
96	29	1.0	(0.3)	31	1.2	(0.3)	-0.2	(0.4)	[-1.0, 0.6]	0.56	-0.2	(0.4)	[-1.0, 0.6]	0.67	0.88
Average treatment differences through follow-up+							-0.2	(0.3)	[-0.8, 0.3]	0.45	-0.2	(0.3)	[-0.7, 0.4]	0.49	0.65

S14.18 ODYSSEY A - Weight-for-Height change over follow-up

Weeks since randomisation	DTG			SOC			Unadjusted Difference*			
	n	mean	(SE)	n	mean	(SE)	mean	(SE)	[95% CI]	p
12	31	0.48	(0.21)	34	0.90	(0.20)	-0.42	(0.29)	[-1.00, 0.16]	0.15
24	31	0.77	(0.27)	34	0.97	(0.26)	-0.20	(0.37)	[-0.94, 0.54]	0.59
36	27	0.81	(0.29)	32	1.19	(0.26)	-0.38	(0.39)	[-1.16, 0.40]	0.34
48	27	1.25	(0.30)	32	1.33	(0.27)	-0.08	(0.40)	[-0.88, 0.73]	0.85
60	24	1.30	(0.32)	29	1.27	(0.29)	0.03	(0.43)	[-0.82, 0.89]	0.94
72	23	1.75	(0.34)	25	1.66	(0.33)	0.08	(0.47)	[-0.86, 1.03]	0.86
84	26	1.49	(0.31)	30	1.65	(0.29)	-0.16	(0.42)	[-1.00, 0.69]	0.71
96	24	1.18	(0.32)	28	1.33	(0.30)	-0.15	(0.44)	[-1.03, 0.73]	0.74
Average treatment differences through follow-up+							-0.14	(0.31)	[-0.74, 0.47]	0.66

*Change calculated using normal regression adjusting for baseline only. Presenting mean change from a baseline of -0.9 in total and -1.0 in A.
**Normal regression adjusting for baseline and ODYSSEY A/B strata.
+Linear mixed models fitted with random intercept and fixed effects for treatment group, study visit and adjustment covariates (baseline Weight-for-Height and ODYSSEY A/B strata (total population only))
++ Test of heterogeneity of treatment effect between ODYSSEY A/B

S14.19 Total population - Weight-for-Height change over follow-up

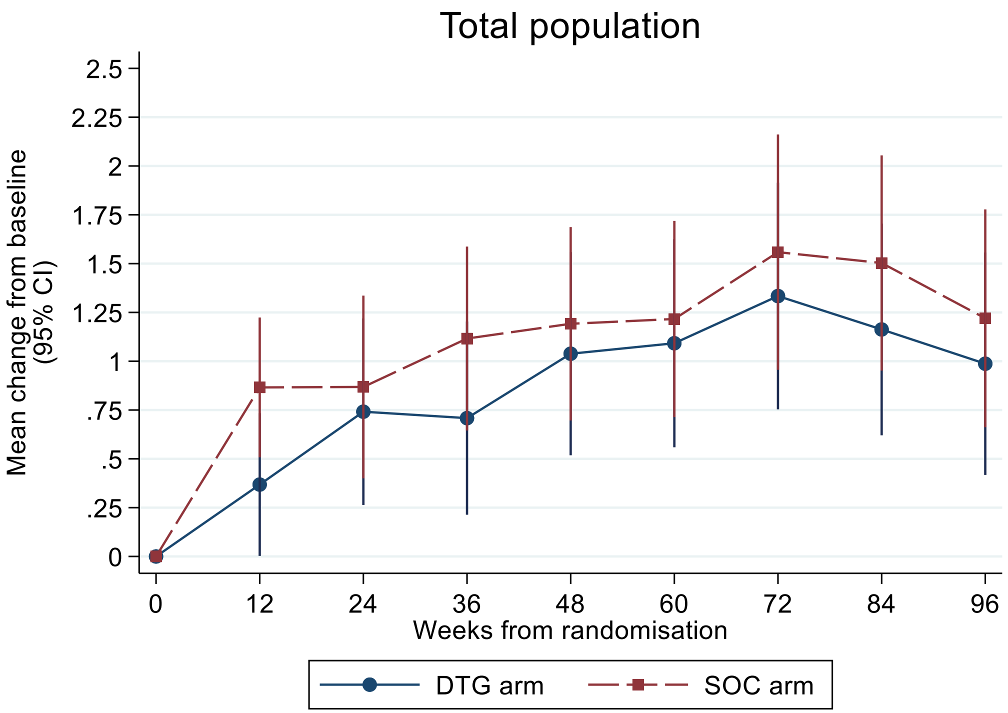

S14.20 ODYSSEY A - Weight-for-Height change over follow-up

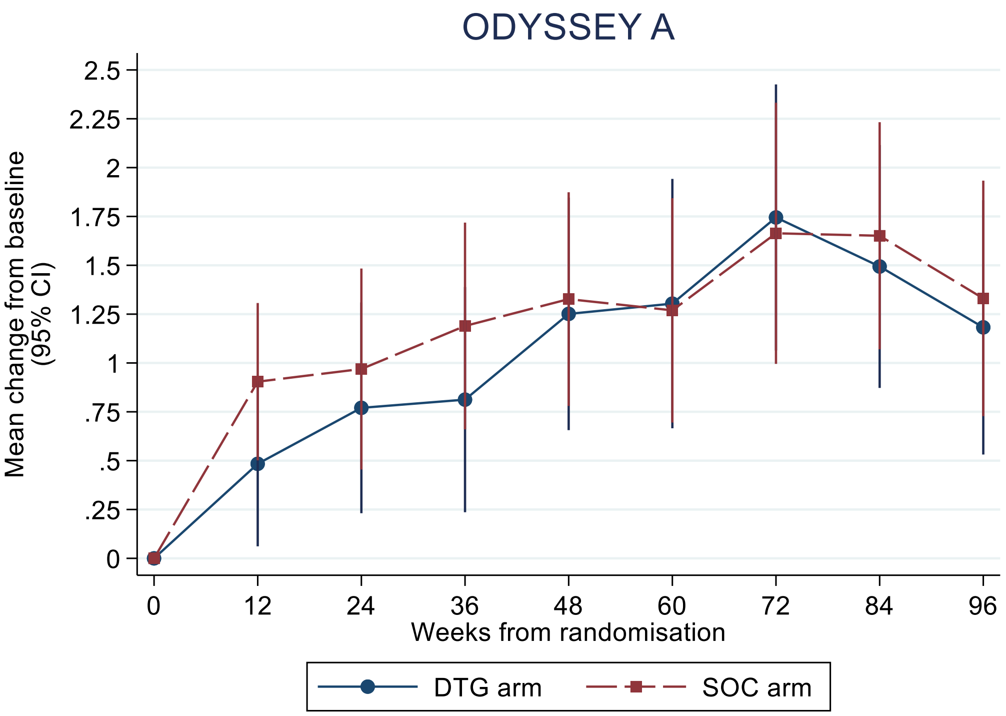

S14.21 Weight-for-age z-scores over follow-up in participants with weight-for-age z-score <-3 at enrolment*

	Total			A		
	**DTG**	**SOC**	**Total**	**DTG**	**SOC**	**Total**
**Participants <-3 at enrolment**	14	13	27	14	11	25
**Week 48**						
<-3	0 (0%)	3 (30%)	3 (14%)	0 (0%)	2 (25%)	2 (11%)
-3-<-2	4 (36%)	3 (30%)	7 (33%)	4 (36%)	3 (38%)	7 (37%)
-2-<0	7 (64%)	3 (30%)	10 (48%)	7 (64%)	2 (25%)	9 (47%)
>=0	0 (0%)	1 (10%)	1 (5%)	0 (0%)	1 (13%)	1 (5%)
Died/LTFU/Withdrawn	3	3	6	3	3	6
**Week 96**						
<-3	0 (0%)	1 (14%)	1 (6%)	0 (0%)	1 (17%)	1 (6%)
-3-<-2	3 (30%)	2 (29%)	5 (29%)	3 (30%)	2 (33%)	5 (31%)
-2-<0	7 (70%)	3 (43%)	10 (59%)	7 (70%)	2 (33%)	9 (56%)
>=0	0 (0%)	1 (14%)	1 (6%)	0 (0%)	1 (17%)	1 (6%)
Died/LTFU/Withdrawn	4	6	10	4	5	9

*Percentages of non-missing

S15 Biochemistry

Note: ODYSSEY B is not presented here due to having few participants

Throughout this section, n refers to the number of participants with available measurement at each visit week. Numbers in models may be lower where there are missing data at baseline.

S15.1 Total population - Creatinine changes over follow-up

Weeks since randomisation	DTG			SOC			Unadjusted Difference*				Adjusted Difference**				Interaction++
	n	mean	(SE)	n	mean	(SE)	mean	(SE)	[95% CI]	p	mean	(SE)	[95% CI]	p	p
4	38	-0.01	(0.02)	39	-0.01	(0.02)	0.00	(0.02)	[-0.05, 0.05]	0.89	0.00	(0.02)	[-0.05, 0.05]	0.87	0.44
24	37	0.03	(0.02)	39	-0.01	(0.02)	0.03	(0.03)	[-0.02, 0.09]	0.25	0.03	(0.03)	[-0.02, 0.09]	0.27	0.16
48	34	0.03	(0.03)	39	0.02	(0.03)	0.01	(0.04)	[-0.06, 0.09]	0.76	0.01	(0.04)	[-0.07, 0.09]	0.80	0.12
72	35	0.03	(0.02)	34	0.03	(0.02)	0.00	(0.03)	[-0.05, 0.06]	0.95	0.00	(0.03)	[-0.05, 0.06]	0.96	0.29
96	36	0.00	(0.02)	36	0.02	(0.02)	-0.02	(0.03)	[-0.07, 0.03]	0.47	-0.02	(0.03)	[-0.07, 0.03]	0.47	0.59
Average treatment differences through follow-up+							0.01	(0.02)	[-0.03, 0.04]	0.66	0.01	(0.02)	[-0.03, 0.04]	0.67	0.79

S15.2 ODYSSEY A - Creatinine over follow-up

Weeks since randomisation	DTG			SOC			Unadjusted Difference*			
	n	mean	(SE)	n	mean	(SE)	mean	(SE)	[95% CI]	p
4	31	-0.00	(0.02)	33	0.00	(0.02)	-0.01	(0.03)	[-0.06, 0.05]	0.85
24	30	0.03	(0.02)	33	-0.02	(0.02)	0.05	(0.03)	[-0.01, 0.11]	0.10
48	27	0.01	(0.03)	33	0.02	(0.03)	-0.02	(0.04)	[-0.10, 0.07]	0.69
72	28	0.04	(0.02)	29	0.03	(0.02)	0.01	(0.03)	[-0.04, 0.06]	0.58
96	29	0.00	(0.02)	31	0.03	(0.02)	-0.02	(0.03)	[-0.08, 0.04]	0.42
Average treatment differences through follow-up+							0.01	(0.02)	[-0.03, 0.05]	0.79

*Change calculated using normal regression adjusting for baseline only. Presenting mean change from a baseline of 0.3 in total and 0.3 in A.
**Normal regression adjusting for baseline and ODYSSEY A/B strata.
+Linear mixed models fitted with random intercept and fixed effects for treatment group, study visit and adjustment covariates (baseline Creatinine and ODYSSEY A/B strata (total population only))
++ Test of heterogeneity of treatment effect between ODYSSEY A/B

S15.3 Total population - Creatinine changes over follow-up

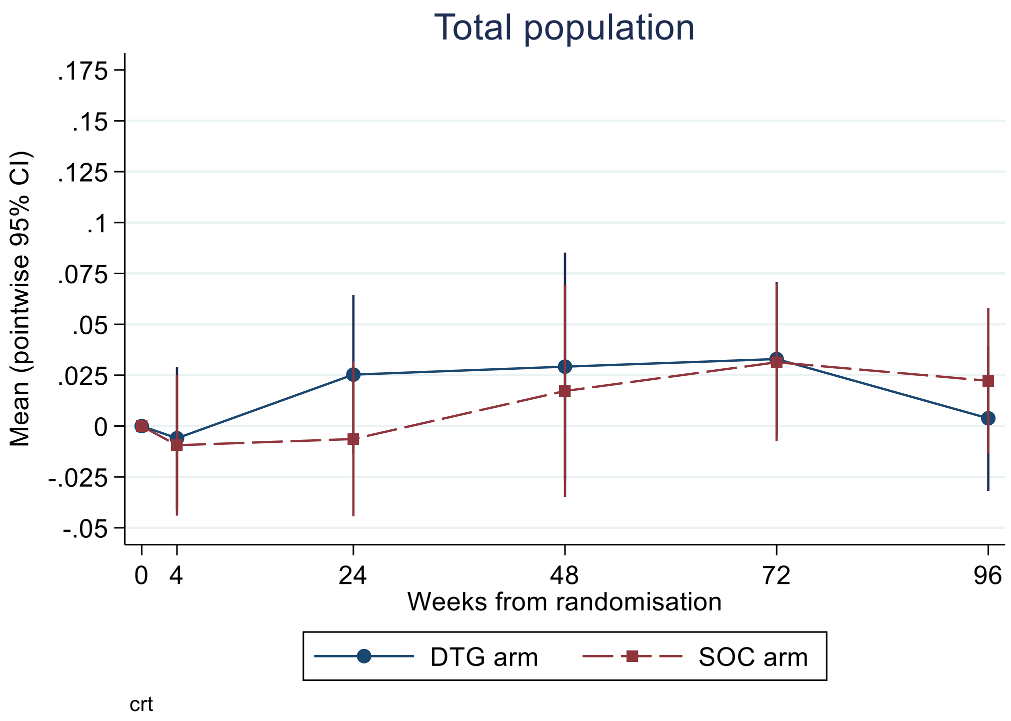

S15.4 ODYSSEY A - Creatinine changes over follow-up

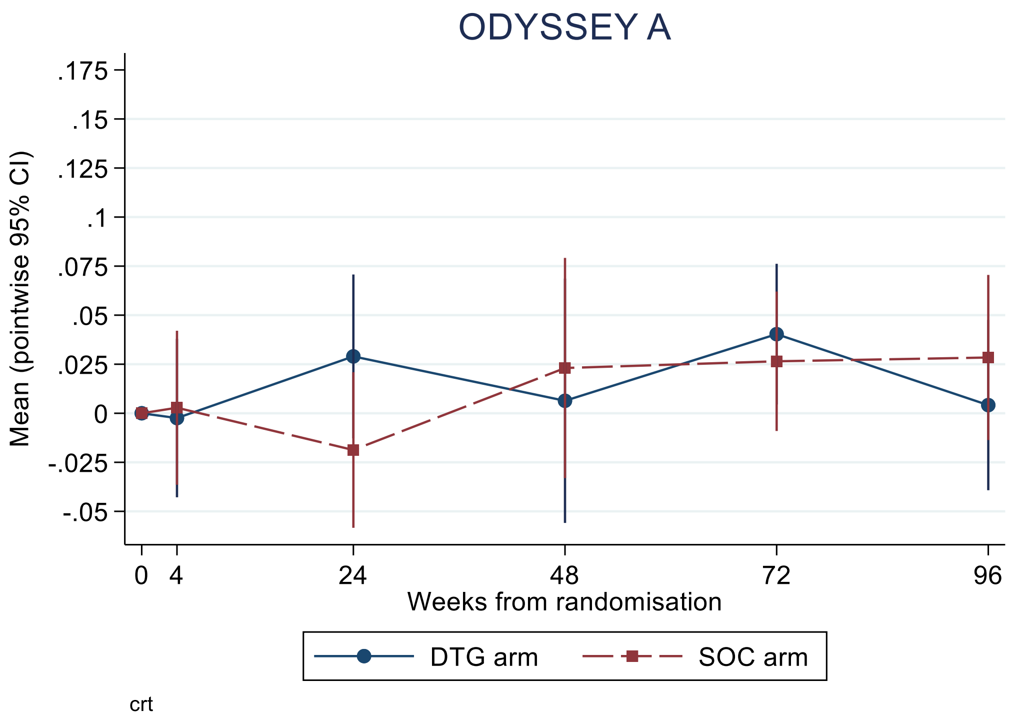

S16 ODYSSEY sites recruiting children to <14kg cohort

University of Zimbabwe Clinical Research Centre (UZCRC), Box A1578 Avondale, Harare, Zimbabwe

Joint Clinical Research Centre (JCRC), Lubowa, Plot 101 Lubowa Estates, off Entebbe road, Kampala, Uganda

Joint Clinical Research Centre (JCRC), Mbarara, P.O Box 40, Mbarara Regional referral Hospital, Mbarara, Uganda

Baylor College of Medicine Bristol Myers Squibb Children's Clinical Centre of Excellence, Block 5, Mulago Hospital, P.O. Box 72052, Kampala, Uganda

Chris Hani Baragwanath Hospital: Perinatal HIV Research Unit, Chris Hani Baragwanath Hospital, P.O. Box 114, Diepkloof, 1864, Soweto, South Africa

Perinatal HIV Research Unit, Klerksdorp/Tsepong Hospital Complex, Matlosana, South Africa

Durban International Clinical Research Site, Parkhome, Gate 1-King Edward VIII Hospital, Umbilo Road, Congella, Durban, KwaZulu-Natal, 4013, South Africa

S17 ODYSSEY inclusion and exclusion criteria

S17.1 Patient Inclusion Criteria

All patients:

- Children ≥28 days and <18 years weighing ≥3kg with confirmed HIV-1 infection*
- Parents/carers and children, where applicable, give informed written consent
- Girls who have reached menses must have a negative pregnancy test at screening and randomisation and be willing to adhere to effective methods of contraception if sexually active
- Children with co-infections who need to start ART according to local/national guidelines
- Parents/carers and children, where applicable, willing to adhere to a minimum of 96 weeks' follow-up

*Children weighing 3 to <14kg must be eligible and willing to participate in the Weight band (WB)-PK1 substudy unless direct enrolment for the child's weight band has opened following the WB-PK1 substudy and/or dosing information has become available from the IMPAACT P1093 DTG dose-finding study.

Additional criteria for ODYSSEY A:

- Planning to start first-line ART

Additional criteria for ODYSSEY B:

- Planning to start second-line ART defined as either: (i) switch of at least 2 ART drugs due to treatment failure; or (ii) switch of only the third agent due to treatment failure where drug sensitivity tests show no mutations conferring NRTI resistance
- Treated with only one previous ART regimen. Single drug substitutions for toxicity, simplification, changes in national guidelines or drug availability are allowed
- At least one NRTI with predicted preserved activity available for a background regimen
- In settings where resistance tests are routinely available, at least one active NRTI from TDF/TAF, ABC or ZDV should have preserved activity based on cumulative results of resistance tests
- In settings where resistance tests are not routinely available, children who are due to switch according to national guidelines should have at least one new NRTI predicted to be available from TDF/TAF, ABC or ZDV
- Viral load ≥500 c/ml at screening visit or within 4 weeks prior to screening

S17.2 Patient Exclusion Criteria

- History or presence of known allergy or contraindications to DTG
- History or presence of known allergy or contraindications to proposed available NRTI backbone or proposed available SOC third agent.
- Alanine aminotransferase (ALT) ≥5 times the upper limit of normal (ULN), OR ALT ≥3xULN and bilirubin ≥2xULN
- Patients with severe hepatic impairment or unstable liver disease (as defined by the presence of ascites, encephalopathy, coagulopathy, hypoalbuminaemia, oesophageal or gastric varices, or persistent jaundice), known biliary abnormalities (with the exception of Gilbert's syndrome or asymptomatic gallstones)
- Anticipated need for Hepatitis C virus (HCV) therapy during the study
- Pregnancy or breastfeeding
- Evidence of lack of susceptibility to integrase inhibitors or more than a 2-week exposure to antiretrovirals of this class
